# Motivational Factors, Physical Activity Impacts, and Sociopsychological Effects of Pokémon GO in Players Over the Years: Scoping Review

**DOI:** 10.2196/89235

**Published:** 2026-07-31

**Authors:** Ghee Kian Koh, Kimberly Mei Yi Low, Shahmir H Ali, Hooi Swang Cheng, Ian Yi Han Ang

**Affiliations:** 1 Saw Swee Hock School of Public Health, National University of Singapore, 21 Lower Kent Ridge Road, Singapore, 119077, Singapore, 65 65164987

**Keywords:** Pokémon GO, physical activity, psychosocial well-being, motivation, mobile health, mHealth, mobile apps, games, exergames, public health

## Abstract

**Background:**

Pokémon GO (PoGo) is a global, location-based augmented reality game integrating virtual play with real-world exploration and social interaction. Its mass adoption and evolving physical and social mechanics make it a salient case to assess whether gamified apps can drive sustained behavior change and deliver health benefits. PoGo’s effects over the years on physical activity (PA), mental and social well-being, and societal impacts remain unexplored by previous reviews.

**Objective:**

This study aimed to map PoGo’s impacts over the years on PA and health, mental well-being, social connectivity, and broader societal outcomes; synthesize motivational factors for initiation, continuance, and disengagement; and assess whether gaps noted in early launch-era reviews are addressed by more recent studies.

**Methods:**

This scoping review followed Arksey and O’Malley’s 5-stage framework and was reported according to PRISMA-ScR (Preferred Reporting Items for Systematic Reviews and Meta-Analyses extension for Scoping Reviews) guidelines. Eligible studies and gray literature examined PoGo’s impacts on physical and mental health and well-being, social and community outcomes, player motivations and notable in-game features, public health applications, and risks. Exclusions included reviews and studies focused solely on technological or augmented reality potential without health or social outcomes. Searches covered August 2016 to December 2025 across Web of Science, ProQuest, PubMed, Scopus, PsycINFO, ScienceDirect, and ProQuest Theses and Dissertations. Eligible papers were screened by multiple reviewers with consensus resolution. The extracted data were charted in Microsoft Excel and synthesized thematically.

**Results:**

A total of 186 sources were included. Twenty-nine studies reported increases in steps, walking or running distances, and outdoor time. The effects were often short-lived, with more sustained gains among older adults and sedentary or overweight young adults. PoGo play was associated with higher life satisfaction, positive affect, reduced distress and loneliness, and a stronger sense of community. Some cognitive benefits were found in adolescents. Social outcomes were reinforced by PoGo-related interactions, as found in 25 studies. PoGo initiation was driven by nostalgia and novelty. Continuance was supported by the satisfaction of needs and regular content updates. Maladaptive drivers were linked to gaming disorder risk and physical strain. Safety concerns in public spaces were found in 17 studies. Data privacy concerns were also identified. Stationary playing may attenuate PA. Societal impacts included suggested public health applications, tourism and mobility effects, and economic benefits tied to existing and potential events and partnerships.

**Conclusions:**

This scoping review assessed PoGo’s impacts through a novel synthesis of PA, mental well-being, and social connectivity research. PoGo is associated with increased walking and reduced loneliness for some demographics, although long-term effects remain unclear. While successful gamified features motivate outdoor activity, concerns exist regarding the associated risks. PoGo demonstrates value for encouraging sedentary individuals toward light PA and social engagement.

## Introduction

### Background

Pokémon GO (PoGo), launched in 2016 by Niantic, Inc, is a groundbreaking augmented reality (AR) mobile game that integrates virtual gameplay with real-world exploration. The game met with instant popularity, with over 508.31 million downloads globally in the quarter of its launch and more than 1 billion downloads globally as of 2025 [1]. PoGo also maintains a monthly global active user base of approximately 60 million players, attesting to its continued appeal across the world, as of August 2025 [2]. Players navigate their surroundings to capture virtual Pokémon, battle in gyms, and interact with in-game features tied to real-world locations, such as PokéStops and community events. Over the years, PoGo has evolved through regular updates, introducing new features such as multiplayer raid battles, AR snapshots, team-based competition, and seasonal events [3]. These updates have expanded the game’s functionality, increased player engagement, and fostered a dynamic global community. PoGo was designed primarily for entertainment, but with an intentional integration of physical activity (PA) and social interaction elements as part of its game design [4].

### Rationale

Unlike traditional PA programs that incorporate social gamification, PoGo’s unique game design and Pokémon’s worldwide appeal could have significant public health impacts by increasing PA and enhancing social connectivity [5-7]. There is an increasing trend in the prevalence of sedentary lifestyles along with its associated health risks, and gamified health interventions are being recommended as an innovative method to engage large numbers of people in PA at a low cost [2,8]. A deeper insight into PoGo’s potential to promote even low- to moderately-intensity PA can be crucial for informing public health interventions and gamification designs targeting reduction in sedentary behaviors [2]. Questions also remain about how its game features drive behavior change and whether such changes can be sustained through long-term engagement as an integration into a more physically active daily living.

Gleaning from the most recent systematic and scoping reviews on the effects of PoGo, research on PoGo has primarily examined its ability to promote PA, showing that the game encourages low- to moderate-intensity PA, such as walking, particularly among sedentary individuals [9,10]. Although increases in step counts have been observed, these gains are typically short-lived and of low intensity, limiting their long-term health impact [10,11]. PoGo’s location-based features also foster social interactions and community engagement by bringing players together in shared spaces. While these aspects enhance its appeal, the influence of socialization on sustained gameplay and associated health outcomes is not well understood, highlighting the need for further exploration of its broader societal impact, including its role in fostering community well-being.

In addition to physical and social outcomes, PoGo has demonstrated potential benefits for mental and cognitive health. Studies included in the most recent systematic review suggest that the game enhances life satisfaction, reduces psychological distress, and supports well-being, while also improving cognitive functions such as working memory and attention [11]. However, the mechanisms driving these effects remain unclear, and little research has been conducted to identify which specific features of the game contribute to positive or negative health and social impacts. This gap is particularly relevant as PoGo continues to evolve and introduce increasingly complex features, potentially shaping player behavior and outcomes in new ways. Given that the most recent systematic review assessing the effects of playing PoGo on PA and psychosocial outcomes was in 2021, these impacts from the game’s development and progression over the past 5 years have not been documented or assessed [11]. In addition, the year 2026, when this scoping review was written, marked the tenth year since PoGo was released. Understanding the psychosocial factors that motivate players to start, continue, or stop playing the game, as well as assessing its broader societal and economic impacts, remains critical to fully realizing its potential as a public health tool and informing future gamified health interventions.

### Objectives

This scoping review aimed to provide a comprehensive assessment of PoGo research until 2025 as a health behavior change tool by examining its holistic impact on physical and mental health, well-being, and societal engagement after nine years of operation. In addition to synthesizing existing research, this review aimed to explore the motivational factors that drive individuals to start, continue, or stop playing PoGo and evaluate the specific game features that contribute to its positive or negative health and societal impacts. By analyzing the successes and limitations of PoGo from a public health perspective, this scoping review seeks to offer valuable insights into optimizing gamification strategies for future public health initiatives and behavior change interventions.

## Methods

### Overview

Given the broad spectrum of knowledge on the effects of playing PoGo and analyses of its numerous game features, as well as widespread media coverage of the game, a scoping review was chosen to provide an overview of the literature. A scoping review allows researchers to explore a research topic comprehensively by identifying literature gaps, including diverse study designs and sources, maintaining flexibility, and maximizing time and resource efficiency [12]. In this review, we adopted the 5-stage framework by Arksey and O’Malley [13]. We reported findings according to the PRISMA-ScR (Preferred Reporting Items for Systematic Reviews and Meta-Analyses extension for Scoping Reviews) guidelines [14], thus ensuring the quality of this review [15]. However, our team did not perform a formal quality appraisal on the included sources, as the objective of scoping reviews is to map the extent of knowledge in the literature, as Arksey and O’Malley [13] suggested.

### Specifying the Research Questions

The primary research question guiding this review was: “Have the areas of further research from game-launch era identified by the latest reviews been covered in more recent studies?” The research questions were also formulated with reference to the findings and comments on research gaps highlighted by the 3 recent reviews mentioned above [9-11]. Six secondary questions were further formulated to delve into specific areas of the primary question and served as a structure for collating the results:

1. What are the demographics of the main player-base from the past until today?
2. Are there benefits to physical and psychological health? In what ways and to what extent?
3. Are there benefits when applied in public health interventions?
4. Are there other societal benefits that tie, in to sustained interest in the game?
5. Are there substantial health risks or other risks (eg, privacy)?
6. What are the key factors in-game (eg, motivation, interest, addiction, fear of missing out) and externally (eg, friends and family) that led to continued playing today?

### Information Sources

All database searches were last conducted on April 15, 2026, according to the PRISMA-S (Preferred Reporting Items for Systematic Reviews and Meta-Analyses Search Reporting Extension) [16]. We identified subject-specific databases most suited to our research question based on a timeline from August 2016 to December 2025, since PoGo officially launched in August 2016. Quantitative and qualitative studies, as well as gray literature on PoGo, were included in this review. The search was conducted in the following seven databases: Web of Science, ProQuest, PubMed, Scopus, PsycINFO, ScienceDirect, and ProQuest Theses and Dissertations. The databases selected and key search terms were informed by a prior systematic review [11]. This scoping review used relevant keywords pertaining to PoGo, physical and mental health and well-being, social relatedness and community, and search terms for motivation and needs satisfaction.

### Search

To identify studies on PoGo play and its impact on physical and mental health, well-being, and societal engagement, we formulated search strategies with our research team (Multimedia Appendix 1). The key search terms were also informed by a prior systematic review [11]. We tested the derived search strategy in PubMed and Web of Science and found the keywords and index terms suitable for use in the other databases. A gray literature search using the same search strategy was conducted to provide a comprehensive understanding of the review’s aims and objectives [17].

### Eligibility Criteria

Papers were deemed eligible for title and abstract screening if they had discussed PoGo use or its applications, as well as its benefits or adverse impacts on physical, mental, or social health and well-being. Only publications in English or officially translated to English were included. From the included papers, this scoping review mapped outcomes related to PoGo’s impacts on overall health and well-being, PoGo engagement and disengagement, and societal impacts globally. Conversely, review papers, such as scoping reviews, systematic reviews, and meta-analyses, were excluded. Analyses solely on the technological aspects of PoGo or the general potential of AR were also excluded, as they did not assess the impact on health and social aspects from playing PoGo.

### Data Items

Where appropriate, we applied Boolean terms and truncation to maximize the retrieval of relevant papers. Salient aspects considered for study inclusion were as follows: PoGo’s impact on PA and physical health; PoGo’s impact on psychological or mental health and well-being; analysis or discussion on key aspects and in-game features that led to continued playing of PoGo; social or commercial impact of PoGo that maintained interest and success for continued playing, including financial or business reviews; how PoGo has been or can be applied or adapted in health interventions and its limitations; aspects of PoGo that make it successful, considering newer features in recent years; and potential health risks or other risks.

### Selection of Sources of Evidence

Upon retrieval from the databases, the studies were uploaded to EndNote (version 20, developed by Clarivate), where duplicates were electronically removed. Thereafter, the studies were exported to the Covidence platform for further manual checking and removal of remaining duplicates. This was followed by title and abstract screening of the remaining studies by 3 independent reviewers in our team (GKK, HSC, and SHA). Studies not meeting the eligibility criteria were removed, while those deemed eligible by at least 2 reviewers (GKK and HSC) were passed to the third reviewer (SHA) for further examination and decision. Any discrepancies in study selection were resolved through consensus with the senior author monthly during the entire review period. Additional studies with eligible titles and abstracts were retrieved for similar full-text screening, and discrepancies were likewise addressed by consulting the senior author.

### Data Charting Process

A data-extraction table was developed iteratively on Microsoft Excel based on the research questions of this review. The tabulated information included the authors, countries, year of publication, study designs, paper types, date of data collection, study population, methodology, interventions, comparisons, outcome measures, key findings, and the main category of the findings according to the research questions. Pilot testing of the table was conducted independently by the first and senior authors based on the first five papers. Subsequently, GKK independently extracted, summarized, and charted data for the individual included sources of evidence. SHA checked all data, and then it was counter-checked by GKK. Key extracted data are provided in Multimedia Appendix 2 [2-7,11,18-188].

### Synthesis of Results

Once the data had been extracted from all the included papers, thematic analyses were conducted [13]. Through thematic organization, the impacts of playing PoGo were grouped into various intervention types and subtypes. Based on the extracted data from the included studies, main findings were identified and grouped accordingly. Guided by the primary and secondary research questions, themes and subthemes for the results section were formed through further in-depth analysis and categorization of each study’s findings. The finalized subthemes and themes were rearranged to ensure that the research questions were answered and the overall findings were coherent. GKK undertook the synthesis of extracted data, and KMYL collated the themes and subthemes into writing. Themes, subthemes, syntheses, and writing were discussed with all authors throughout the process.

### Ethical Considerations

As this study is a scoping review of publicly available literature and does not involve human participant recruitment or identifiable private data, ethics approval was not required in accordance with the National University of Singapore Institutional Review Board. In accordance with current methodological guidance (eg, PRISMA-ScR, JBI Manual), prospective registration is not required for scoping reviews. Any included gray literature will be handled in accordance with applicable copyright and access conditions, and sources will be cited appropriately.

## Results

### Catching Them All: Search Results

**Figure 1.**
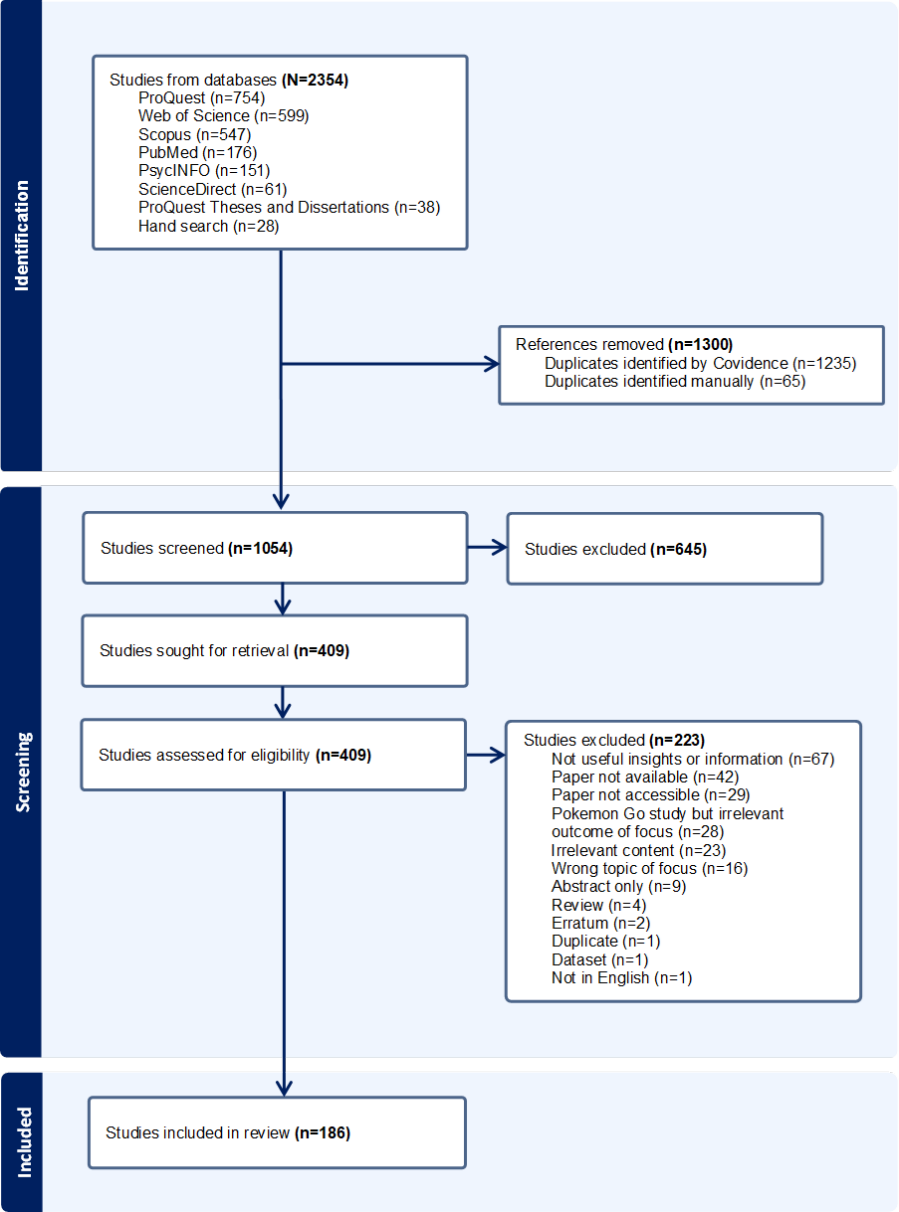
PRISMA (Preferred Reporting Items for Systematic Reviews and Meta-Analyses) flow diagram of study selection.

As provided in Figure 1, the systematic search yielded 2354 publications. These publications were uploaded to Covidence (Veritas Health Innovation) for assessment of eligibility [189]. Covidence is a web-based collaboration software platform that streamlines the production of systematic and other literature reviews. Duplicates and corrigenda were removed, leading to a remainder of 1054 papers. Upon title and abstract screening, 409 papers were sought for retrieval of their full texts. Another 223 publications were removed during full-text review due to ineligibility based on the criteria, or because their full texts were unable to be retrieved. Finally, 186 papers were identified as papers for inclusion in this systematic review. Table 1 is a tabular presentation of the overall characteristics of included studies. The third section “types of studies” and final section “domains of analysis” contain categories that are not mutually exclusive.

**Figure 2.**
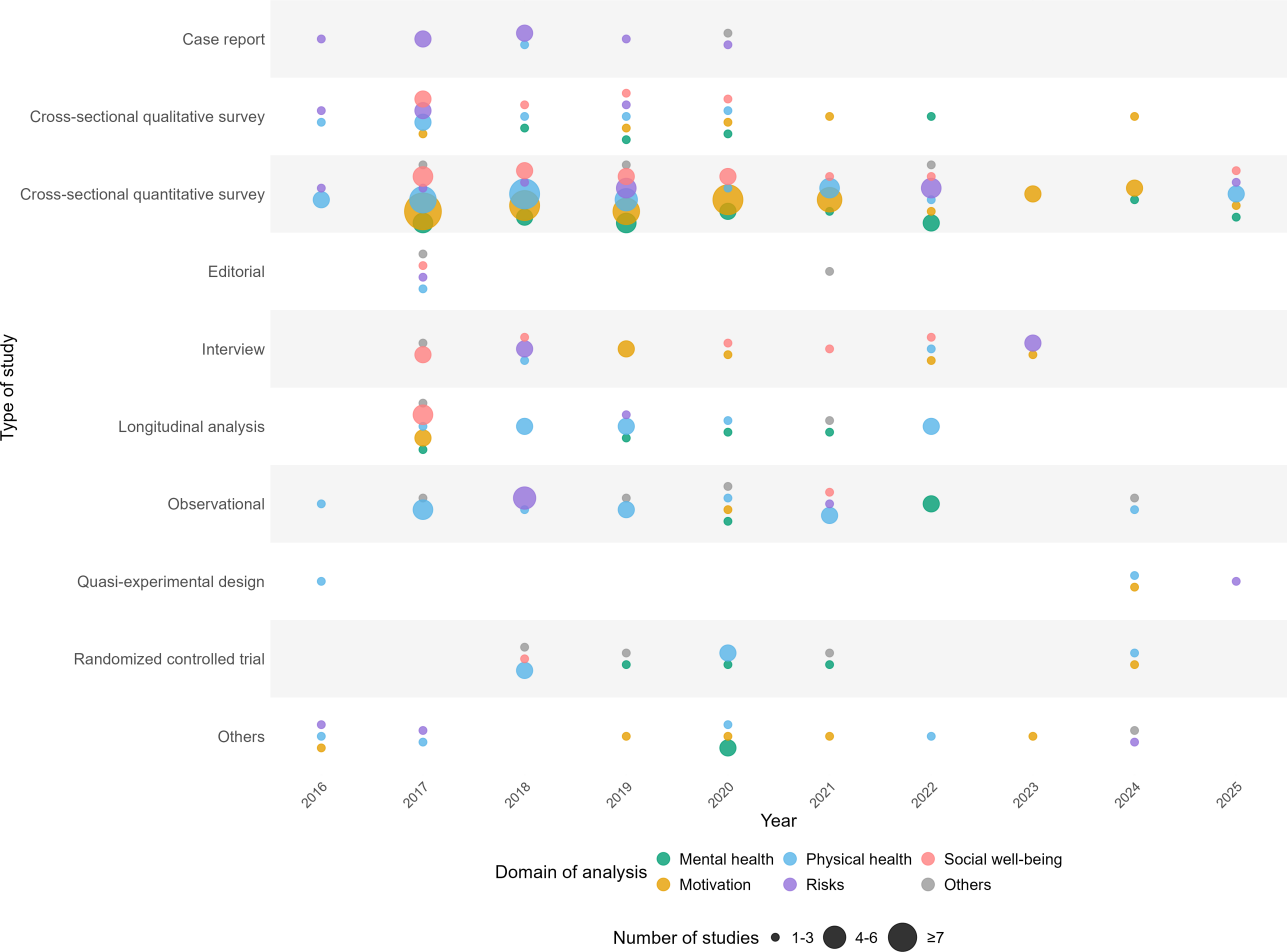
Gap map for the included sources of evidence.

In addition, Figure 2 provides a gap map, which visualizes gaps in the literature from the included sources of evidence. The size of each “bubble” represents the number of sources for each type of study in a particular year and for a particular domain. The sources of evidence can be classified as more than one type of study and/or covering more than one domain of analysis. For type of study, the “Others” label includes diary study, grounded theory, interrupted time-series design, mixed methods analysis, video analysis, brief report, and internet user analytics. For domain of analysis, the “Others” label includes cognition, public health, and societal impact.

**Table 1.** Characteristics and data charting of included sources of evidence.

Parameter	Results
Number of publications by year	2016: 19 2017: 40 2018: 31 2019: 27 2020: 22 2021: 15 2022: 14 2023: 5 2024: 8 2025: 5
Types of sources	Conference proceedings: 16 Newspapers: 13 Peer-reviewed papers: 146 Peer-reviewed journal commentaries: 5 Theses and dissertations: 8
Types of studies^a^	Brief report: 2 Case report: 7 Cross-sectional qualitative survey: 14 Cross-sectional quantitative survey: 91 Diary study: 1 Editorial: 5 Grounded theory: 1 Interview: 13 Internet user analytics: 4 Interrupted time-series design: 1 Longitudinal analysis: 13 Mixed methods analysis: 1 Observational: 21 Quasi-experimental design: 4 Randomized controlled trial: 7 Video analysis: 1
Populations identified^a^	Adolescents: 5 Adults: 9 Children: 5 General public: 20 Older adults: 1 Parents and/or caregivers: 1 Patients: 3 Pokémon GO players: 94 Tertiary education students: 16 Working professionals: 3 Young adults: 3
Domains of analysis^a^	Cognition: 4 Mental health: 31 Motivation: 62 Physical health: 63 Public health: 1 Risks: 42 Social well-being: 29 Societal impact: 7

a These categories are not mutually exclusive, and publications can be classified as more than one type of study, studying more than one population, and/or covering more than one domain of analysis.

### Who Wants to Be the Very Best: Player Demographics

Since the launch of PoGo, player demographics (ie, gender, age, socioeconomic status, personality traits, and so on) have grown in diversity over the years, influenced by factors including but not limited to trends, peer influence, and launch of new game features. Nevertheless, across various geographical locations and studies, ongoing active players were most consistently found to be predominantly males living in urbanized cities [18-20]. Younger players aged around 18-25 years tended to be students or individuals who were more likely to play a game while walking [21-24], and the majority of adult players, aged around 25-60 years, were educated, employed, secular, and possessed middle to higher incomes [23,25].

Significant variability was observed in the aspect of players’ personality traits across gender and age groups. For example, a study in Italy revealed that early users (ie, players who have remained active since shortly after PoGo’s release) tended to be more introverted and identified more strongly as “video-gamers” instead of “social-network or mobile users.” These players also displayed higher levels of agreeableness, resilience, perseverance, and conscientiousness compared to the broader PoGo player population [26-28]. Contrastingly, Kaczmarek et al [20] and Marquet et al [24] highlighted players who demonstrated extraverted traits, as they were particularly curious and enjoyed social opportunities brought about by PoGo. Interestingly, active players had diverse gaming experiences, ranging from individuals with no prior exposure to gaming or AR games, or both [22], to long-time fans of Pokémon card and video games [24], which in turn may have affected their playstyles.

### Pokémon “Go”: What Gets People Into the Game?

Nostalgia emerged as a primary motivation for many players to initially engage in PoGo, as identified and analyzed in 36.2% (n=17) of the 47 studies that investigated the motivations and intention to play among PoGo players. This was particularly relevant for those with prior exposure to the Pokémon franchise, especially those aged 20‐26 years in 2024, stemming from positive experiences with the franchise’s related animations, video games, and card games, which peaked in popularity during the late 1990s and early 2000s [23,29]. For others, commonly reported drivers were curiosity and excitement sparked by the novelty and popularity of PoGo, expectations of enjoyment, fun, and pleasure derived from both the nature and mechanics of the game [21,29-35], keeping up with the trends to stay connected, as well as social influences through peer recommendation to play or a perceived common activity for family bonding [36,37].

Notably, gender and prior gaming experience were covariates found to influence an individual’s motivation and intention to play. Females were more likely to engage in PoGo with the intention of exploring new physical locations, contributing to both health benefits and pleasure [23]. Males were often more driven by nostalgia; they desired to unlock more achievements in the game and be seen as competent and successful in-game, as well as to increase opportunities for social interaction [23]. Moreover, individuals with less or no experience in PoGo gaming were more inclined to play if they believed they could achieve satisfaction in aspects of autonomy and competence through gameplay [38].

### Pokémon “Going”: What Keeps People Going in the Game?

Some of the motivations to engage in PoGo also served as facilitators to continue gameplay, albeit in a different order of priority and relevance across time and types of players [39]. For example, factors relevant to nostalgia, such as franchise and brand loyalty, and fulfilling childhood fantasies, were still positively associated with gameplay continuance across the diverse player base [40-42]. However, this motivation was found to generally weaken within 3 months after the global launch of the game [31,43]. Instead, a significantly greater number of studies (n=35) highlight the lasting importance of both positive and negative motivations for gameplay continuance to be demonstrated.

#### Positive Intrinsic Motivations

In-game competence satisfaction was commonly associated with game progression and achievements, such as gaining mastery through leveling up and progressing in the game [29,44]; sense of glory and accomplishment reportedly derived from competition or challenging others [11,25,40,42,43,45-47], as well as development of competitive skills [43]. Customizable PoGo features, including costumes for in-game avatars, choice of Pokémon companion, choice of game narrative, and ease of application usage to learn and navigate the game [3,40,44,48-50], reportedly satisfied players’ sense of autonomy, which, together with competence satisfaction, were found to predict gameplay continuance as they correlated to game enjoyment [50]. Self-reported “hardcore” PoGo players expressed more positive gratification for enjoyment and competition, while “casual” PoGo players were more positively gratified by the aesthetic and socialization aspects of the game [51]. Of note, male players indicated that they were more driven by competence and relatedness satisfaction compared to their female counterparts, who were strongly motivated by autonomy satisfaction [49]. This was supported by documentation of males investing significantly more time and effort than females in playing to accumulate game points and leveling up [52-54].

Relatedness satisfaction was a key source of motivation associated with continued PoGo engagement across diverse types of players. For example, the nature of PoGo gameplay fueled a “need-to-collect” desire, from which they derived pleasure and fun through the process and mechanisms of collecting Pokémons and rare game items [31,44]. Moreover, PoGo uniquely blended reality with the virtual world, facilitating enjoyment through immersion and flow [33,190]. Coupled with appreciation for the authenticity of game graphics and aesthetics, this integration assisted in fostering meaningful connections between players and specific locations often encountered in their daily lives and likely encouraged continued engagement with the game [55-57]. Players with more social playstyles were found to be encouraged to continue gameplay after deriving intrinsic rewards from establishing new relationships [45,57,58]. The location-based technology also facilitated the strengthening of existing social ties [57,59] and bridged generational and cross-cultural gaps [60]. Forming new friendships from face-to-face interactions [61], as well as developing a sense of belonging through experiencing a shared passion for the game were also crucial motivators for these players [31,57].

#### Positive Extrinsic Motivations

Players who initially played PoGo for recreational purposes remained engaged due to external gratifications gained either directly or indirectly while playing the game. Social benefits were found across numerous studies (n=27) to be a major source of motivation, with some players appreciating opportunities to maintain existing social interactions among peers and family, while others were compelled to forge new friendships through physical encounters or online connections via in-game network externalities. Regardless of their nature, social interactions and interpersonal influences were found to significantly enhance players’ experiences of gamification, thereby encouraging continued gameplay [62]. Other commonly reported motivations included temporary escapism from stressors [25,45,46,63], increased opportunities for outdoor and physical activities that consequently enhanced health [35,40,43,63], and a fun way to alleviate boredom [43,64].

In comparison, players who originally played PoGo due to nostalgia, interest in the novelty of AR games, or prior gaming experience were more motivated by content gratifications such as in-game rewards, incentives (eg, unlocking new content and game progression), and gratification through external recognition after attaining achievements [23,29,34,44]. Additionally, technical features that enhanced the PoGo gameplay experience, such as cooperative modes, location-based game mechanics, and immersive interactions, appealed to players [62,65-67]. Being easy to learn and play PoGo also encouraged continued use [48]. Regular content updates in the form of new Pokémon to collect and exclusive in-app events were also found to be prominent motivators associated with playing and exercising outdoors, especially to get players to stay engaged or be periodically reengaged with PoGo in the long run [31,37,68].

#### Maladaptive Motivations of Sustained Gameplay

A fear of missing out (FOMO), often heightened through social and media influences [62,65], was occasionally found to fuel PoGo gameplay negatively. Moreover, young adults, players with maladaptive gameplay motives—such as an obsessive passion toward PoGo—and those who engaged in PoGo as a means of escapism or coping were more likely to encounter issues with lack of self-regulation and higher impulsivity, resulting in adoption of, and even a greater risk of addiction to virtual gaming as a whole [18,27,63,64,69]. Overly inflating perceptions of gameplay usefulness and excessive in-app purchases were also found to tip the balance toward irrational risk-benefit analysis by individuals, contributing to reported negative drivers of sustained gameplay [34,191].

### Pokémon “Gone”: What Deters People From the Game?

For many, disengagement from PoGo resulted from the gradual erosion and eventual disappearance of initial motivations that had driven gameplay. Common indicators included waning of interest and increasing sense of boredom experienced by the player and within player networks [37,70], along with a sense of stagnation in gameplay and consequently diminishing intrinsic motivations [66]. The game had also grown to be too complex and unenjoyable for some players who lacked a sense of competence, which was key to intrinsic motivation, according to findings from a randomized controlled trial in a dissertation [71]. According to Vaterlaus et al [35], this decline could be attributed to failure to derive gratification from aspects of nostalgia, novelty, pleasure, achievement, and social recognition, which, when unmet, led to negative feelings and ultimately deterred continued play. Additional deterrents included technical issues such as device compatibility problems, missing in-game features, unreliable navigation and step-tracking, which impacted PoGo’s perceived functionality as a fitness application and its playability [4,35,66,72,73], data privacy and safety concerns [7,35,47,61,74,75], as well as frustrations regarding the high operating demand PoGo placed on players’ devices, namely excessive, large consumption of battery, data, and phone storage requirements [76-78]. Finally, a minority of players attributed disengagement to situational reasons, such as lack of time and personal circumstances beyond the game [7,29,72].

### PoGo “Plus”: Positive Health Outcomes

#### Physical Health

Despite only one study [53] explicitly documenting improvements in players’ cardiorespiratory fitness as a direct physical health outcome from playing PoGo, many others (n=29) reported associated improvements in PA levels and frequency across all intensities (ie, low, moderate, and vigorous), alongside a decrease in sedentary behaviors. These are often presented in the form of increased step counts [79,80], walking, and running distances [81]. In a sample of Croatian players, the average player spent more than double the World Health Organization’s recommendation of 150 minutes of PA per week solely from playing PoGo [82]. Such increases were documented to be occurring subconsciously while enjoying the game, regardless of one’s self-efficacy, as PoGo’s gameplay inherently encouraged players to head out more often and exercise as part of real-world exploration and Pokémon collection [60,68,83,84]. Other contributing features included “Adventure Sync,” which recorded the distance traveled with PoGo’s application running in the phone’s background and daily bonuses [85,86]. Yet several other studies reported a large variability regarding the sustainability and extent of these health benefits experienced. For instance, the associated increase in step counts was temporary or even insignificant for some [80,87-89], but prolonged for others, especially among middle-aged and older players [19]. Nonsignificant increases were also documented for overall PA levels and physical wellness [90-93], which could be attributed to compensatory behaviors resulting in increased calorie intake or reduced engagement in other health-promoting activities [94].

Generally, PoGo-associated physical health benefits were evident across players of all ages [19,95], but may be more pronounced among unemployed male gamers in densely populated areas [96]. Other effective populations also include hospitalized patients, players with mental health challenges [97-99], and sedentary or less physically active individuals, particularly young adult players who were overweight or obese [81,93,96,100,192].

#### Mental Health and State of Well-Being

Across players, frequent PoGo play was found to be positively correlated with overall well-being, life satisfaction, positive emotions, and vitality [24,91,101-106], as well as intellectual and spiritual dimensions of wellness found from a cross-sectional survey in a conference proceeding among 370 PoGo players [91]. These benefits were largely associated with the social and exploratory aspects of gameplay, along with relevant features that facilitated these experiences. A total of 14% of studies (n=26) highlighted how PoGo offered opportunities for increased interactions with both existing networks and new individuals, leading to an enhanced sense of community and belonging, social contentment, and relational gratification.

Increased opportunities for social interactions resulting from increased time spent outdoors among players [20,93,102,104,107] were also positively correlated with social satisfaction, stronger social bonds, and a stronger sense of community [57,101,104,108]. Croatian players reported making new friends and experiencing improved mental health through playing PoGo [82]. Lower levels of loneliness were also found among PoGo players who played to form new relationships [59]. PoGo play was also associated with improved mood [109], lower negative affect [110], and reduced feelings of anxiety and distress [111-113]. This was further supported by a notable decline in internet searches related to depression across several countries [114] and self-harm incidences [115] after the global launch of PoGo. The sustainability of these impacts was reportedly strengthened by PoGo’s light, entertaining, and diverse (eg, individual, cooperative, and competitive) AR game mechanics, which allowed integration into the daily routines of many players, thereby enhancing routines with greater meaning and purpose and eventually encouraging lifestyle modifications of health behaviors [65,101,104,116].

While PoGo contributed to mitigating the effects of external stressors on overall well-being and was associated with reduced psychological distress among players who were working adults [113], adolescent players documented increased levels of attention and concentration [52,54,110], and even creativity [52]. Some aspects of working memory and attention were also found to have improved [110]. They also experienced notable improvements in emotional wellness and benefited from the gamification aspects of PoGo, as they derived enjoyment from gaining a sense of presence, identity, and achievement through visual representations of their individual and team success, aligning with their developmental needs [91,117,118]. This, in turn, encouraged further engagement with PoGo and its community via online and physical means, which may also foster a stronger sense of belonging [101,108].

Contrastingly, notable variation in benefits associated with PoGo use was present among subpopulations with mental disorders. For instance, social connectedness was positively related to PoGo use among Japanese players displaying social withdrawal [119,120] or those with highly depressive symptoms [105]. In particular, PoGo was associated with satisfying needs for competence and social satisfaction among severely depressed players, who were more likely to derive affective benefits from these aspects [105]. The associated beneficial effects of PoGo were also more pronounced among individuals seeking clinical treatment, who reported higher life satisfaction and social functioning abilities [106]. Incidental mental well-being and physical health benefits were also reportedly more pronounced among previous inactive individuals [2].

#### Factors Influencing the Extent of Physical and Mental Health Benefits Experienced

Key factors influencing the extent of physical health benefits gained included frequency, duration, and timing of initiation for each gameplay session, as well as gameplay style [95,121,122]. These were, in turn, shaped by extrinsic factors such as the rate and novelty of game updates, but more notably by intrinsic motivations such as players’ social gaming, achievement, and immersion orientations, which affected their intentions behind playing PoGo. For instance, some players approach PoGo primarily as a game rather than a means for fitness, resulting in observed increases in sedentary or deviant behavior or both, when focused on game-related achievements [92,123,124]. Others intentionally used it for both fitness and entertainment, leading to increased PA levels [20,24,68,90,103,125-127]. However, the intensity and flow of PA was also found to be limited by the need to walk slower and stop frequently to play the game [95,124,128,129]. Some studies also reported no significant increase in overall PA [90-93].

In addition to physical health, the aforementioned intrinsic factors have been shown to positively influence mental health outcomes through associated satisfaction of players’ well-being needs. The drive to use PoGo as a social game was positively related to physical, mental, and social health, while the drive to derive a sense of achievement or immersion from playing PoGo most strongly correlated with physical and mental health outcomes, as reported in a conference proceeding survey analysis of 1190 PoGo players [125]. Environmental stressors, such as those experienced during the COVID-19 pandemic, also impacted the extent of health outcomes experienced. For example, players who faced isolation and distress during that era reported significantly more improvements in mental and physical health following PoGo gameplay [97]. PoGo was also identified as a protective factor during COVID-19 lockdown by players in Romania [59]. Notably, PoGo in-game trainer levels significantly predicted players’ reported sense of belonging to the community where they played PoGo, as well as the odds of meeting someone, visiting a new location, and patronizing a new business [130].

### PoGo “Minus”: Negative Health and Well-Being Outcomes

#### Physical Health Impact

Safety concerns associated with PoGo were the most frequently reported downside across 17 studies, with risks extending beyond PoGo users to those around them. PoGo often served as a distraction or indirectly contributed to distractions among all road users [131-133]. Shortly after the release of PoGo in 2016, 12% of players in the United States were reported in a newspaper to have experienced a form of injury while playing PoGo [134], aligning with increased sightings of reckless road-crossing behaviors and individuals failing to pay attention to their surroundings [131]. Although these distractions did not cause a significant change in the number of incidences of traffic fatalities [135], they led to several serious physical injuries [136-138]. One medical case reported a young male player, engrossed in the game, fell onto a railway track and sustained electrical burns [138]. In another incident, a player suddenly dashed across the road, resulting in a collision with an oncoming vehicle [136]. Besides pedestrians, PoGo also caused distractions among drivers [139,140]. In one report, a driver collided with another vehicle after being distracted by a passenger who was absorbed in the game [131]. Another Japanese driver was arrested for causing a fatal hit-and-run incident while playing PoGo during driving [141]. Based on the findings of a video analysis of 242 user-generated PoGo gameplay videos in a conference proceeding, the issue of crowding or swarming brings disturbance and inconvenience to others in the public space, especially road users, while the issues of bystander response, stampedes, and blockages are associated with a major portion of threatening incidents in parks and leisure areas being a result of AR gameplay [142]. Unsafe crossing behaviors and risk-taking inclinations were more prevalent among PoGo players [143-145].

Individuals who were at increased risk of developing gaming addictions, such as those who adopted maladaptive gameplay motives or have attained high levels of achievement in the game—likely driven by PoGo’s competitive elements such as gym battles, raids, player-vs-player mode—have increased likelihood for poorer physical and mental health [49,146]. These players reported more physical health issues related to vision, increased pain, and fatigue [65,146]. The risks associated with dangerous behaviors in public were also heightened when combined with the immersive nature of AR [147,148]. It is also important to note that although risks could be self-inflicted, players in regions that faced higher incidences of theft, physical violence, and sexual harassment, as well as racial and class-based discrimination were particularly vulnerable to negative health impacts associated with PoGo [149].

Risk-taking behaviors were also demonstrated by PoGo players in the form of data privacy. This was especially prominent among players with low self-efficacy or limited knowledge of privacy protection and were thereby less likely to take precautionary measures or exhibit protection behaviors [74]. Players harbored negative attitudes toward the game’s location-based mechanics, requiring players to share their real-time location to play [74]. Moreover, findings from a dissertation mentioned that achievement-driven players, motivated by the desire for competence satisfaction, were discovered to be more susceptible to privacy risks as they often circumvented game design [61]. Some even reported intentionally exchanged personal information to reap game utility, despite being aware of associated risks [150].

#### Mental Health and Well-Being Impact

PoGo playing was associated with poor psychosocial well-being among US players driven by nostalgia and escapism. Those who played PoGo to initiate new relationships also reportedly experienced greater loneliness [69]. An associated decline in mental health was also more prominent among players who engaged in consistent gameplay. A group of medical students in India reported significantly higher levels of stress, depression, and anxiety after playing four hours a day for at least 2 weeks [147]. In addition, 38% of these medical students who played regularly reported moderate to severe levels of depression, as compared to none in the control group. Between 1% and 9% of core PoGo players—defined as trainer level 25 and above—also met diagnostic criteria for gaming disorder, depending on the measures considered [49]. Among a Polish sample of 243 adult PoGo players, 27% of respondents admitted sacrificing sleep, 20% considered themselves addicted, and more than half exceeded the World Health Organization screen time guidelines based solely upon time spent playing PoGo [2]. Excessive and compulsive use of PoGo among 889 Peruvian secondary school students were also associated with video game addiction and problematic relationships with parents and teachers [151].

### Pokémon “GOing” Beyond: Potential Impact on a Societal Level

#### Public Health Impact

Li et al [152] suggested that health problems were multifaceted and shaped by a range of factors, including behavior, economic forces, and cultural influences. PoGo served as an exemplary model of an easily accessible application that integrates these diverse considerations, effectively addressing public health challenges on a global scale. Through its location-based mechanics and ability to accommodate players with different preferences in gameplay, PoGo granted players the autonomy to decide how, where, and in what style they wished to engage with the game [112,153]. These features were suggested in a thesis to have overcome the limitations of traditional health treatment regimens by offering a self-regulated, nonburdensome form of PA that enhances enjoyment and motivation [112]. As a result, PoGo has been shown to promote PA and may potentially yield positive outcomes in addressing obesity-related diseases [57,154,155] and targeting sedentary lifestyles [93,98,120,152,156,157,192]. Although PoGo’s efficacy may be limited in regions with restricted freedom of movement [158], it remains a valuable resource for others who wish to design gamified, population-level health interventions aimed at promoting PA [5,6,154].

#### Potential Commercial and Economic Impact

Engaging in PoGo for recreational purposes had unintentionally influenced users’ mobility patterns, highlighting its potential to integrate urban landscapes into leisure activities. Findings from the included studies offer valuable insights for long-term infrastructure development and urban planning, as well as public policies aimed at encouraging increased pedestrian traffic, ultimately boosting economic productivity and enhancing walkability [159]. PoGo’s ability to encourage exploration [60] and enhance tourism through country-specific events globally was also recognized [160], owing to its storyline as an innovative extension of the existing Pokémon universe, as well as its social components and AR nature. Consequently, nostalgic sentiments evoked by a well-known brand, as well as gratifications derived from gamifications and relationships, present unique opportunities for policymakers to leverage these factors to influence travel behavior and foster tourism-driven mobility [161]. PoGo also serves as an innovative health intervention model for researchers by combining health behavior promotion, marketing forces, and the influence of popular culture [152].

### Rounding Up the Findings

**Figure 3.**
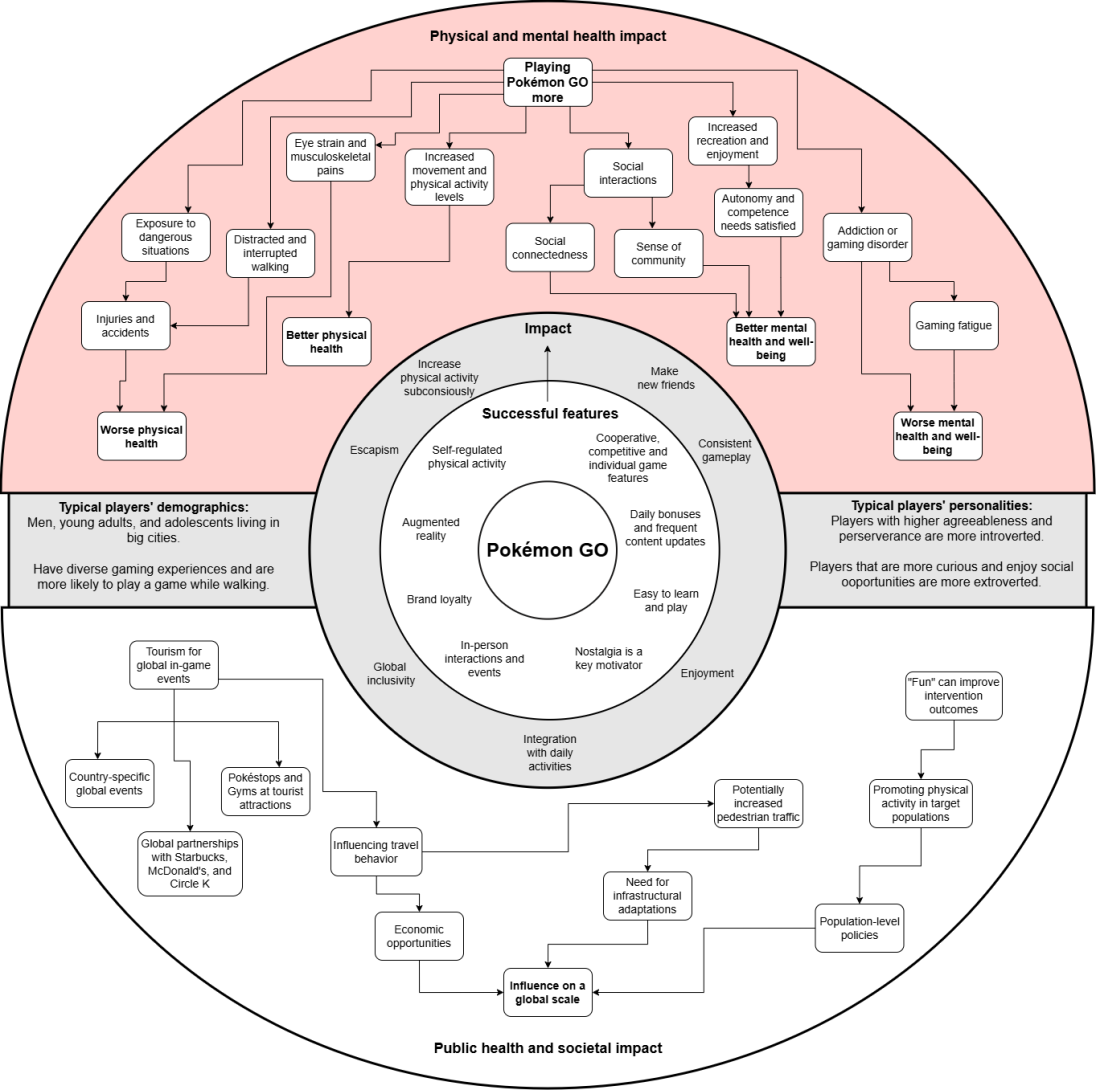
Summary of overall findings from included sources of evidence and opportunities for greater impact.

An overview of the findings from the included sources of evidence, as well as the discussion of the future interventions and implementations based on these findings, are provided in Figure 3. The findings from the included studies span across various theories and models of understanding motivation in the context of gamified health intervention. Motivation for PA has been found to increase with motivation to engage with PoGo use while being physically active. As such, findings include perspectives from individual needs with reference to the self-determination theory, satisfaction with the game with reference to uses and gratification theory, behavioral concepts such as FOMO, as well as analyses on individual differences based on personality and traits. Figure 3 endeavors to visualize these findings holistically and allow consideration of how findings based on various theoretical backgrounds may be interlinked, providing a resource for further exploration of these concepts in relation to one another. The innermost white ring highlights the features and combination of some or all unique features that may contribute to the impacts highlighted in the outer gray ring. The red and white halves further expand on these impacts and are organized into those relating to the individual-level physical and mental health and those relating to broader public health and societal levels.

## Discussion

### Principal Findings

Based on the scoping review’s objectives to assess PoGo as a health behavior change tool, the findings reveal a consistent profile of typical players and mixed outcomes across physical and mental health, well-being, and societal engagement. Active players were predominantly males in urban areas [18-20] and adults with middle-to-higher incomes and education levels [23]. They were reportedly driven by nostalgia for the Pokémon franchise [122], although motivations also differed by gender [49]. PoGo also successfully designed game features to satisfy a diverse range of needs for continued interest in the game [42-50]. While PoGo was found in numerous included studies to be positively associated with increased PA [79-81,156], particularly among sedentary individuals [81,100], its effectiveness as a sustained PA intervention is limited. Players often paused exercise for gameplay, and PA gains frequently diminished after stopping play [95,124,128,129]. One of the game’s strongest impacts lay in its association with increased social connections and community engagement through cooperative features and large-scale events [55-57,62]. However, significant concerns emerged around public safety risks from distracted play and traffic accidents arising from playing PoGo in public [143-145], as well as potential gaming addiction driven by competitive features and FOMO [63].

Key to PoGo’s notable success in gamification of PA is the game’s ability to increase the user’s PA subconsciously while they move around through enjoying the game [60,83]. However, PoGo functions as a game more than a gamified health application, and ultimately, players may find less strenuous means of playing the game efficiently. From the included sources of evidence, players have been found to prioritize gameplay over consistent PA [95,124,128,129]. In recent updates, the extended ability to use “Remote Raid Passes” in more types of raids—allowing players to participate in these raid events from the comfort of their homes—potentially results in little to no impact on PA while playing the game [193]. Ironically, PoGo’s greatest increase in uptake occurred during the COVID-19 lockdown, where the game rose in popularity by adapting to isolated and stationary gaming [59,194]. The need to collect rare Pokémon may end up fueling maladaptive gaming motivations and physical strain from extensive gaming, without any physical health benefits gained, making PoGo no different from other mobile games [68,69,146]. While simply playing the game might be effective in nudging sedentary or overweight adults into engaging in some walking while playing outdoors [81,100], PoGo’s heavy focus on being a game likely impedes its effectiveness as a gamified health intervention tool for sustained increases in PA of various intensities.

PoGo has been understood from the included studies to be a social game designed around cooperation and competition, often in seasonal events involving large groups of players [57,62]. Of the studies included, 25 studies [3,20,24,37,54,61,91,96,98,101,104,105,107,108,111,117,119,127,130,156,162-166] mentioned how PoGo facilitated more interactions between acquaintances and even strangers, potentially enhancing the sense of community, which can improve social well-being. Social support for PA has been found to be a cost-effective public health strategy [195]. The World Health Organization [8] emphasizes community-based recreation as being central to PA promotion across all countries, stating that health policies and programs have a key role in facilitating community-based PA. These findings are also supported by a 6-week field study conducted by Tong et al [196], where social interaction and communication were found to be the most effective gamification features in increasing step count. Those designing new interventions could consider the double benefits of improving social well-being and physical health as encouraged by PoGo’s social features.

Regardless of gender-specific or individually unique motivations, the included studies have shown that various aspects of PoGo, be it competitive, cosmetic, social elements, or simply a desire to “catch them all,” have helped to sustain the interest of PoGo players through meeting their various needs [42-51]. However, time-limited events and the need for the newest rare Pokémon to stay competitive in-game can stimulate a sense of FOMO [62,65], placing players at greater risk of losing self-control and becoming addicted to gaming [63]. Furthermore, PoGo’s video game characteristics, such as sociability features, elements of the flow experience, and general happiness, accounted for 49.2% of the total variance in Game Addiction Scale levels [197]. Increased engagement with PoGo, while associated with higher PA levels and improvements in mental and social well-being, may also result in problematic screen time [2]. As such, even the seemingly positively successful features of PoGo in eliciting more PA, such as increased sociability and enjoyment, should be carefully considered. PoGo use was still found by the included studies to reduce levels of anxiety, loneliness, and negative affect [59,69,110-113]; therefore, the extent of its benefits and potential harms with prolonged use should be further assessed.

PoGo has shown the massive influence of brand loyalty and nostalgia in the adoption of a behavioral change app. This phenomenon has not been replicated by other population-level gamification efforts globally, which highlights the potential of leveraging popular brands in gamified health interventions [1,2]. A successful population-wide incentive-based mobile health PA program is the National Steps Challenge in Singapore, implemented by the Health Promotion Board under the Ministry of Health [198]. Season 3 of the program reached over 14% of Singapore’s adults from 2017 to 2018, engaging 60% of participants for a median of 74 days and increased their step count by 1500 steps daily [198]. In fact, Season 5 of the National Steps Challenge featured Pokémon-themed collectible kits at their roadshows, which attests to the drawing power of Pokémon’s brand [167]. Another 12-week loyalty points-based program implemented in Canada among 32,229 participants saw an increase of more than 6511 steps on average, indicating the effectiveness of incentives in increasing PA [199]. A potential collaboration between governments and PoGo may be able to incentivize increased PA through more PA-focused, cooperative game modes. PoGo’s app can also be enhanced to sync PA from other fitness apps more accurately and comprehensively. The rewards provided could be government-funded, such as retail vouchers, to make participation more meaningful for general or target populations.

Over the years, PoGo has had various successful partnerships and tie-ins with global franchises, such as Starbucks, McDonald’s, and Circle K [200-202]. Companies and even countries have benefited from increased footfall generated by crowds of players who move and gather around commercial and public spaces to play the game, especially when participating in game events. PoGo has also been known to hold numerous events every year in selected cities across the world, such as PoGo Fest, which attract hundreds of thousands of people each time [203,204]. These events encourage not only outdoor movement, but also overseas travel and social interactions beyond national borders. This demonstrates not just PoGo’s value as an effective tourism product, but also its global influence through its appeal and inclusiveness. However, for locations in cities with heavy traffic, such as Los Angeles, significant delays and restricted participation at venues can occur [205]. This may deter PoGo players from participating in country-specific events, thereby reducing the PA impact of such game modes. For cities or countries looking to host such PoGo global events, policymakers may consider investing in long-term infrastructure enhancements to facilitate walkability and smooth exploration of public areas, which will benefit both local and tourist pedestrians. Given that PoGo revenue has maintained above US $557 million after nine years, compared with slightly more than US $550 million in 2016 when it first launched [194], longer-term health and economic impacts can still be leveraged from PoGo. The way these raid events gather masses of people, including tourists, demonstrates PoGo’s effectiveness in gathering physically active and inactive people through a shared interest in the game. However, further long-term research into sustained increases in PA because of playing PoGo is necessary when considering PoGo as a health intervention or when using its successful brand in novel health interventions.

PoGo’s large-scale global events can attract crowds gathering in hundreds, notably in recent Max Battles that can host up to 100 players per virtual lobby [206]. The risks of accidents due to distractions from intense focus on playing the game on mobile phones, unsafe traffic-crossing behaviors, or tourists’ unfamiliarity with foreign public spaces may increase sharply during game event periods [145,147,148]. As such, PoGo may not be suitable as a public health tool in regions that remain unsafe for interacting with strangers [149]. Even in countries that are safe for public social interactions, reducing the risk of serious injuries due to distraction while playing PoGo should be a priority. To leverage community-based PA and encourage tourism, state laws and infrastructure should adapt to accommodate large-scale events, facilitating safe spaces for social interactions and physical activity in public spaces.

### Limitations

This scoping review sought to assess the effects of playing PoGo over the years that the game has been in operation to provide a comprehensive understanding of PoGo’s sustainability and suitability as a gamification tool, as well as its socioeconomic impacts. There are limitations to the extent of our endeavor to understand PoGo’s effects over the years. One key caveat is that the majority (44.1%) of the included studies were published based on player populations and data from the first 3 years of the game’s operation since 2016. Regarding longer-term studies, only 5 [19,60,155,168,169] included studies had a duration of 6 months and above, with only 1 study [169] spanning across 3 years. The longer-term effects of PoGo across its 8-year lifetime cannot be fully understood through studies involving players who have played for more than 3 years, except through comparisons across different player populations examined in later studies. Future studies should focus on longitudinal designs or long-term players with more than 3 years of gameplay experience to better understand changes in health and well-being from playing PoGo.

Another notable observation is the large number of findings being based on cross-sectional surveys. As shown in Table 1, cross-sectional surveys were used in 107 of the 186 included sources. Surveys were also the main mode of data collection consistently used in PoGo research [2]. A large portion of psychosocial outcomes were based upon self-reported scores from questionnaires and online surveys, and study findings were largely correlational among the included studies. While these studies have revealed the depth of PoGo’s impact on players and the larger society, there remains a lack of understanding of how specific elements of PoGo can have causal impact on various areas of a player’s well-being, as well as specific features of the game that can lead to increased motivation for positive health behaviors. Further research studies designed to test the theoretical framework generated from this scoping review would help with better understanding of how to translate the successes and failures of PoGo to gamified health interventions. In addition, this paper only included studies published in English and may have omitted the possibility of useful findings from studies published in other languages. No research librarian or information scientist was consulted for input on the search strategy, which may have limited potential refinements.

### Conclusions

Despite the limitations, this scoping review has achieved its aim of being a novel and comprehensive assessment of existing PoGo knowledge pertaining to promoting and sustaining overall health impacts, as well as the current understanding of its potential as a tool or model for increasing PA at a population level. This scoping review sought to expand the understanding of PoGo’s impact from the most recent findings related to increasing overall health and well-being among its players, which can be applied to designing more effective health intervention tools in the future. Given that the most recent systematic review on a similar topic was in 2021, a review of new PoGo research in the past 4 years was also crucial to understand the impact of newer game features. This scoping review is also the first of its kind that synthesizes outcomes across PA, mental well-being, social connectivity, socioeconomic effects, risks, as well as motivational features and game mechanisms that drive initiation, continuance, and disengagement. This review brings to the field a consolidated and thematically organized understanding of the health aspects whereby benefits appeared most consistently (eg, walking and step increases for some demographics, self-reported improvements to social well-being and reduced loneliness or distress among players), where the evidence is inconclusive or short-lived, and which features (eg, supporting social play, individual needs satisfaction, and game content updates) plausibly explain maintenance vs drop-off. Simultaneously, the review also identified potentially maladaptive complications (eg, gaming disorder risk, distraction injuries, and data privacy trade-offs) that can impede the viability of PoGo or game-based PA tools in general.

In summary, PoGo has been found to relate positively to physical and mental health, as well as social well-being, for various demographics. PoGo also had successful features that motivated players to continue playing, thereby promoting an increase in their PA through enjoying the game. This shows that a strong media franchise and easy-to-play mechanics, together with the right gamified features and AR functionality, can encourage engagement in outdoor PA. The potential cognitive benefits among adolescents also suggest a holistic improvement in overall health from playing PoGo that warrants further investigation. Nevertheless, PoGo functions primarily as a game before being a health intervention tool. Caution should also be exercised against the maladaptive motivations discussed and the playability of PoGo without being physically active. Certain personal and public safety concerns should also be considered due to the AR nature of the game. PoGo certainly has value as a tourism product and the mass appeal to encourage PA within and across countries that policymakers can use in suitable adaptations or partnerships, but its long-term impact on PA and promotion of more vigorous PA remain inconclusive. At the very least, PoGo use has been shown to motivate people who are sedentary, isolated, or experiencing stress and anxiety to go outdoors and engage in some walking and social interactions, which can have a significant impact on improving their physical and mental health, even at a low level of PA intensity.

## Supplementary material

10.2196/89235 Multimedia Appendix 1 Database search strategy.

10.2196/89235 Multimedia Appendix 2 Selected characteristics of included sources of evidence.

10.2196/89235 Checklist 1 PRISMA-ScR checklist.

## Abbreviations

AR: augmented reality
FOMO: fear of missing out
PA: physical activity
PoGo: Pokémon GO
PRISMA-S: Preferred Reporting Items for Systematic Reviews and Meta-Analyses Search Reporting Extension
PRISMA-ScR: Preferred Reporting Items for Systematic Reviews and Meta-Analyses extension for Scoping Reviews
