# Supplementary material for: Motivational Factors, Physical Activity Impacts, and Sociopsychological Effects of Pokémon GO in Players Over the Years: Scoping Review
Source: J Med Internet Res. 2026 Jul 31;28:e89235. doi: 10.2196/89235 (PMC13430953; doi:10.2196/89235)
Supplement: Multimedia Appendix 1 [file jmir-v28-e89235-s001.docx]

| **Database** | **Search Term** |
| --- | --- |
| Web of Science | (TI=(("Pokémon GO" OR "Pokemon Go" OR "Pokemon GO" OR "Pokémon Go" OR "PokemonGO" OR "PokémonGO")  AND  ((exercise* OR "physical activity" OR "physical activities" OR "physical fitness" OR "motor activity" OR walk* OR run OR running OR "physical health" OR sedentary OR "step count" OR "step counts" OR "physical inactivity" OR "physical performance" OR "physical function" OR "physical functioning" OR "body mass index" OR BMI OR obesity OR "weight loss" OR "physical wellbeing" OR "physical well-being")  OR  (psychosocial OR psychological OR depression OR depressive OR anxiety OR anxious OR affect OR mood OR "mental health" OR wellbeing OR "well-being" OR "well being" OR "quality of life" OR QoL OR cognition OR cognitive OR memory OR "executive function" OR "executive functioning" OR "executive functions" OR "cognitive function" OR "cognitive functioning" OR "cognitive performance" OR attention OR stress OR "self-esteem" OR "self esteem" OR happiness OR "life satisfaction" OR emotion*)  OR  ("social health" OR "social wellbeing" OR "social well-being" OR "social relation" OR "social relations" OR "social relationship" OR "social relationships" OR "social support" OR "social connectedness" OR "social connection" OR "social connections" OR "social interaction" OR "social interactions" OR "interpersonal relation" OR "interpersonal relations" OR "interpersonal relationship" OR "interpersonal relationships" OR community OR communities OR "sense of community" OR "community engagement" OR loneliness OR "social isolation" OR satisfaction OR motivat* OR incentive* OR "behavioral engagement" OR "behavioural engagement" OR engagement OR "user engagement" OR "social capital" OR "social network" OR "social networks" OR "social participation" OR "social activity" OR "social activities")  OR  ("health promotion" OR "health behavior" OR "health behaviour" OR "health benefit" OR "health benefits" OR "health risk" OR "health risks" OR "preventive health" OR prevention OR "disease prevention" OR "health intervention" OR "health interventions" OR "lifestyle intervention" OR "lifestyle interventions" OR "behavioral intervention" OR "behavioural intervention" OR "physical activity intervention" OR "physical activity interventions" OR "physical activity promotion" OR "exercise intervention" OR "exercise interventions" OR "exercise promotion" OR cardiovascular OR "cardiovascular health" OR "cardiovascular disease" OR "cardiovascular diseases" OR diabetes OR "metabolic syndrome" OR "metabolic health" OR "chronic disease" OR "chronic diseases" OR "noncommunicable disease" OR "noncommunicable diseases" OR "non-communicable disease" OR "non-communicable diseases" OR "societal impact" OR "societal impacts" OR "social impact" OR "social impacts" OR "societal benefit" OR "societal benefits" OR "societal change" OR "social change" OR "behavioral change" OR "behavioural change" OR "behavior change" OR "behaviour change" OR "lifestyle change" OR "lifestyle changes" OR "lifestyle modification" OR "lifestyle modifications" OR safety OR "traffic accident" OR "traffic accidents" OR "pedestrian safety" OR "road safety" OR injury OR injuries OR "risk taking" OR "risky behavior" OR "risky behaviour" OR "distracted walking" OR "distracted driving" OR privacy OR "data privacy" OR "data security" OR "personal data" OR "data collection" OR surveillance OR crime OR trespass* OR violence OR "social problem" OR "social problems" OR "social behavior" OR "social behaviour" OR "cultural impact" OR "cultural impacts" OR "cultural phenomenon" OR "cultural phenomena" OR "social phenomenon" OR "social phenomena" OR "public space" OR "public spaces" OR "urban space" OR "urban spaces" OR "place making" OR placemaking)))) OR AB=(("Pokémon GO" OR "Pokemon Go" OR "Pokemon GO" OR "Pokémon Go" OR "PokemonGO" OR "PokémonGO") AND ((exercise* OR "physical activity" OR "physical activities" OR "physical fitness" OR "motor activity" OR walk* OR run OR running OR "physical health" OR sedentary OR "step count" OR "step counts" OR "physical inactivity" OR "physical performance" OR "physical function" OR "physical functioning" OR "body mass index" OR BMI OR obesity OR "weight loss" OR "physical wellbeing" OR "physical well-being") OR (psychosocial OR psychological OR depression OR depressive OR anxiety OR anxious OR affect OR mood OR "mental health" OR wellbeing OR "well-being" OR "well being" OR "quality of life" OR QoL OR cognition OR cognitive OR memory OR "executive function" OR "executive functioning" OR "executive functions" OR "cognitive function" OR "cognitive functioning" OR "cognitive performance" OR attention OR stress OR "self-esteem" OR "self esteem" OR happiness OR "life satisfaction" OR emotion*) OR ("social health" OR "social wellbeing" OR "social well-being" OR "social relation" OR "social relations" OR "social relationship" OR "social relationships" OR "social support" OR "social connectedness" OR "social connection" OR "social connections" OR "social interaction" OR "social interactions" OR "interpersonal relation" OR "interpersonal relations" OR "interpersonal relationship" OR "interpersonal relationships" OR community OR communities OR "sense of community" OR "community engagement" OR loneliness OR "social isolation" OR satisfaction OR motivat* OR incentive* OR "behavioral engagement" OR "behavioural engagement" OR engagement OR "user engagement" OR "social capital" OR "social network" OR "social networks" OR "social participation" OR "social activity" OR "social activities") OR ("health promotion" OR "health behavior" OR "health behaviour" OR "health benefit" OR "health benefits" OR "health risk" OR "health risks" OR "preventive health" OR prevention OR "disease prevention" OR "health intervention" OR "health interventions" OR "lifestyle intervention" OR "lifestyle interventions" OR "behavioral intervention" OR "behavioural intervention" OR "physical activity intervention" OR "physical activity interventions" OR "physical activity promotion" OR "exercise intervention" OR "exercise interventions" OR "exercise promotion" OR cardiovascular OR "cardiovascular health" OR "cardiovascular disease" OR "cardiovascular diseases" OR diabetes OR "metabolic syndrome" OR "metabolic health" OR "chronic disease" OR "chronic diseases" OR "noncommunicable disease" OR "noncommunicable diseases" OR "non-communicable disease" OR "non-communicable diseases" OR "societal impact" OR "societal impacts" OR "social impact" OR "social impacts" OR "societal benefit" OR "societal benefits" OR "societal change" OR "social change" OR "behavioral change" OR "behavioural change" OR "behavior change" OR "behaviour change" OR "lifestyle change" OR "lifestyle changes" OR "lifestyle modification" OR "lifestyle modifications" OR safety OR "traffic accident" OR "traffic accidents" OR "pedestrian safety" OR "road safety" OR injury OR injuries OR "risk taking" OR "risky behavior" OR "risky behaviour" OR "distracted walking" OR "distracted driving" OR privacy OR "data privacy" OR "data security" OR "personal data" OR "data collection" OR surveillance OR crime OR trespass* OR violence OR "social problem" OR "social problems" OR "social behavior" OR "social behaviour" OR "cultural impact" OR "cultural impacts" OR "cultural phenomenon" OR "cultural phenomena" OR "social phenomenon" OR "social phenomena" OR "public space" OR "public spaces" OR "urban space" OR "urban spaces" OR "place making" OR placemaking)))  Limits: title, abstract, Not review; 1/1/2016 to 31/12/2025; English |
| Scopus | ("Pokémon GO" OR "Pokemon Go" OR "Pokemon GO" OR "Pokémon Go" OR "PokemonGO" OR "PokémonGO")  AND  ((exercise* OR "physical activity" OR "physical activities" OR "physical fitness" OR "motor activity" OR walk* OR run OR running OR "physical health" OR sedentary OR "step count" OR "step counts" OR "physical inactivity" OR "physical performance" OR "physical function" OR "physical functioning" OR "body mass index" OR BMI OR obesity OR "weight loss" OR "physical wellbeing" OR "physical well-being") OR (psychosocial OR psychological OR depression OR depressive OR anxiety OR anxious OR affect OR mood OR "mental health" OR wellbeing OR "well-being" OR "well being" OR "quality of life" OR QoL OR cognition OR cognitive OR memory OR "executive function" OR "executive functioning" OR "executive functions" OR "cognitive function" OR "cognitive functioning" OR "cognitive performance" OR attention OR stress OR "self-esteem" OR "self esteem" OR happiness OR "life satisfaction" OR emotion*)  OR  ("social health" OR "social wellbeing" OR "social well-being" OR "social relation" OR "social relations" OR "social relationship" OR "social relationships" OR "social support" OR "social connectedness" OR "social connection" OR "social connections" OR "social interaction" OR "social interactions" OR "interpersonal relation" OR "interpersonal relations" OR "interpersonal relationship" OR "interpersonal relationships" OR community OR communities OR "sense of community" OR "community engagement" OR loneliness OR "social isolation" OR satisfaction OR motivat* OR incentive* OR "behavioral engagement" OR "behavioural engagement" OR engagement OR "user engagement" OR "social capital" OR "social network" OR "social networks" OR "social participation" OR "social activity" OR "social activities")  OR  ("health promotion" OR "health behavior" OR "health behaviour" OR "health benefit" OR "health benefits" OR "health risk" OR "health risks" OR "preventive health" OR prevention OR "disease prevention" OR "health intervention" OR "health interventions" OR "lifestyle intervention" OR "lifestyle interventions" OR "behavioral intervention" OR "behavioural intervention" OR "physical activity intervention" OR "physical activity interventions" OR "physical activity promotion" OR "exercise intervention" OR "exercise interventions" OR "exercise promotion" OR cardiovascular OR "cardiovascular health" OR "cardiovascular disease" OR "cardiovascular diseases" OR diabetes OR "metabolic syndrome" OR "metabolic health" OR "chronic disease" OR "chronic diseases" OR "noncommunicable disease" OR "noncommunicable diseases" OR "non-communicable disease" OR "non-communicable diseases" OR "societal impact" OR "societal impacts" OR "social impact" OR "social impacts" OR "societal benefit" OR "societal benefits" OR "societal change" OR "social change" OR "behavioral change" OR "behavioural change" OR "behavior change" OR "behaviour change" OR "lifestyle change" OR "lifestyle changes" OR "lifestyle modification" OR "lifestyle modifications" OR safety OR "traffic accident" OR "traffic accidents" OR "pedestrian safety" OR "road safety" OR injury OR injuries OR "risk taking" OR "risky behavior" OR "risky behaviour" OR "distracted walking" OR "distracted driving" OR privacy OR "data privacy" OR "data security" OR "personal data" OR "data collection" OR surveillance OR crime OR trespass* OR violence OR "social problem" OR "social problems" OR "social behavior" OR "social behaviour" OR "cultural impact" OR "cultural impacts" OR "cultural phenomenon" OR "cultural phenomena" OR "social phenomenon" OR "social phenomena" OR "public space" OR "public spaces" OR "urban space" OR "urban spaces" OR "place making" OR placemaking))  Limits: Article title, Abstract, Keywords; English, no review, limit to “POKEMON GO” 2016-2025 |
| ProQuest Journals & ProQuest Dissertations & Theses Citation Index | ("Pokémon GO" OR "Pokemon Go" OR "Pokemon GO" OR "Pokémon Go" OR "PokemonGO" OR "PokémonGO")  AND  ((exercise* OR "physical activity" OR "physical activities" OR "physical fitness" OR "motor activity" OR walk* OR run OR running OR "physical health" OR sedentary OR "step count" OR "step counts" OR "physical inactivity" OR "physical performance" OR "physical function" OR "physical functioning" OR "body mass index" OR BMI OR obesity OR "weight loss" OR "physical wellbeing" OR "physical well-being") OR (psychosocial OR psychological OR depression OR depressive OR anxiety OR anxious OR affect OR mood OR "mental health" OR wellbeing OR "well-being" OR "well being" OR "quality of life" OR QoL OR cognition OR cognitive OR memory OR "executive function" OR "executive functioning" OR "executive functions" OR "cognitive function" OR "cognitive functioning" OR "cognitive performance" OR attention OR stress OR "self-esteem" OR "self esteem" OR happiness OR "life satisfaction" OR emotion*)  OR  ("social health" OR "social wellbeing" OR "social well-being" OR "social relation" OR "social relations" OR "social relationship" OR "social relationships" OR "social support" OR "social connectedness" OR "social connection" OR "social connections" OR "social interaction" OR "social interactions" OR "interpersonal relation" OR "interpersonal relations" OR "interpersonal relationship" OR "interpersonal relationships" OR community OR communities OR "sense of community" OR "community engagement" OR loneliness OR "social isolation" OR satisfaction OR motivat* OR incentive* OR "behavioral engagement" OR "behavioural engagement" OR engagement OR "user engagement" OR "social capital" OR "social network" OR "social networks" OR "social participation" OR "social activity" OR "social activities")  OR  ("health promotion" OR "health behavior" OR "health behaviour" OR "health benefit" OR "health benefits" OR "health risk" OR "health risks" OR "preventive health" OR prevention OR "disease prevention" OR "health intervention" OR "health interventions" OR "lifestyle intervention" OR "lifestyle interventions" OR "behavioral intervention" OR "behavioural intervention" OR "physical activity intervention" OR "physical activity interventions" OR "physical activity promotion" OR "exercise intervention" OR "exercise interventions" OR "exercise promotion" OR cardiovascular OR "cardiovascular health" OR "cardiovascular disease" OR "cardiovascular diseases" OR diabetes OR "metabolic syndrome" OR "metabolic health" OR "chronic disease" OR "chronic diseases" OR "noncommunicable disease" OR "noncommunicable diseases" OR "non-communicable disease" OR "non-communicable diseases" OR "societal impact" OR "societal impacts" OR "social impact" OR "social impacts" OR "societal benefit" OR "societal benefits" OR "societal change" OR "social change" OR "behavioral change" OR "behavioural change" OR "behavior change" OR "behaviour change" OR "lifestyle change" OR "lifestyle changes" OR "lifestyle modification" OR "lifestyle modifications" OR safety OR "traffic accident" OR "traffic accidents" OR "pedestrian safety" OR "road safety" OR injury OR injuries OR "risk taking" OR "risky behavior" OR "risky behaviour" OR "distracted walking" OR "distracted driving" OR privacy OR "data privacy" OR "data security" OR "personal data" OR "data collection" OR surveillance OR crime OR trespass* OR violence OR "social problem" OR "social problems" OR "social behavior" OR "social behaviour" OR "cultural impact" OR "cultural impacts" OR "cultural phenomenon" OR "cultural phenomena" OR "social phenomenon" OR "social phenomena" OR "public space" OR "public spaces" OR "urban space" OR "urban spaces" OR "place making" OR placemaking))  Limits: 2016-2025; English; Abstract; Document title |
| PubMed | ("Pokémon GO"[tiab] OR "Pokemon Go"[tiab] OR "Pokemon GO"[tiab] OR "Pokémon Go"[tiab] OR "PokemonGO"[tiab] OR "PokémonGO"[tiab])  AND  (("Exercise"[Mesh] OR "Motor Activity"[Mesh] OR "Physical Fitness"[Mesh] OR "Walking"[Mesh] OR "Running"[Mesh] OR "Sedentary Behavior"[Mesh] OR "physical activity"[tiab] OR "physical activities"[tiab] OR "physical health"[tiab] OR "physical fitness"[tiab] OR "motor activity"[tiab] OR exercise*[tiab] OR walk*[tiab] OR run[tiab] OR running[tiab] OR "sedentary"[tiab] OR "step count"[tiab] OR "step counts"[tiab] OR "physical inactivity"[tiab] OR "physical performance"[tiab] OR "physical function"[tiab] OR "physical functioning"[tiab] OR "body mass index"[tiab] OR BMI[tiab] OR obesity[tiab] OR "weight loss"[tiab] OR "physical wellbeing"[tiab] OR "physical well-being"[tiab])  OR  ("Mental Health"[Mesh] OR "Depression"[Mesh] OR "Anxiety"[Mesh] OR "Affect"[Mesh] OR "Quality of Life"[Mesh] OR "Cognition"[Mesh] OR "Memory"[Mesh] OR "Executive Function"[Mesh] OR "Emotions"[Mesh] OR "Stress, Psychological"[Mesh] OR "Self Concept"[Mesh] OR "Happiness"[Mesh] OR psychosocial[tiab] OR psychological[tiab] OR depression[tiab] OR depressive[tiab] OR anxiety[tiab] OR anxious[tiab] OR affect[tiab] OR mood[tiab] OR "mental health"[tiab] OR wellbeing[tiab] OR "well-being"[tiab] OR "well being"[tiab] OR "quality of life"[tiab] OR QoL[tiab] OR cognition[tiab] OR cognitive[tiab] OR memory[tiab] OR "executive function"[tiab] OR "executive functioning"[tiab] OR "executive functions"[tiab] OR "cognitive function"[tiab] OR "cognitive functioning"[tiab] OR "cognitive performance"[tiab] OR attention[tiab] OR stress[tiab] OR "self-esteem"[tiab] OR "self esteem"[tiab] OR happiness[tiab] OR "life satisfaction"[tiab] OR emotion*[tiab])  OR  ("Social Support"[Mesh] OR "Interpersonal Relations"[Mesh] OR "Social Interaction"[Mesh] OR "Motivation"[Mesh] OR "Personal Satisfaction"[Mesh] OR "Loneliness"[Mesh] OR "Social Isolation"[Mesh] OR "Community Networks"[Mesh] OR "Social Networking"[Mesh] OR "social health"[tiab] OR "social wellbeing"[tiab] OR "social well-being"[tiab] OR "social relation"[tiab] OR "social relations"[tiab] OR "social relationship"[tiab] OR "social relationships"[tiab] OR "social support"[tiab] OR "social connectedness"[tiab] OR "social connection"[tiab] OR "social connections"[tiab] OR "social interaction"[tiab] OR "social interactions"[tiab] OR "interpersonal relation"[tiab] OR "interpersonal relations"[tiab] OR "interpersonal relationship"[tiab] OR "interpersonal relationships"[tiab] OR community[tiab] OR communities[tiab] OR "sense of community"[tiab] OR "community engagement"[tiab] OR loneliness[tiab] OR "social isolation"[tiab] OR satisfaction[tiab] OR motivat*[tiab] OR incentive*[tiab] OR "behavioral engagement"[tiab] OR "behavioural engagement"[tiab] OR engagement[tiab] OR "user engagement"[tiab] OR "social capital"[tiab] OR "social network*"[tiab] OR "social participation"[tiab] OR "social activity"[tiab] OR "social activities"[tiab])  OR  ("Health Promotion"[Mesh] OR "Health Behavior"[Mesh] OR "Preventive Health Services"[Mesh] OR "Obesity"[Mesh] OR "Weight Loss"[Mesh] OR "Cardiovascular Diseases"[Mesh] OR "Diabetes Mellitus"[Mesh] OR "Metabolic Syndrome"[Mesh] OR "Accidents, Traffic"[Mesh] OR "Wounds and Injuries"[Mesh] OR "Safety"[Mesh] OR "Risk-Taking"[Mesh] OR "Privacy"[Mesh] OR "Crime"[Mesh] OR "Violence"[Mesh] OR "Social Problems"[Mesh] OR "Social Change"[Mesh] OR "Social Behavior"[Mesh] OR "Cultural Characteristics"[Mesh] OR "health promotion"[tiab] OR "health behavior"[tiab] OR "health behaviour"[tiab] OR "health benefit"[tiab] OR "health benefits"[tiab] OR "health risk"[tiab] OR "health risks"[tiab] OR "preventive health"[tiab] OR prevention[tiab] OR "disease prevention"[tiab] OR "health intervention"[tiab] OR "health interventions"[tiab] OR "lifestyle intervention"[tiab] OR "lifestyle interventions"[tiab] OR "behavioral intervention"[tiab] OR "behavioural intervention"[tiab] OR "physical activity intervention"[tiab] OR "physical activity interventions"[tiab] OR "physical activity promotion"[tiab] OR "exercise intervention"[tiab] OR "exercise interventions"[tiab] OR "exercise promotion"[tiab] OR cardiovascular[tiab] OR "cardiovascular health"[tiab] OR "cardiovascular disease"[tiab] OR "cardiovascular diseases"[tiab] OR diabetes[tiab] OR "metabolic syndrome"[tiab] OR "metabolic health"[tiab] OR "chronic disease"[tiab] OR "chronic diseases"[tiab] OR "noncommunicable disease"[tiab] OR "noncommunicable diseases"[tiab] OR "non-communicable disease"[tiab] OR "non-communicable diseases"[tiab] OR "societal impact"[tiab] OR "societal impacts"[tiab] OR "social impact"[tiab] OR "social impacts"[tiab] OR "societal benefit"[tiab] OR "societal benefits"[tiab] OR "societal change"[tiab] OR "social change"[tiab] OR "behavioral change"[tiab] OR "behavioural change"[tiab] OR "behavior change"[tiab] OR "behaviour change"[tiab] OR "lifestyle change"[tiab] OR "lifestyle changes"[tiab] OR "lifestyle modification"[tiab] OR "lifestyle modifications"[tiab] OR safety[tiab] OR "traffic accident"[tiab] OR "traffic accidents"[tiab] OR "pedestrian safety"[tiab] OR "road safety"[tiab] OR injury[tiab] OR injuries[tiab] OR "risk taking"[tiab] OR "risky behavior"[tiab] OR "risky behaviour"[tiab] OR "distracted walking"[tiab] OR "distracted driving"[tiab] OR privacy[tiab] OR "data privacy"[tiab] OR "data security"[tiab] OR "personal data"[tiab] OR "data collection"[tiab] OR surveillance[tiab] OR crime[tiab] OR trespass*[tiab] OR violence[tiab] OR "social problem"[tiab] OR "social problems"[tiab] OR "social behavior"[tiab] OR "social behaviour"[tiab] OR "cultural impact"[tiab] OR "cultural impacts"[tiab] OR "cultural phenomenon"[tiab] OR "cultural phenomena"[tiab] OR "social phenomenon"[tiab] OR "social phenomena"[tiab] OR "public space"[tiab] OR "public spaces"[tiab] OR "urban space"[tiab] OR "urban spaces"[tiab] OR "place making"[tiab] OR placemaking[tiab]))  Limits: 2016-2025; English; title/abstract |
