# Supplementary material for: Motivational Factors, Physical Activity Impacts, and Sociopsychological Effects of Pokémon GO in Players Over the Years: Scoping Review
Source: J Med Internet Res. 2026 Jul 31;28:e89235. doi: 10.2196/89235 (PMC13430953; doi:10.2196/89235)
Supplement: Multimedia Appendix 2 [file jmir-v28-e89235-s002.docx]

| **Author** | **Year** | **Country** | **Study design/ Article type** | **Data collection period (if stated)** | **Study population** | **Intervention type** | **Assessment methods** | **Outcome_types** | **Key findings and overall conclusion** | **Main category of the findings in relation to research questions** | **Type of measure for PA** |
| --- | --- | --- | --- | --- | --- | --- | --- | --- | --- | --- | --- |
| Alha et al. [1] | 2019 | Finland | Quantitative survey | September 1, 2016 at 7:00 p.m. until September 7th, 2016 12:00 p.m. | Finnish Pokémon GO (PoGo) players | Not applicable | Open-ended and close-ended survey questions | Reasons to start, continue, and quit playing PoGo | Earlier experiences especially with the same franchise, social influence, and popularity were the most common reasons to adopt the game, while progressing in the game was the most frequently reported reason to continue playing. The player's personal situation outside the game and playability problems were the most significant reasons to quit the game.  Progression, situation, positive aspects, mechanics, interest and expectations were positively and statistically significantly associated with playing frequency, whereas technology was negatively associated, indicating that the novelty of the technology might wear off quickly.  The player's situation, various problems, shortcomings, poor game mechanics, slow or difficult progression, the nature of the game, changes, the company behind the game, and social influence were mentioned as reasons for quitting the game. | Reasons to adopt, continue, or quit playing PoGo | Not applicable |
| Alloway & Carpenter [2] | 2021 | United States | Longitudinal analysis; randomized controlled trial | 2019 | Students from a public Florida university | Pokémon Go vs. Walking without phone | Cognitive, affect, and prosocial tasks and surveys Working memory was measured using both verbal and visuospatial tasks Computerized assessment of auditory sustained attention 20-item Positive and Negative Affect Schedule Interpersonal Reactivity Index was used to measure empathy | Working memory, affect, empathy, attention | Although Pokémon Go was not designed to directly improve cognition, game play does seem to improve some aspects of WM, attention, and affect.  The current study specifically found an improvement in negative affect. Pokémon Go may better assist those who are feeling down and be more effective in aiding those with more extreme forms of negative affect such as depression lending its use as a possible cost-effective therapeutic tool. | Cognitive and affective benefits of PoGo | Not applicable |
| Alomar et al. [3] | 2019 | Not mentioned | Quantitative survey | July to September 2017 | Crowd workers who are located in the United States | Not applicable | 6 independent scenarios designed for each study survey was posted twice online; one time without explicitly mentioning the gaming context and another one that clearly envisions a participant as a Pokémon Go player | Whether Pokémon Go has contributed to forming motives for trespassing into private properties, violating respected regulations, compromising personal physical safety, violating street-crossing rules and parking cars in illegal spots. | Overall, the long-term negative impact that Pokémon Go players have exhibited in our studies is minimal, and could have been caused by other influential factors, such as players' contextual and personal attributes. | Risky behaviours of PoGo players | Not applicable |
| Althoff et al. [4] | 2016 | United States | Quasi-experimental design | July 6, 2016 (US release date of Pokémon Go) to August 23, 2016 | US users of Microsoft products who have agreed to link data from their Microsoft Band wearables and their online activities to understand product usage and improve Microsoft products. | Not applicable | Compare changes in physical activity for Pokémon Go users with changes for a large control group of US wearable users and further compare the effect of Pokémon Go with 4 leading mobile health apps | Effect of Pokémon Go on physical activity | Pokémon Go leads to significant increases in physical activity over a period of 30 days, with particularly engaged users increasing their activity by 1473 steps a day on average, a more than 25% increase compared with their prior activity level (P<.001).   In the short time span of the study, Pokémon Go has added a total of 144 billion steps to US physical activity. Furthermore, Pokémon Go has been able to increase physical activity across men and women of all ages, weight status, and prior activity levels showing this form of game leads to increases in physical activity with significant implications for public health. In particular, Pokémon Go is able to reach low activity populations. | Physical activity impacts of PoGo | Wearable/Phone |
| Arjoranta et al. [5] | 2020 | International | Qualitative survey | Mid-July to mid-November 2016 | International, English speaking Pokémon GO players | Not applicable | Critical incident technique (CIT), which focuses on acquiring individuals’ descriptions of their actual behavior | Catching new Pokémon, Visiting Pokéstops, Exploring Pokéstops, Hatching eggs, Fighting in gyms, Finding specific Pokémon, Using items, Pokémon theme, Game location tied to physical location; how these features tie to specific behavior change | On a general level, there were 2 game features behind all behavior changes: game location tied to physical location and catching new Pokémon. Thus, these are the 2 most central features that supported the respondents’ (intentional or unintentional) behavior changes.  Surveyed players changed their behaviors while or after playing Pokémon GO. The respondents reported being more social, expressed more positive emotions, found more meaningfulness in their routines, and had increased motivation to explore their surroundings. | Successful PoGo game features (motivation, physical and mental health, social well-being) | Not applicable |
| Ashar et al. [6] | 2019 | India | Longitudinal analysis | 2018 | MBBS students | Not applicable | Depression, anxiety, and stress scale | Depression symptoms | The major finding of the present study is that regular players of Pokémon Go had significantly higher levels of depression, anxiety, and stress as compared to nonplayers. Further analysis revealed that 66% of the players had moderate or higher anxiety compared to 12% of the nonplayers. More importantly, 38% of the players had moderate to severe depression compared to none of the nonplayers.  Regular playing of Pokémon Go results in adverse consequences including exposure to dangerous situations, stress, anxiety, and depression. | Negative mental health correlates among PoGo players | Not applicable |
| Ayers et al. [7] | 2016 | International | Google search and Twitter analytics | July 10 through 20, 2016 | 345,433 Twitter posts | Not applicable | A random sample of 4000 tweets was generated; Google News reports that included “Pokémon” and “driving” were obtained | Whether (1) a driver was playing, (2) a passenger was playing, or (3) a pedestrian interacted with traffic while playing Pokémon GO. | Thirty-three percent (95% CI, 31%-34%) of tweets indicated that a driver, passenger, or pedestrian was distracted by Pokémon GO. Eighteen percent (95% CI, 17%-19%) of tweets indicated a person was playing and driving.  There were 14 unique crashes—1 player drove his car into a tree—attributed to Pokémon GO in news reports during the same period. | Risky behaviours of PoGo players | Not applicable |
| Barbero et al. [8] | 2018 | United States | Case reports review | July 5, 2016 to November 5, 2016 | All text physician notes containing the word "Pokemon" or "Pokémon" | Not applicable | Review of physician's notes | Benefits, adverse events, and incedental mention of Pokémon Go | Injuries were similar to those seen with other light-to-moderate outdoor physical activities. The number of severe injuries reported was low; this suggests that the game is safe to play. Pokémon Go may be reaching a population that requires increased physical activity. | Risk and physical activity impacts of PoGo | Not applicable |
| Barbieri et al. [9] | 2017 | Italy | Case report |  | Pokémon Go player | Not applicable | The patient’s history, the circumstances in which the collision happened, imaging data, and clinical course information were recorded | Not applicable | 25-year-old male who suddenly crossed a road while playing Pokémon Go and was hit by a van, reporting several injuries and being assisted by the Emergency Medical Service of the hospital.  Cases like the one presented here point out that these games could pose a significant risk to users who play while walking, cycling, or driving in unsafe areas such as city streets, because players become distracted and may ignore surrounding hazards. | Risky behaviours of PoGo players | Not applicable |
| Bazina et al. [10] | 2025 | Croatia | Conference paper, quantitative survey | 2021 onwards | Croatian Pokémon GO players | Not applicable | Online anonymous survey consisting of 39 questions. The first three questions focused on demographic data, subsequent 14 questions addressed players’ gaming habits, and 22 statements related to the game's impact on their physical activity, sociability, and overall well-being. | Daily playtime, time spent moving during PoGo playing time, average kilometers walked per week, sociability, well-being, and motivation of players to visit new places | Average player spends 342 minutes per week moving solely to play Pokémon GO, more than double the World Health Organization's recommended minimum of 150 minutes of weekly physical activity. 90% of respondents who identified as hardcore players met the minimum physical activity criteria, as did 78.6% of those identifying as a mix between casual and hardcore players.  Additionally, 75% of players reported walking more frequently, 58.3% made new friends, and 55.6% experienced improved mental health, affirming the game’s multifaceted positive effects. | Well-being and physical activity impacts of playing PoGo | Not applicable |
| Beach et al. [11] | 2021 | United States | Observational |  | Experienced (level 5 and above) Pokémon Go (PoGo) players (18 years of age and above) participated in this study | PoGo vs traditional walking | Direct observation documentation and time-stamped accelerometer data, for the 1.77 km walking loop | Total steps, aerobic steps, caloric expenditure, distance, amount of time in sedentary and moderate intensity, and number of stops | Continuous PoGo produced similar PA to traditional walking, while intermittent PoGo reduced PA, nevertheless PoGo may be a strategy for increasing PA participation.  A larger sample size and monitoring players during free-living play would provide greater insight into the prevalence of the two PoGo playing styles identified in this study. | Physical activity impacts of PoGo | Accelerometer |
| Beach et al. [12] | 2019 | United States | Observational | June to August 2016 | Users of the Maryville–Alcoa greenway | Not applicable | Omron HJ-322U (Bannockburn, IL) pedometer and ActiGraph GT3X (Pensacola, FL) accelerometer | Steps tracked and physical activity (PA) intensity | While Pokémon Go may encourage younger individuals to engage in PA, Pokémon Go users appear to be spending only 55% of their 42-minute walk in health-enhancing MVPA. This may be due to the fact that despite spending the same amount of time on the greenway as others, Pokémon Go users are likely stopping more, and when walking, walking at a slower pace due to their focus on playing Pokémon Go. However, Pokémon Go’s engagement of younger individuals could serve as an effective gateway into an active lifestyle. | Physical activity impacts of PoGo | Pedometer and accelerometer |
| Biel [13] | 2016 | United States | Mixed methods analysis (Thesis) |  | 18 and older who had either actively used Pokémon GO in the past month, or within the semi-structured interview portion only, have knowledge of Pokémon GO but have not yet played | Not applicable | Uploading steps data from HealthKit via Open Humans website, www.openhumans.org  Ecological momentary assessment (EMA) application called personal analytics companion – PACO  Semi-structured interviews | Step count, physical activity  Motives for playing, game mechanic preferences, modes and locations of play, and prior physical activity habits | Small but non-significant trend towards increased steps taken on days when a participant played  Nostalgia was a strong motivator for many to play the game, which was counter to theoretical expectations. In line with previous theory, results suggested that operant conditioning principles appeared to be at work in terms of fostering game play use. | Physical activity impacts of PoGo and motivation to play | Wearable/Phone |
| Bonus et al. [14] | 2018 | United States | Quantitative survey | Three weeks after the release of Pokémon Go in the United States | Pokémon Go players and people high in social anxiety | Not applicable | Differential Emotions Scale, Time Perspective Inventory, Exercise, Friendship initiation and intensification, Brief Resilience Scale, Satisfaction with Life Scale, loneliness scale, Patient Health Questionnaire-4 scale | Positive and negative affect, nostalgia, exercise, friendship initiation and intensification, media effects, life satisfaction, resilience | Pokémon Go was associated with various positive responses (increased positive affect, nostalgic reverie, friendship formation, friendship intensification, and walking), most of which predicted enhanced well-being. For highly anxious participants, gameplay showed weaker associations with positive affect (a predictor of enhanced well-being), but also weaker associations with nostalgic regret (a predictor of reduced well-being).  Results hint at a moment in time where simply playing a video game might have made people happier, encouraged nostalgic reverie, created or deepened friendships, and motivated players to walk around their neighborhoods; these experiences might also have enhanced players’ well-being. | Well-being impacts and motivation to play PoGo | Not applicable |
| Brecht [15] | 2020 | United States | Longitudinal analysis; randomized controlled trial |  | Players with mild levels of depressive symptoms | Pokémon Go vs Candy Crush | Social Connectedness Scale-Revised, Patient Health Questionnaire - 8, Godin-Shephard Leisure-Time Physical Activity Questionnaire | Depressive symptoms, physical activity, and social connectedness | Participants in both groups experienced a moderate decrease in depressive symptoms and an increase in social connectedness, but no change in physical activity. Differences between the two groups were not found to be statistically significant across the three outcome variables.  Based on these findings it does not appear that Pokémon Go was more effective at treating depressive symptoms, increasing physical activity, or increasing social connectedness when compared to a traditional smartphone game. | Mental and physical health impacts of playing PoGo | Self-report questionnaire |
| Broom & Flint [16] | 2018 | United Kingdom | Quantitative survey; longitudinal analysis | During a four-week period after Pokémon Go was released in the UK. | Pokémon Go users and non-users | Not applicable | Physical Activity and Pokémon Go questionnaire | Physical activity, sitting time and perceptions of the physical activity and health benefits | Pokémon Go use can increase the frequency of days of physical activity benefitting health. Users at both time points maintained their physical activity behaviour but increased sitting time on weekdays, highlighting that another intervention to prevent sitting is needed. | Physical activity impacts of PoGo | Self-report questionnaire |
| Broom et al. [17] | 2019 | United Kingdom | Quantitative and qualitative survey; longitudinal analysis | Soon after launch in the United Kingdom | Pokémon Go users and non-users | Not applicable | Physical Activity and Pokémon Go questionnaire | Motivations for using the Pokémon Go App  Reasons for using or not using the Pokémon Go App in the future | Future smartphone applications aiming to increase physical activity ensure that the objectives evolve to maintain initial interest and motivation to engage with an application  Consider the required phone storage and capability as this might present a barrier to continued use or for some an inability to download the application in the first instance  Consider potential concerns of using the application including the safety of users and those around them | Risk and physical activity impacts of PoGo | Self-report questionnaire |
| Broom et al. [18] | 2018 | United Kingdom | Quantitative and qualitative survey; longitudinal analysis | Soon after launch in the United Kingdom | Pokémon Go users and non-users | Not applicable | Physical Activity and Pokémon Go questionnaire | Physical activity, sitting time and perceptions of physical activity and health | Users’ reported that they undertook less days of vigorous physical activity than non-users’ but more days of moderate physical activity and walking.  Users’ at both time points maintained their physical activity behaviour but increased sitting time on weekdays | Physical activity impact of PoGo | Not applicable |
| Bueno et al. [19] | 2020 | International | Quantitative survey | December 2018 to June 2019 | Pokémon Go players | Not applicable | Survey instrument based on the theoretical background regarding Uses & Gratification theory and online games | Hedonic gratification: enjoyment (ENJ), fantasy (FAN) and escapism (ESC).   Social gratification: social interaction (SINT) and social presence (SPRE)   Utilitarian gratification: achievement (ACH) and self-presentation (SELFP)   All these constructs in turn influence the continuance intention (CI) to use the Pokémon Go game | The dataset indicates that the proposed model offers an appropriate fit for the defined connections, as the positive influence of enjoyment, escapism, social interaction, social presence and achievement on the CI of playing Pokémon Go are clearly confirmed.  It can be concluded that users achieve gratification when they use augmented reality mobile games | Successful PoGo game features (motivation) | Not applicable |
| Burney et al. [20] | 2017 | Not mentioned | Quantitative survey |  | Pokémon Go users | Not applicable | Questionnaires based on four-factor learnability, efficiency, effectiveness and memorability | Perceived ease of use: learnability and memorability  Perceived usefulness: effectiveness and efficiency | Albeit all connection coefficients are not huge, the importance of their relationship should be additionally explored  Perceived ease of use is in accordance with core elements of usability "Memorability" and "Learnability", and it's causality is distingushed from perceived usability. Pokémon Go being easy to learn and play led to its continued use | Successful PoGo game features (motivation) | Not applicable |
| Butcher et al. [21] | 2021 | Australia | Google search and Twitter analytics | Prior to its launch (Jan ’16–June ’16), during its peak (July ’16–December ’16), and into its decline (Jan ’17–June ’17) | Australian Google Search (AGS) data | Not applicable | Google search trends for pervasive gaming for Pokémon Go and application of path to discontinuance for pervasive mobile games | Google search trends results and twitter posts and comments | The development of the P2D_PMG model provides a new conceptual framework to illustrate the distinct forms discontinuance manifests in, for the first time. Scholarly rigour of the P2D_PMGs is achieved through validating and extending Soliman and Rinta-Kahila’s (2020) framework for ‘discontinuance’ through its five forms. These forms are revealed as access and on-boarding (rejection), disconfirmation and hedonic adaptation (regressive discontinuance), technological, social, third parties, and personal issues (quitting), re-occurrences of hedonic adaptation (temporary), and alternatives and iterations (replacement). | Understanding continuing/discontinuing PoGo | Not applicable |
| Caci et al. [22] | 2019 | Italy | Quantitative survey |  | Pokémon GO players | Not applicable | Pokemon GO Motivational Scale (PokeGOMS) Italian version of TIPI | Motivational needs and personality types for Pokemon GO adoption | Results at EFA and CFA showed three dimensions for primary motives for using Pokémon GO: Personal Needs, Social Needs, and Recreation. Specifically, Personal Needs include expressing aggression, sexuality, and hidden aspects of the gamer personality. Social Needs involve managing a variety of social experiences, knowing new people and cultural worlds different from their own or expressing creativity. Recreational needs are related to spending free time, performing physical activities, and meeting friends.  More introverted gamers driven by their recreational needs tend to spend more time using Pokémon GO. Less agreeable people driven by personal needs tend not only to spend time on playing Pokémon GO but also to have more game sessions on a day. People who tend to be competitive and not compassionate with others find the Pokémon GO a breeding ground to express their personality traits using the virtual environment of the game. Low conscientiousness people, who usually tend to neglect daily activities related to work or family, play Pokémon GO capturing a high number of Pokemon creatures, but they are driven explicitly by their social needs. | Personality traits and motives of PoGo players | Not applicable |
| Chen & Pai [23] | 2018b | Taiwan | Observational | April 2017∼March 2018 | Pedestrians | Not applicable | Pedestrian's risk-taking inclinations observed using two video cameras (D-Link DCS-2630 L Full HD 180-Degree Wi-Fi Camera) located at both sides of a signalised street in Taipei City, Taiwan.  Interview after they had completed crossing the street | Smartphone game types, data use limit, social conformity, participant's risk-taking inclinations | Racing games appears not to be associated with pedestrian's risk-taking behaviours as much as Pokemon Go does. Instead, compared to other smartphone games, Pokemon Go is most associated with pedestrian's risk-taking inclinations. | Risky behaviours of PoGo players | Not applicable |
| Chen & Pai [24] | 2018a | Taiwan | Observational | from 6/8/2016 until 5/9/2016 | Distracted and undistracted pedestrians (the control group) | Not applicable | Video cameras for observing pedestrians’ road-crossing behaviors at an uncontrolled intersection  Data such as phone features, distraction types, and personal attributes of the pedestrians were obtained in the interviews conducted after pedestrians had completed crossing the street. | Street-crossing behaviors such as the average crossing times, sudden movement, head-turning frequency, not looking at traffic, looking at the wrong side of traffic comapred to secondary tasks | The results indicate that the unsafe crossing behaviors (e.g., sudden movement, fewer head-turning frequencies, not looking at traffic before crossing, not looking at left side of traffic first) were more prevalent among those gaming with “Pokemon Go.”  Logistic regression models reveal several important correlates of unsafe crossing behaviors: being a student, large phone screen (5 in. or larger), and having un-restricted 4G Internet data allowance. | Risky behaviours of PoGo players | Not applicable |
| Chen et al. [25] | 2018 | Taiwan | Quantitative survey | August 6th, 2016 to August 17th, 2016 | Pokémon Go players in Taiwan | Not applicable | Survey on a five-point Likert-type scale | Network externalities, intrinsic motivations, gratifications, and flow. | The results reveal that gratification plays a more important role than flow in retaining users. Furthermore, over time the impact of network externalities and intrinsic motivation gradually disappeared, leaving only a weak serendipity-gratification-stickiness relationship. These findings might explain the game's drop off in popularity. | Understanding continuing/discontinuing PoGo | Not applicable |
| Chen et al. [26] | 2018 | Taiwan | Observational | 6 August to 5 September 2016 | Video cameras were set up to observe pedestrians’ road-crossing behaviours at a selected location. All pedestrians that were observed to be distracted by their phones were participants in the study. | Not applicable | Video cameras were used to capture pedestrians’ street-crossing behaviours | Crossing time, sudden movements, running a red light, and walking outside the crosswalk | Results indicate that unsafe crossing behaviours were more prevalent among those playing ‘‘Pokemon Go”. Texting via instant-message apps appeared to be the second-most risk distracting activity.  Results of the logistic models reveal that contributing factors to unsafe behaviours include being a student, phone screen of 5 in. or larger, and having an unrestricted 4G Internet data allowance. Two interaction terms (gaming × students, and gaming × unlimited 4G data allowance) in the models appear to be important determinants of unsafe crossing behaviours. | Risky behaviours of PoGo players | Not applicable |
| Cheng [27] | 2019 | Taiwan | Quantitative survey | March 5th to April 13th, 2018 | Students from each grade in 3 Taiwan middle schools | Not applicable | Parenting Questionnaire, temperament scale, Motivation for Playing Pokemon Go Questionnaire, IGD-20, Well-being Questionnaire, | Parenting, temperament, motivation for playing Pokemon Go, gaming addiction, well-being | The research finding revealed a positive effect of demanding parenting on temperament, motivation for playing Pokémon Go, and IGD, reaffirming the crucial role of parenting.  Interior temperament can have a positive effect on motivation for playing Pokémon Go and well–being.  Motivation for playing Pokémon Go does show a positive effect on IGD. In particular, the dimension of form, story and personal interaction do contribute significant effect on IGD. Motivation for playing Pokémon Go can serve as the mediator for a positive relationship between parenting and IGD. | Factors affecting PoGo motivation and effect on problematic gaming | Not applicable |
| Cheng et al. [28] | 2022 | 166 regions across 12 English-speaking countries | Observational | January 1, 2016, and December 12, 2016 | Google users in 12 English-speaking countries | Not applicable | Ananlysis of internet search trends before and after release of Pokémon Go  Capture local trends in depression using Google Trends data of the Google Misery Index | Depression-related search frequency internationally | Empirically documented a disproportionate decrease in depression-related search in those regions where users are able to play Pokémon Go.   This finding lends credence to anecdotal claims that location-based mobile games may alleviate symptoms of depression of their users, underscoring the mental health opportunities of location-based mobile gaming and creating new opportunities for information systems research. | Mental health impacts of playing PoGo | Not applicable |
| Clark [29] | 2018 | Not mentioned | News article |  |  | Not applicable | Not applicable | Not applicable | Nearly 90% of Pokémon Go players meet new people while playing, fostering community connections across generations. Despite these social benefits, 31% of users have felt unsafe, highlighting risks related to real-world dangers and data privacy. | Risk and social impacts of PoGo | Not applicable |
| Coldewey [30] | 2016 | United States | News article |  |  | Not applicable | Not applicable | Not applicable | In 2016, New York State Department of Corrections and Community Supervision has imposed a new condition of parole for sex offenders under community supervision; the restriction will apply to some 3,000 sex offenders; the letter also adds Niantic to the list of companies regularly updated on those offenders’ whereabouts and online handles. | Risk of playing PoGo | Not applicable |
| Conditt [31] | 2016 | United States | News article |  |  | Not applicable | Not applicable | Not applicable | Pokemon Go isn't presented as a tool to help treat anxiety or depression; instead, it's a game that happens to cultivate healthy behaviors. But for all of its achievements, Pokemon Go has limits. It may be a wonderful motivator for players to leave their houses, get some light exercise and meet new people, but it isn't a substitute for professional treatment. | Mental and physical health impacts of playing PoGo | Not applicable |
| Costello and Smith [32] | 2022 | Not mentioned | Quantitative and qualitative survey | December 2018 to October 2019 | Candidates from industry and academia | Not applicable | Qualitative and quantitative questionnaire | Sensitivity of forming a community, buildign freindships, isolation and anxiety, depression, health and well-being | Industry and academia need to find suitable ways to assist individuals dealing with well-being, anxiety, and depression. Even though some of the results are not entirely conclusive, the authors have demonstrated that only 55% of participants agreed with Mobile Games assisting with depression or that 52% agreed that it keeps the participants active. There were parts where Mobile Games has demonstrated that it has helped with forming friends, dealing with anxieties, and creating communities. | Mental health impacts of playing PoGo | Not applicable |
| de Souza e Silva et al. [33] | 2023 | Brazil and Kenya | Interview | June to August 2019 | Pokémon Go players in Rio de Janeiro (Brazil) and 13 in Nairobi (Kenya) | Not applicable | Individual and semi-structured interviews, with the intent to build rapport with participants | (1) how gameplay (i.e. the physical mobility and sociability required  to play the game) impacts players’ mobilities in these cities, and  (2) how systemic urban issues (such as the risk associated with physical mobility, socioeconomic inequalities, and gender and racial inequalities) in these densely populated environments shape the way people play | Playing Pokémon Go in these cities includes a great deal of managing everyday life risks, which include concerns for theft, physical and sexual violence, and racialized and class-based discrimination. These risks do not prevent people from playing, but they do influence how people move through urban spaces, their choice of specific times and spaces to play, how they deal with gender and racial inequalities, and their way of socializing in the game and through social media platforms.   These findings are relevant for understanding the social and political aspects of networked urban spaces as well as for investigating games as venues through which we can understand ordinary life, racial, gender, and socioeconomic inequalities. | Risk of playing PoGo | Not applicable |
| Dunham et al. [34] | 2025 | International | Quantitative survey | July 2019 for 10 days | 347 hardcore players (32.4%), 724 casual (67.6%) | Not applicable | Questionnaire about demographics, traits, and gratifications | To identify where on the hardcore/casual spectrum LBG players lie and the traits and gratifications of those players | Pokémon GO participants expressed affinity for each trait; although casual participants align more with aesthetics orientation, hardcore players gravitate towards the goal, action, and social orientation traits. There is no significant difference between the casual and hardcore players for immersion orientation trait.  In term of gratifications, participants expressed nostalgia for the franchise. Hardcore participants expressed significantly more positive gratification for enjoyment and competition, while casual participants scored nearly a point higher than their hardcore counterparts for socialisation. Challenge for hardcore participants is neutrally received; however, casual participants express negative gratification. Trends concerning Pokémon GO did not gratify participants. | Personality traits and gratifications of PoGo players | Not applicable |
| Ellis et al. [35] | 2020 | International | Observational |  | Users on four subreddits dedicated to Pokémon GO or Harry Potter: Wizards Unite (r/WizardsUnite, r/PokemonGO, r/harrypotterwu, and r/TheSilphRoad) | Not applicable | The survey included a total of 40 questions, both quantitative and qualitative. | Responses from quantitative questions relating to video game use, exercise, and mental well-being are reported here, as well as responses from two qualitative questions regarding motivation to play and the impact of games on mental health. | AR games have the potential to promote physical and mental health during the COVID-19 pandemic. Used by populations under isolation and distress, these games can improve physical and mental health by providing virtual socialization, sustained exercise, temporal routine, and mental structure. | Mental and physical health impacts of playing PoGo | Self-report questionnaire |
| Escaravajal-Rodríguez [36] | 2018 | Spain | Quantitative survey | 2016 | Spanish Facebook users | Not applicable | Ad-hoc opinion questionnaire was designed with closed-ended questions  Simultaneous Multi-Attribute Trade Off (SIMALTO) response choices | Physical activity, social relations, spatial orientation and places | The results show that there was an increase in physical activity; users of Pokémon GO play with fellow players and meet new people, plus they also get to know new streets and points of interest in their city; and finally, there has been an increase in their outings into nature. | Physical activity impacts and social benefits of PoGo | Self-report questionnaire |
| Etherington [37] | 2016 | Not mentioned | News article |  |  | Not applicable | Not applicable | Not applicable | PokeFit, from P3 communications, helps trainers get a real-time grasp on the positive health benefits they’re getting by playing Pokemon Go. The app itself keeps a log of your sessions, giving you a breakdown of how long each lasted and how much distance you traversed during your trip. When you’re using Pokemon Go, it displays a small rectangular frame in the upper left corner of your screen, providing quick access to info at a glance. | Health features of PoGo | Not applicable |
| Evans & Saker [38] | 2019 | International | Quantitative and qualitative survey | May 2017 and July 2017 | Users of Pokémon Go | Not applicable | Mixture of closed questions and open questions | Space and place; play; sociability; identity | First, players will often use new routes and break established, habitual routes of movement to improve their performance in the game and to play the game to a higher level. Second, thanks in part to this need to change established wayfinding, players are more likely to encounter new places in their everyday playing of the game, and are in some cases more inclined to visit new places or go to places they normally would have no need or no desire to visit because of their playing. Third, the playing of Pokémon Go has subsidiary social benefits that go beyond changes in mobility but are the result of this modification. | Social benefits of PoGo (successful features) | Not applicable |
| Evans et al. [39] | 2021 | Not mentioned | Quantitative survey |  | Current and former Pokémon Go players using Amazon’s Mechanical Turk (mturk) | Not applicable | Pokémon Go Motive Scale | Motives for playing Pokémon Go, average play time, social or solo play | Players motivated by initiating relationships play the game more, find it more challenging, and find it more interesting than they did in the past. Players motivated by maintaining current friendships play the game less, find it less challenging, and find it less interesting than previously. Players motivated by fun find the game more challenging. Finally, those motivated by exercise report increased solo play time, and more recent social play time.  Intrinsic rewards hold the most staying power as a distinct motivation for gameplay. The primary motivation for continued gameplay over time is relationship initiation. | Motivation to play PoGo | Not applicable |
| Ewell et al. [40] | 2020 | United States | Diary study |  | Pokémon Go players | Not applicable | Participant estimated the total number of minutes engaged in the respective activity, estimated number of minutes the participant spent interacting, Satisfaction with Life Scale, Subjective Vitality Scale | Time spent playing the game and amount of exercise, interactions with friends and strangers, life satisfaction, vitality | Daily time spent playing Pokémon Go was related to higher scores of life satisfaction, vitality, and greater social interactions and conversation with both friends and strangers, but not with increased daily exercise. Increased total gameplay across the week was associated with increased interaction and conversations along with more exercise. | Well-being and physical activity impacts of playing PoGo | Self-report questionnaire |
| Faccio & McConnell [41] | 2020 | United States | Case reports review | March 1, 2015, through November 30, 2016 | Police Accident Reports Data | Not applicable | OLS panel regressions | Whether the number of vehicular crashes increased disproportionately in the vicinity of PokéStops after the introduction of Pokémon GO | Users playing the augmented reality game Pokémon GO while driving gave rise to a disproportionate increase in vehicular crashes, injuries, and fatalities in the vicinity of PokéStops following the introduction of the game.  Focusing only on costs of vehicular damage from police reports, the lower bound estimate of the incremental costs is $334,145. Incorporating estimates of the losses associated with personal injuries from the Insurance Information Institute and the CDC gives a lower bound estimate of the incremental costs associated with crashes of $1,226,300. Even ignoring the value of lives lost, these costs are significant, giving rise to an implied increase in vehicular insurance premiums of 2.47 percent. | Risk and societal impact of PoGo | Not applicable |
| Finco et al. [42] | 2017 | Brazil | Qualitative survey | 5 to 19 January 2017 | Pokémon Go players | Not applicable | Qualitative questionnaire | Questions on personal data (age, sex, educational background and profession), about Pokémon GO and its playability | Users have changed physical activity habits such as going more often to practice sports and exercises with friends and family outside of the game. Many players mentioned that meeting new users was a good way to socialise through making groups to walk or run together, as an extra motivation.  Pokémon GO is one of the first mobile-based gameplay that can be used to promote a healthier lifestyle with a new way of interaction, changing sedentary lifestyles with a big potential to be used in Health Education. | Physical activity impacts and social benefits of PoGo | Self-report questionnaire |
| Fisher-Reid [43] | 2020 | United States | Interview | September 2018 to May 2020 and all Community Days were attended except for January and October 2019. | Mostly undergraduates from Stony Brook University | Not applicable | Analysis of interview transcripts | Recurrent themes mentioned by the participants in interview transcripts | Some participants attended for self-interest such as gaining strong Pokémon and completing their collection. Others attended for socialization and community building such as having fun meeting new people and feeling a sense of “community.” A couple people participated for both tangible and intangible benefits. While the self-interest aspects of obtaining incentives did play a part in Community Days, players also gathered and shared a sense of camaraderie on Community Day. | Social benefits of PoGo | Not applicable |
| Fountaine et al. [44] | 2018 | United States | Observational |  | Recreationally active college students | Not applicable | Participants were fitted with an accelerometer, pedometer, and heart rate monitor to assess the activity demands  Physical Activity Readiness Questionnaire | Intensity of physical activity in the 60-minute playtime | Of the 60-min allotted playtime, accelerometry indicated 82% was achieved via moderate-to vigorous-intensity physical activity. Pedometer counts indicated approximately 6000-steps or 100-steps/min, indicative of moderate-intensity physical activity. Heart rates were approximately 50% of age-predicted maximum, also indicative of moderate-intensity physical activity. The results of this study provide evidence that playing one hour of Pokémon GO can be an effective means of accumulating recommended levels of daily/weekly physical activity. | Physical activity impacts of PoGo | Pedometer, accelerometer, and heart rate monitor |
| Gabbiadini et al. [45] | 2018 | United States | Quantitative survey |  | Participants from Amazon Mechanical Turk | Not applicable | Attitude Regarding Physical Activities for Health and Fitness Scale | Recency and frequency of their physical activity, frequency of Pokémon Go app usage, extent to which people share their achievements on social media, amount of specific physical activity related to the use of Pokémon Go | Pokémon Go related physical activity significantly reversed the positive effects of the app on participants' overall physically active behavior, suggesting that the mere adoption of the app does not reliably change people's behavior in general. The increase in physical activity levels is rather explained by the exercise required by the game. | Physical activity impacts of PoGo | Self-report questionnaire |
| Gee et al. [46] | 2021 | Malaysia | Quantitative survey | 3 years after the game was released | Pokémon Go players | Not applicable | Global Physical Activity Questionnaire | Time spent playing Pokémon Go, level of physical activity | The results of the current study showed no significant association between days spent playing Pokémon Go and level of physical activity (p = .14), hours spent playing Pokémon Go and physical activity (p = .516), or between daily hours spent playing Pokémon Go and daily sedentary time (p = .283).   Nevertheless, the mean of the study reported that the physical activity level of the players increased concurrently as the player’s game frequency increases. | Physical activity impacts of PoGo | Self-report questionnaire |
| Ghazali et al. [47] | 2019b | Malaysia | Quantitative survey | 2017 | Current players of Pokémon Go (PoGo) in Malaysia | Not applicable | The questionnaire was developed by adapting scales from existing literature to suit the current research context. | Achievement, Challenge, Escapism, Social interaction, Need-to-collect, Nostalgia, Network externality, Enjoyment, Flow, Community involvement, Continuance intention, Purchase intention | Enjoyment, network externalities, community involvement and the need-to-collect significantly influence players’ continuance intention. Furthermore, the findings reveal that flow and nostalgia have indirect effects on players’ continuance intention, which in turn significantly influences their purchase intention. | Understanding continuing/discontinuing PoGo (motivation) | Not applicable |
| Ghazali et al. [48] | 2019a | Malaysia | Quantitative survey |  | Current players of Pokémon Go (PoGo) in Malaysia | Not applicable | The instrument consisted of three sections: gaming experience, motivations and behavioural intentions for the game, and demographic information | Achievement, Challenge, Escapism, Social interaction, Need-to-collect, Nostalgia, Network externality, Enjoyment, Flow, Online community involvement, Continuance intention to play PoGo (ContInt) | Enjoyment is the most important mediator, mediating three U&G predictor constructs (achievement, escapism, challenge and social interaction) and the outcome ContInt. Flow did not have any influence on ContInt unless coupled with enjoyment as a serial mediator. Network externality and nostalgia were found to only influence ContInt through mediators, online community involvement and need-to-collect Pokémon Monsters, respectively. Overall, the results show evidence of four indirect-only mediation paths and one complementary partial mediation path. | Factors affecting PoGo motivation | Not applicable |
| Giller et al. [49] | 2025 | Poland | Quantitative survey | 2025 | Adult Pokémon GO players | Not applicable | Validated Polish version of the International Physical Activity Questionnaire (IPAQ long form), a demographic questionnaire, and a series of questions regarding sports participation and engagement with Pokémon GO | Physical activity levels, involvement in sports, participants’ experiences with Pokémon GO | Pokémon GO may serve as a potentially useful adjunct in promoting physical activity—and modestly increasing physical activity levels—and in enhancing mental well-being by reducing stress and fostering social connections, particularly among previously inactive individuals.   While the game may offer incidental health benefits, it also poses risks such as screen overuse, sleep disruption, and physical injury, underscoring the need for moderation and awareness. 27% of respondents admitting to sacrificing sleep, 20% considering themselves addicted, and more than half exceeding the World Health Organization (WHO) screen time guidelines based solely on the time spent playing Pokémon GO. There is a pressing need for long-term studies and interventions to evaluate sustained effects on physical activity. | Physical activity benefits and risk of PoGo | Not applicable |
| Goldbach [50] | 2017 | United States | News article |  |  | Not applicable | Not applicable | Not applicable | Every Monday, Wednesday and Friday, Wan, president of the CSUF Gaming and eSports club, and Amaro, club committee member, meet at the Titan sign in front of the TSU to walk to the arboretum to catch Pokemon.  Not only are the “Pokemon GO” walks helpful in maintaining healthiness, but Wan said he believes these walks can benefit students who are newer to the Cal State Fullerton campus and aren’t familiar with its layout. | Physical activity impacts of PoGo | Not applicable |
| Gómez-Cuesta et al. [51] | 2025 | Spain | Quasi-experimental design |  | Adolescents from two Compulsory Secondary Education (ESO) centers (146 active and 114 inactive) | Three days a week, in which they randomly used the Pokémon Go, MapMyWalk, Pacer, or Strava mobile apps after school for cardiorespiratory training | Physical Activity Questionnaire for Adolescents questionnaire (PAQ-A), Questionnaire of Mobile-Phone-Related Experiences (CERM), User version mobile application rating scale (uMARS), | Physical activity levels, problematic mobile phone use, quality of mobile applications | Among the apps analyzed, Pokémon Go had the highest abandonment rate among the inactive adolescents.  Special attention should be paid to active adolescents who travel a greater distance using mobile apps as this increase in the time required to complete a longer workout also appears to increase problematic mobile device use. | Risk of playing PoGo | Not applicable |
| Graells-Garrido et al. [52] | 2017 | Chile | Longitudinal analysis; observational | Data from before and after game’s release in August 2016 | Data from mobile phone networks | Not applicable | Device counts at each cell phone tower and zone level aggregation in the city of Santiago, Chile | Behavioral changes at the city level before and after the launch of Pokémon Go | On business days, there are more people on the street at commuting times, meaning that people did not change their daily routines but slightly adapted them to play the game. Conversely, on Saturday and Sunday night, people indeed went out to play, but favored places close to where they live.  Even if the statistical effects of the game do not reflect the massive change in mobility behavior portrayed by the media, at least in terms of expanse, they do show how ‘the street’ may become a new place of leisure. This change should have an impact on long-term infrastructure investment by city officials, and on the drafting of public policies aimed at stimulating pedestrian traffic. | Societal impacts of PoGo | Not applicable |
| Grajek et al. [53] | 2022 | Poland | Quantitative survey | March–June 2021 | Players of urban multimedia games (players of Pokemon GO, as the most popular application of this type) | Not applicable | Polish versions of Emotions and Sensations Related to Electronic Entertainment Questionnaire and Addiction to Electronic Forms  of Entertainment Questionnaire | Frequency of gameplay, physical condition, mental health | Users of urban multimedia games were usually adult men living in big cities.  It was also shown that the defined profile of the player was characterized by a higher risk of addiction to electronic forms of entertainment. It was observed that the respondents most often played urban multimedia games every day in a maximum of two-hour sessions. | Risk of playing PoGo | Not applicable |
| Guo et al. [54] | 2022 | United States | Quantitative survey | Between September 7, 2016 and December 12, 2016 | Users and non-users of Pokémon GO in the United States | Not applicable | Anonymous online questionnaire-based survey | Influence of Pokémon GO on frequency and choice of mode of transport on work and non-work trips | The modeling results identify four types of variables (attitude and perceptions related to Pokémon GO, app engagement, play style, and sociodemographic characteristics) that affect users’ travel behavior. The results illustrate that such apps with integrated AR, gamification, and social components can be used by policymakers to influence various aspects of travel behavior.   Providing in-app benefits through fixed- and dynamic-location virtual objects can influence users’ work and non-work route choice decisions. | Societal impacts of PoGo | Not applicable |
| Guo et al. [55] | 2021 | United States | Observational | September 2018 and January 2019 | Users and non-users of Pokémon GO | Not applicable | 80-item anonymous online questionnaire; three unconditional sections for all participants and four conditional sections for some participants | Safety perception, driving and cycling related behaviours, average daily steps before and after using the app, perceived physical and mental health benefits | LAR gaming apps can potentially promote physical activity by encouraging people to walk more, increase social interactions such as app-related discussions, but also contribute to increased app-related distracted driving and cycling, app-induced driving, and unsafe driving behavior. | Risk, physical activity, and social impacts of PoGo | Self-report questionnaire |
| Hamari et al. [56] | 2019 | International | Quantitative survey | 14 September–13th October 2016 | People who currently played or had recently played Pokémon Go | Not applicable | All the constructs used in the survey instrument were adapted from prior literature related to either uses and gratification or technology acceptance-related research as well as further adapted to fit the context of the study except for the outdoor activity construct which was developed by the authors due to a lack of related prior measurement in literature. | Challenge, Competition, Enjoyment, Trendiness, Socializing, Outdoor activity, Ease of use, Privacy concerns, Nostalgia effects on Intention to reuse and In-app purchase | The results indicate that game enjoyment, outdoor activity, ease of use, challenge, and nostalgia are positively associated with intentions to reuse (ITR), meanwhile outdoor activity, challenge, competition, socializing, nostalgia and ITR are associated with in-app purchase intentions (IPI). In contrast with our expectations, privacy concerns or trendiness were not associated with reuse intentions or IPI. | Motivation to continue playing and spend PoGo | Not applicable |
| Harborth & Pape [57] | 2017 | Germany | Quantitative survey |  | Users of the game | Not applicable | Questionnaire constructs are adapted from the original UTAUT2 paper | Performance Expectancy, Effort Expectancy, Social Influence, Facilitating Conditions, Hedonic Motivation, Price Value, Habit, Behavioral Intention, Use Behavior | The strongest predictor of behavioral intention to play Pokémon Go is hedonic motivation, i.e., fun and pleasure due to playing the game. Additionally, we find medium-sized effects of effort expectancy on behavioral intention, and of habit on behavioral intention and use behavior.   These results imply that AR applications – besides needing to be easily integrable in the users’ daily life – should be designed in an intuitive and easily understandable way. | Motivation to play PoGo | Not applicable |
| Harborth & Pape [58] | 2020 | Germany | Quantitative survey | January 2017 | Active German players | Not applicable | Constructs of the questionnaire adapted from Technology Acceptance Model (TAM) for hedonic information systems | Habit, Performance expectancy, Effort expectancy, Social influence, Hedonic motivation, Price value, and Facilitating conditions effects on behavioual intention and use behaviour | Results indicate that the effect of childhood brand nostalgia on behavioural intention is fully mediated by the belief constructs. Thus, nostalgic feelings about Pokémon influence the intention of users through altering beliefs concerning Pokémon. | Motivation to play PoGo | Not applicable |
| Harborth & Pape [59] | 2018 | Germany | Quantitative survey |  | Pokémon Go Players in Germany | Not applicable | English questionnaire was translated into German with by a certified translator | Study I: Privacy concerns with regard to organizational information privacy practices  Study II: Actual privacy protecting measures, which active Pokémon Go players undertake. | Study I: The majority of the active players are concerned about the privacy practices of companies. This result hints towards the existence of a cognitive dissonance, i.e. the privacy paradox.  Study II: The results are highly mixed and dependent on the measure, i.e. relatively many participants use privacy-preserving measures when interacting with their smartphone. This implies that many users know about risks and might take actions to protect their privacy, but deliberately trade-off their information privacy for the utility generated by playing the game. | Risky behaviours of PoGo players | Not applicable |
| Harborth and Pape [60] | 2017 | Germany | Quantitative survey |  | Users of the game | Not applicable | PLS-SEM approach based on the UTAUT2 model by Venkatesh et al. | Technology acceptance factors of the AR smartphone game Pokémon Go | The strongest predictor of behavioral intention to play Pokémon Go is hedonic motivation, i.e. fun and pleasure due to playing the game. Additionally, we find medium-sized effects of effort expectancy on behavioral intention, and of habit on behavioral intention and use behavior. | Factors affecting intention to play PoGo | Not applicable |
| Hino et al. [61] | 2019 | Japan | Longitudinal analysis | 2016-2017 | Japanese adults 40 years and above   46 (5.0%) were game players. Among nonplayers, 184 participants, which was equivalent to 4 times the number of players, were randomly selected so that the ratio of players to non-players was 1:4 after stratification by sex, age group (<55, 55-64, and ≥65 years), and PA level | Not applicable | Pedometer data 1 month before game release compared with 8 months after release  Questionnaire | Step count and play frequency | Middle and older-aged players’ step counts were significantly higher even 7 months after the release of the game, which was not observed in previous short-term studies.  The player group maintained their step counts in winter, despite the decrease in step counts of nonplayers. In subgroup analyses, players were more likely to be men, aged <55 years, workers, active, and subjectively in good health. | Physical activity impacts of PoGo | Pedometer |
| Howe et al. [62] | 2016 | United States | Quantitative survey and observational |  | United States participants of Amazon Mechanical Turk | Not applicable | “Health” application of the iPhone 6 series smartphones and reported by the participants via online questionnaire | Number of daily steps taken each of the four weeks before and six weeks after installation of Pokémon GO | The daily average steps for Pokémon GO players during the first week of installation increased by 955 additional steps, and then this increase gradually attenuated over the subsequent five weeks. By the sixth week after installation, the number of daily steps had gone back to pre-installation levels.   No significant effect modification of Pokémon GO was found by sex, age, race group, bodyweight status, urbanity, or walkability of the area of residence. | Physical activity impacts of PoGo | Wearable/Phone |
| Hsiao & Tang [63] | 2020 | Taiwan | Quantitative survey | March 17 to March 31, 2017 | Undergraduates of a university in northern Taiwan who are actual players of Pokémon Go | Not applicable | Survey was conducted by a trained instructor during regular class time with the permission of the students and teachers  All of the eight research constructs were measured with items modified from the validated instruments | Gamified experiences, Visiting intention and Continuance intention | Stimulus effects, such as social stimuli (critical mass and social interaction) and media stimuli (content timeliness and media richness), have significant impacts on the players’ internal gamified experience (attachment and conformity), which in turn affect their visit intention to catch creatures at certain attractions and to continue playing Pokémon Go.   Players’ intention to visit Pokémon spots is significantly correlated with their intention to continue playing the game. | Successful PoGo game features and intention to play | Not applicable |
| Hsieh & Chen [64] | 2019 | Taiwan | Randomized controlled trial | September 2017 to November 2017 | Taiwanese primary students in the fifth and sixth grade | Control group did not use Pokémon GO Experimental group used Pokémon GO for 10 weeks. | To assess memory, an ad hoc test of 90 seconds was used. Selective attention and concentration capacity were assessed under stress induced by a stipulated completion time, using the Attention Test for Children of Elementary School The measure for assessment of creative imagination was adapted from the researcher’s dissertation, The Test of Creative Imagination Trait and Emotional Intelligence Questionnaire Short Form | Ccognitive performance (memory, selective attention, concentration, and creative imagination) and emotional intelligence (well-being, self-control, emotionality, and sociability) | Compared against their peers, the players playing Pokémon GO showed a significant increase in their selective attention, concentration levels, creative imagination, emotionality, and sociability levels but not memory, well-being, or self-control.  With an average of 40 minutes play per day, boys had more fun in this game than girls. Boys were more involved in daily game practice, accumulating more points, and reaching a higher level than girls. Boys and girls both preferred to have company when playing, which made them happier, more motivated to go out, and more willing to test new versions of the game.  Pokémon GO, in a playful way, could positively affect their cognitive performance (selective attention, concentration levels, and creative imagination) and improve their social relationships. | Cognitive and affective benefits of PoGo | Not applicable |
| Huțul et al. [65] | 2024 | Romania | Quantitative survey | Between 3 January 2021, and 7 February 2021 | Various online groups from Facebook and Discord intended exclusively for PoGo players in Romania | Not applicable | Scales we used were translated from English into Romanian using the backward method and modified in accordance with the suggestions existing in the literature.  PoGo Motive Scale, Social Capital Scale, UCLA Loneliness Scale, Miller Social Intimacy Scale, Tromsø Social Intelligence Scale, Self-Perceived Communication Competence Scale | The reasons why people play PoGo, social capital, loneliness, social intimacy, social intelligence, communication competence | People play PoGo for nostalgia, physical movement (exercise), and fun. Nostalgia has activated adaptive coping mechanisms during the COVID-19 lockdown and state of emergency, serving as a protective factor for mental health.  Individuals who played PoGo to initiate new relationships reported lower levels of loneliness. Individuals who played PoGo with the intention of initiating new relationships reported higher levels of the bonding type of relationships. In addition to strengthening existing close relationships, they also have a desire to form new ones. | Motivation to play PoGo and benefits during COVID-19 lockdown | Not applicable |
| Ivan [66] | 2017 | Not mentioned | Conference paper, qualitative survey |  | Pokémon Go players | Not applicable | 10 open-ended questions | Opinions on the potential benefits of Pokémon Go | Pokémon Go encourages socialization, offering people a reason to spend time together. Even if the game has not been created to treat depression and anxiety, it is exciting for many users with such problems.  Pokémon Go was not a financial strategy, but has become a financial success; it was not a means to combat sedentary lifestyle, but movement has become an effect of the game. Mobile apps that combine game with physical activity lead to significant improvement of the latter, but the real challenge is to change one’s behaviour and maintain it in the long term. | Physical activity impacts and social benefits of PoGo | Not applicable |
| Jang & Liu [67] | 2020 | Germany | Quantitative survey |  | Pokémon Go users | Not applicable | Adopted the established scales to the research context, assessing: Content gratification, Process gratification, Social Gratification, Technology gratification, Game knowledge, and Achievement | Continuance use intention of playing Pokémon Go | Content gratification (i.e. catching Pokémon), process gratification (i.e. entertainment), game knowledge and achievement drive players’ continuance use intention. However, social and technology gratifications do not influence players’ continuance use intention. | Understanding continuing/discontinuing PoGo | Not applicable |
| Jenny & Thompson [68] | 2016 | United States | Quantitative and qualitative survey |  | Students and staff of Winthrop University | Not applicable | Six demographic questions, two open-ended questions about the perceived positives and negatives of Pokémon Go, and four final open-ended event-specific questions | Perceived positives and potential negatives of playing Pokémon Go, perceptions of the event | Perceived positives included: (a) more walking/exercise, (b) better body confidence, (c) getting outside/out of the house more often, (d) socializing while playing, and (e) it is a fun game to play with friends. Perceived potential negatives of playing Pokémon Go included: (a) not paying attention to surroundings, (b) dangers of getting hit by a car, (c) data usage, and (d) draining battery power.  Pokémon GO motivated participants to want to attend in an event where they would have to be active through walking and performing station physical activities. However, while Pokémon GO and other AR games may play an important role as a motivation tool to accomplish some exercise, overall event design and activity stations must be planned effectively to optimally reach the program’s physical activity aims. | Physical activity benefits and risk of PoGo | Not applicable |
| Joseph & Armstrong [69] | 2016 | United States | Case report |  | Pokémon Go players | Not applicable | Injuries sustained secondary to engagement with this augmented reality-based application | Not applicable | This technology holds tremendous promise to promote ambulatory activity. However, there exists the obvious potential for distraction-related morbidity | Risk of playing PoGo | Not applicable |
| Jumareng et al. [70] | 2022 | Indonesia | Mixed methods analysis | November to December 2021 | Teenagers in grades 10‑12 from three high schools | Control group did not get any physical activity | International Physical Activity Questionnaire, in-depth interviews analyzed by 3 experts who have Ph.D. degrees in physical education and sports | Physical activity level, Experience of Intervention, Pokemon Go Program Advantages, The disadvantages of Pokemon go program | Pokemon Go intervention program showed to positively promote PA levels in teenagers to higher levels in the current COVID-19 crisis  Teenagers with the status of players, ex-players and non-players experienced a significant increase in physical activity level after participating the Pokemon Go intervention program for 7 weeks, and the majority of subjects considered that Pokemon Go had a positive effect to promote physical activity during the COVID-19 pandemic. | Physical activity impacts of PoGo | Self-report questionnaire |
| Kaczmarek et al. [71] | 2022 | Poland | Quantitative survey |  | Pokémon Go (PoGo) players | Not applicable | Self-report questionnaire | Problematic PoGo use, Problematic smartphone use, phubbing, visual symptoms, musculoskeletal symptoms, Pokémon Go gaming time, media usage time | PoGo players with problematic game use reported more pain and vision problems. Problematic PoGo use was a better predictor of physical symptoms than PoGo gaming time and variables related to electronic media use. Problematic PoGo use and problematic smartphone use were correlated but independent predictors of physical symptoms.   The type of participation rather than the time spent playing predicts poorer physical health among PoGo players. | Risk of playing PoGo | Not applicable |
| Kaczmarek et al. [72] | 2017 | Not mentioned | Quantitative survey; longitudinal analysis | September to November 2016 | Pokémon GO players | Not applicable | International Physical Activity Questionnaire, Online Gaming Motivations Scale | Physical activity and gaming motivation | Individuals who spent more time playing Pokémon GO were more physically active; evidenced a new Pokémon GO playing behavioral benefit, i.e., increased time spent outdoors among more active players.   Men played more and derived more benefits from Pokémon GO. Players who appreciated social aspects of Pokémon GO (e.g., keeping in touch with their friends or being a part of a team) spent more time playing. | Physical activity impacts and social benefits of PoGo | Self-report questionnaire |
| Kari et al. [73] | 2017 | International | Quantitative survey | Mid-July to mid- November 2016 | Pokémon GO players | Not applicable | Qualitiative survey; Critical incident technique (CIT) to address users’ actual experiences (instead of hypothetical scenarios) | Types of behavior changes Pokémon GO players have experienced | Identified eight types of behavior changes: added activity in life, enhancing routines, exploration, increased physical activity, strengthening social bonds, lowering social barriers, increased emotional expression, and self-treatment  The behavior changes induced by Pokémon GO are not just restricted to increased physical activity or social behavior but are actually much more multifaceted: players were more social, found their routines more meaningful, expressed more positive emotions, and were more motivated to explore their surroundings. | Physical activity, social, and affective benefits of PoGo | Self-report questionnaire |
| Kato et al. [74] | 2017 | Japan | Editorial |  |  | Not applicable | Not applicable | Not applicable | Many shut-in individuals are starting to go out with Pokémon GO by their own motivation without any external pressures, providing a ‘first step’ towards more permanent solutions.  Behind the pathology of hikikomori lie difficulties in interpersonal exchanges and deeper psychological problems beyond the scope of these augmented reality games. There is also a risk of these games creating further problems.  Enhancing continuous self-motivation to go out and to enter therapeutic situations and society using such augmented reality technologies may be a novel therapeutic strategy that provides ongoing benefits in the process of treating hikikomori. | Social benefits of PoGo (potential) | Not applicable |
| Khalis & Mikami [75] | 2018 | Canada | Quantitative survey | 2016–2017 academic year | 101 Pokémon Go (PoGo) players from a public Canadian university | No instructions were given for the gameplay session, except that all participants received a small incentive (a candy bar) for either: (a) catching at least 15 Pokémon; or (b) visiting at least 10 Pokéstops during the session. | Questionnaires in the lab regarding their personality, social anxiety, and social competence | Personality, Social anxiety, Social competence, PoGo gameplay behaviour | Participants with greater social competence, agreeableness and extraversion as well as lower social anxiety were observed to catch more Pokémon and gain more experience points during gameplay. Participants with greater social competence and conscientiousness were observed to visit more Pokéstops and cover greater physical distances.   Personality and adjustment factors may influence behaviors in video games, much in the same way they do in face-to-face contexts. | Factors affecting PoGo behaviour | Not applicable |
| Khalis et al. [76] | 2022 | Canada | Quantitative survey | 2017–2018, approximately 1 year after the initial release of Pokémon GO | Pokémon GO player dyads from the undergraduate student body at a public Canadian university | The dyad was instructed to play Pokémon GO together eight times for a minimum of 15 minutes each time over the next 2 weeks. | Baseline questionnaire regarding noxious mood states and relationship closeness with their gameplay partner.  After each gameplay session, participants independently completed a short online questionnaire about their social interactions with their partner during gameplay, satisfaction with their gameplay accomplishments, and positivity. | Discrete, short-term associations between gameplay experiences and positivity that occurs immediately after the gameplay | More positive in-game social interactions (as well as greater satisfaction with game accomplishments) were associated with greater positivity post-gameplay  These findings align with the UGT, suggesting that players of Pokémon GO may derive affective benefits through the fulfilment of needs for competence and sociability within their gameplay sessions. Particularly interesting is the finding that this association may be especially pronounced for players with high depressive symptoms. | Social and affective benefits of PoGo | Not applicable |
| Kim et al. [77] | 2020 | United States | Conference paper, quantitative survey | December 2018 and February 2019 | Avid Pokémon GO players | Not applicable | Pre-survey was designed to examine participants’ healthy and unhealthy behaviors related to playing Pokémon GO, perceptions of the current health-related features in Pokémon GO, and design considerations for health-related features for Pokémon GO.   Informant workshops to incorporate players’ expertise with Pokémon GO and their lived experiences with the benefits and challenges to their health.  The post-survey consisted of questions including lessons participants learned from the workshop. | Prominent ideas, the overall atmosphere, and noteworthy conversations for data analysis. | Four design tensions: (1) diverse goals and rewards vs. data accuracy, (2) strong bonds between players and characters vs. gaming obsession, (3) collaborative play vs. social anxiety, and (4) connection of in-real-life experiences with the game vs. different individual contexts. | Successful PoGo game features (motivation) | Not applicable |
| Kim et al. [78] | 2020 | United States | Quantitative survey | 2018 | Undergraduate Pokémon GO users | Not applicable | Presence was measured with six items based on the conceptual definition of presence in previous research, along with Gameplay enjoyment, A sense of community belonging, and Exploring the community | Sense of community belonging | A feeling of presence increases an individual’s enjoyment of Pokémon GO gameplay, and increased enjoyment promotes exploring the community. In return, this ultimately leads to building a stronger sense of community belonging. | Social and affective benefits of PoGo | Not applicable |
| Kim et al. [79] | 2018 | Korea | Quantitative survey |  | Pokémon GO players | Not applicable | Man-to-man field survey and online survey; measures used are primarily from the literature on games | Enjoyment, Attitude, Intention to use | Self-efficacy had a non-significant effect on attitude toward the game Pokémon GO, while previous studies found that self-efficacy is the most important factor in increasing physical activity. This indicates that playing AR drives physical activity, subconsciously and effectively.  Physical activity increases subconsciously regardless of self-efficacy level when people’s intention is to enjoy a game. Thus, even if a game is developed to promote physical activity, it should make users feel enjoyment for the game itself but not consciously exercising more. | Physical activity impacts of PoGo | Self-report questionnaire |
| Kogan et al. [80] | 2017 | United States | Quantitative survey | 8/22/2016 to 8/23/2016 | Adult, dog owning, US Pokémon GO players | Not applicable | Details about their playing habits, daily exercise routines, and interactions with their family and dog, and how these were impacted by playing the game | Social interactions, physical activity, mental health and anxiety | Playing Pokémon GO affected the amount of time players spent with other people and pets, with 43.2% of respondents reporting an increased amount of time spent with family/household members and 52.3% reporting they spent more time with their dog. Additionally, 62.9% of participants reported spending more time walking their dog since starting to play.   A statistically significant increase in amount of exercise obtained was found when comparing pre-to post-game levels. A number of respondents reported feeling less anxious leaving the house (38.6%), interacting with strangers (39.7%), and going to new places (39.4%) after they began playing Pokémon GO. | Physical activity impacts and social benefits of PoGo | Self-report questionnaire |
| Koh et al. [81] | 2017 | United States | Quantitative survey | November 2016 to April 2017 | Study 1: Student from a large southern university in the United States  Study 2: US workers from Mturk | Not applicable | Web-based survey assessing intention of playing Pokemon Go while walking, automaticity tendency, immersion tendency, enjoyment, and 3 TPB components, including attitude, subjective norms, and PBC | Intention of playing Pokemon Go while walking | The following 3 TPB variables were significant predictors of intention to play Pokemon Go while walking in study 1 and study 2: attitude, subjective norms, and PBC. Automaticity tendency, immersion, and enjoyment were significant predictors in study 1, whereas enjoyment was the only significant predictor in study 2.  Younger users who are habitual, impulsive, and less immersed players are more likely to intend to play a mobile game while walking. | Factors affecting intention to play PoGo | Not applicable |
| Koivisto et al. [82] | 2019 | International | Conference paper, quantitative survey | September to October 2016 | Pokémon Go players | Not applicable | Survey assessing Achievement, Immersion, and Social Interaction | Perceived mental, physical and social health outcomes | The way in which players approach the game and what kinds of aspects of the game they emphasize can have a differential dynamic on how the health benefits of the game manifest.  Social gaming orientation is positively associated with physical, mental and social health outcomes, whereas achievement and immersion orientations are most strongly associated with physical and mental health outcomes from playing Pokémon Go. | Factors affecting health benefits of PoGo | Not applicable |
| Koroleva et al. [83] | 2016 | International | Commentary |  |  | Not applicable | Not applicable | Not applicable | The game already quite accurately satisfies the psychological needs of teenagers. The app facilitates the socialization of teenagers. Players will be more likely to engage in live communication during the game, including in new environments and new places. The game is an accessible and safe way of testing oneself. It provides the teenager with a way of understanding who he is and what he is capable of while insulating himself from the consequences of making mistakes. The virtual badges in the game allows for a visual record of success and comparison with others, as well as achieving status on a team, thereby allowing one to form their own identity. | Social and psychological benefits of PoGo | Not applicable |
| Kosa & Uysal [84] | 2021 | United States | Quantitative survey |  | Pokémon Go players | Not applicable | Player Experience of Need Satisfaction Scale, 2 items on Intention to purchase, 4 items on Intention to play, 1 item on Prior experience | Intention to purchase and play | Relatedness satisfaction uniquely predicted intention to make in-game purchases, whereas autonomy and competence satisfaction uniquely predicted intention to play.  Prior player experience moderated the relationships between, relatedness and intention to purchase, as well as, autonomy and intention to play. The effects were stronger for inexperienced Pokémon Go players. | Factors affecting intention to play and spend in PoGo (motivation) | Not applicable |
| Krittanawong et al. [85] | 2017 | International | Brief report | August 1 and September 10, 2016 |  | Not applicable | Twitter (https://twitter.com/) postings containing the terms “Pokémon” and “walking”, “walks”, “walk”, “walked”, “kilometer”, “kilometers”, “km”, “mile”, or “miles” (n =10,007) were obtained | Not applicable | 12% of tweets indicated that they walked between 2 and 35 miles a day playing Pokémon Go, suggesting that they were walking at least 30 minute a day, which met the current guideline recommendation. Augmented reality games such as Pokémon Go may enhance physical activity and perhaps reduce CVD risks. | Physical activity impacts of PoGo | Online self-report data |
| Ku et al. [86] | 2021 | Taiwan | Interview |  | Pokémon Go players | Not applicable | Soft laddering conducted in three steps: identifying attributes, building linkages between abstractions, and drawing the hierarchical value map (HVM) | Pokémon Go potential attributes, consequences, and values, and to construct a HVM | Pokémon Go users pursue social relationships through play, and these relationships are triggered by the benefits of making new friends, maintaining current relationships with friends and family, and the attributes of prevalence, childhood memory, game design, and augmented reality | Social benefits of PoGo (successful features) | Not applicable |
| Kumparak [87] | 2016 | International | News article |  |  | Not applicable | Not applicable | Not applicable | The company, Niantic, took its first swing at the problem: daily bonuses. The more regularly you play, the more you’re rewarded. | Successful PoGo game features | Not applicable |
| Kurland [88] | 2016 | United States | News article |  | Jews | Not applicable | Not applicable | Not applicable | The particular Pokémon appearing at Helena Rubinstein Auditorium, which features testimonials from Jews who survived gas chambers — though the cartoon creatures vary and change — is called a Koffing, which can be seen emitting gas from its round floating body, marked with a cartoonish spoof of a skull and crossbones.  The game gets you outside, but you’re still on your phone, and likely distracted from harm and stressed about playing. | Risky behaviours of PoGo players and social sensitivity issues | Not applicable |
| Laato et al. [89] | 2020 | Finland | Observational |  | Finnish Pokémon GO players | Not applicable | Developed a scale to understand player's attitude government and developer measures during the pandemic | Factors influencing intention to play LBGs socially during the on-going pandemic | Perceived severity of the pandemic and a positive attitude towards both governmental measures and in-game changes for combatting COVID-19 predicted intention to reduce social playing. Fear of missing out and deficient self-regulation increased playing intensity, which in turn negatively correlated with the intention to reduce social playing.  Location-based games can be considered a resource in designing interventions for influencing movement at a population level. | Societal impacts of PoGo | Not applicable |
| Laato et al. [90] | 2021 | Finland | Quantitative survey | April 8 to 14, 2020 | Finnish Pokémon GO players | Not applicable | Scales for Deficient self-regulation, FoMo, Psychological well-being, Fatigue, Playing intensity, Engagement with individual, cooperative, competitive game mechanics  Two-factor theory of psychological well-being | Psychological well-being and fatigue | Deficient self-regulation and fear of missing out to be positively associated with gaming fatigue. Engagement with cooperative and individual game mechanics had a positive relationship with well-being. Competitive game mechanics were found to have a positive relationship with both well-being and fatigue. Finally, the overall playing intensity had a strong relationship with well-being, but no association with fatigue. | Well-being impacts of playing PoGo | Not applicable |
| Lalot et al. [91] | 2017 | Various English- and French-speaking countries | Longitudinal analysis | August to December 2016 | Players recruited from social media and notably through various Pokémon Go Facebook groups | Not applicable | Online questionnaire; Short version of the HEXACO 6-trait personality inventory; trait impulsivity; need for closure; competitiveness; need for cognition; self-efficacy; reason for quitting | Distance walked per day while playing the game, as an indicator of game-induced physical activity in the medium to long term and, most importantly, the fact that participants were or were not still playing at Phase II (December 2016) | No personality traits predicted the distance walked by the players. However, the probability of still being playing the game at Phase II was positively predicted by three personality traits: agreeableness, perseverance, and premeditation. Distance walked per day significantly decreased between Phases I and II but remained substantial. | Personality traits and motives of PoGo players | Not applicable |
| Langford et al. [92] | 2019 | United States | Observational | August till November 2016 | Local Middle Tennessee participants | Not applicable | SenseWear Armband (SWA); questionnaires assessing current Pokémon Go usage, PA level, and health risk classification | Daily energy expenditure (DEE), self-reported physical activity, motivational factors | DEE was not significantly different for players (2735 ± 666 kcal) versus non-players (2274 ± 474 kcal), when controlling for body weight [F (1, 20) = 2,195, η = 0.114, p = 0.157]. In conclusion, interactive mobile games (i.e. PoGo) may be an effective method to increase DEE in individuals interested in mobile gaming. | Physical activity impacts of PoGo | Multisensor physical activity monitor |
| Laor [93] | 2022 | Israel | Quantitative survey | September 2019 | Pokémon Go Players | Not applicable | To understand their activity in relation to Yee’s model of motivations and while relying on the Motives for Online Gaming Questionnaire (MOGQ) | Pokémon Go playing motives and needs | It was found that there are more Pokémon Go players among young, secular men from central Israel, who are highly educated and possess middle-income and above.   It was found that Pokémon Go provides players with a number of needs: Escapism, the central and leading motive; Achievement; and Relationships. However, the weight of these motives differs from network games. | Motivation to play PoGo | Not applicable |
| Laor [94] | 2020 | Israel | Interview | 2018 | Pokémon Go players | Not applicable | The questionnaire contained a list of questions yet allowed and encouraged deviations and adjustments. | Unique motivations for playing AR games | Pokémon Go satisfies diverse player needs, and players’ main uses of the game are related to escapism, social interaction, and challenging others, consistent with previous research.  Two additional motivations of Pokémon Go players were identified, which are also applicable to AR use in general: increased physical activity and location-dependent escapism integrated into everyday activities outside the home. This consumption pattern is apparently part of the revolution of data accessibility online and on mobile phones, which also function as social means for satisfying needs for entertainment, escapism, and highly accessible information. | Motivation to play PoGo | Not applicable |
| Lawler [95] | 2018 | International | News article |  |  | Not applicable | Not applicable | Not applicable | The Adventure Sync option ties into Google Fit or Apple Health tracking to dole out bonuses based on all the movement players do throughout the day. That should mean earning more Buddy Candy and hatching Eggs, all without even opening the app. | Successful PoGo game features | Not applicable |
| Lawler-Sagarin et al. [96] | 2025 | International | Quantitative survey | August, 2018, and November, 2019 | Pokémon GO players | Not applicable | 16 questions including demographics and Pokémon Go demographics. | Personal demographics, game-related demographics, sense of belonging to the community where participants play Pokémon Go, and questions regarding whether participants have met someone, visited a new location, or patronized a new business through playing Pokémon Go. | Pokémon Go level significantly predicted sense of belonging to the community in which participants play Pokémon Go as well as the odds of meeting someone, visiting a new location, and patronizing a new business. Results suggest that Pokémon Go strengthens players’ sense of belonging and engagement with their community. | Social benefits of PoGo | Not applicable |
| Lee & Lin [97] | 2024 | International | Video analysis | Earlier than 2024, from game launch in 2016 |  | Not applicable | Thematic analysis of 242 user-generated videos of augmented reality Pokémon GO gameplay using a coding scheme to identify the threats to individuals and societies | Threats, nuisances and disregards to users, bystanders, properties, and various spatial contexts (e.g., public, private, and sensitive spaces). | Video analysis generalises the threats, including but not limited to hitting bystanders, falling off a cliff, car crashes, crowds and blockages, and stampedes.  The top-five threat issues in descending order are Crowd/Swarm (15.2%), Bystander Response (13.1%), Stampedes (10.6%), Block (8.9%) and Trespassing (8.8%). In particular, the issue of crowd/swarm brings disturbance and inconvenience to other stakeholders in the public space, especially road users in the roads, while the issues of bystander response, stampedes, and block are associated with a major portion of incidents in parks and leisure areas. Additionally, personal threats are almost four times less than social-spatial threats, but most personal threats have been recorded in the public space (18.1% out of 21.0%), implying that most AR gameplay incidents, regardless of the threat category, have had ineluctable influences on society. | Risky behaviours and socio-spatial impacts of PoGo players | Not applicable |
| Lee 2021 [98] | 2021 | International | Quantitative survey |  | Pokémon GO and Harry Potter Wizards Unite players | Not applicable | Survey before and after COVID-19 | Factors underlying their continued engagement, as well as players’ experiences of these factors affected any benefit they derived from play | A significant positive relationship between hours of participation in gaming and total hours of exercise per week was noted, coupled with a significant increase in the use of video games, suggesting that AR games continued to promote physical activity during the pandemic.  Qualitative results supported this, with achievement, entertainment, exercise and social connection being prominent motivations. | Physical activity impacts of PoGo and motivation to play | Self-report questionnaire |
| Lee et al. [99] | 2018 | Taiwan | Quantitative survey | February to April 2017 |  | Not applicable | This research develops a conceptual model and hypotheses based on the theory of flow and satisfaction to investigate the antecedents.  Validated multi-item scales were adapted from previous studies to measure research constructs. | Antecedents of Pokémon Go stickiness | Flow and satisfaction were found to have strong direct effects. In comparison to satisfaction, flow also has a stronger impact on stickiness, suggesting that flow is a significant reason why players continue to play Pokémon GO. Telepresence, challenge, perceived control, curiosity, and concentration all have direct influences on the flow. Only perceived currency and responsiveness were found to have a direct impact on players’ satisfaction. | Reasons to adopt, continue, or quit playing PoGo | Not applicable |
| Lemmens & Weergang [100] | 2023 | International | Quantitative survey | October 2019 | Pokémon Go players | Not applicable | The questionnaire consisted of approximately 90 items measuring player demographics, characteristics, motivations, game use and its appeal, microtransactions, and gaming disorder items. Specifically included age, gender, nationality, social anxiety, SDT-motivations, usage metrics, spending behavior, and gaming disorder. | Pokémon caught, Hours per week, Gaming disorder, Spending, Motivations | The need for in-game relatedness was the strongest predictor of playing, followed by the need for in-game competence. Male players showed stronger in-game motivational needs for relatedness and competence, whereas female players were more strongly motivated by a need for autonomy.   The needs for relatedness and competence predicted spending on microtransactions, whereas the need for autonomy did not. Depending on the measure for gaming disorder, between 1% and 9% of these core players met the diagnostic criteria. Gaming disorder was also related to social anxiety and increased spending on in-game microtransactions. | Factors affecting intention to play PoGo | Not applicable |
| Li et al. [101] | 2021 | International | Editorial commentary |  |  | Not applicable | Not applicable | Not applicable | Pokémon GO, as a widely popular, marketed product, can be seen as a potential indication to solve some health issues, especially given the growing sedentary lifestyles in the world.  These innovations including Pokémon GO for mhealth may provide more ways to consider the current health problem not just as a topic in academia, but a combination of behavior, market forces, and cultural influences. | Public health impact of PoGo | Not applicable |
| Li et al. [102] | 2020 | United States | Quantitative survey and Twitter analytics | 2017 | Pokémon GO users | Not applicable | Integrated the text analytics and survey-based theory-validating research methodology to build and test the research model. | User continuance and more use intention | The rational risk/benefit calculus and satisfaction are two primary inputs for continuance intention. Besides physical health benefits, users also value the benefits in mental health and relationship building. The risks in performance, time and safety are salient risk dimensions that negatively impact satisfaction. Technical features play a strong role in influencing perceived benefits and user satisfaction. | Reasons to adopt, continue, or quit playing PoGo | Not applicable |
| Lindqvist et al. [103] | 2018 | Sweden | Interview | December 2016 | Children and parents | Not applicable | Each family formed a focus group consisting of 1 or 2 children and 1 or 2 parents. The focus group interviews were performed by the first and last authors, and they were audio recorded and transcribed verbatim. | Children’s and parents’ experiences playing Pokémon GO | Three themes were revealed: (1) exciting and enjoyable exploration; (2) dangers and disadvantages; and (3) cooperation conquers competition.  The first centers around the present and possible future aspects of Pokémon GO that promote physical activity. The second focuses on unwanted aspects and specific threats to safety when playing the game. The third shows that cooperation and togetherness are highly valued by the participants and that competition is fun but less important. | Physical activity benefits and risk of PoGo | Not applicable |
| Liu et al. [104] | 2023 | International | Ground theory | Game release (May 2016) to Aug 2021 | Collected data on Pokémon GO’s monthly active player numbers at different times | Not applicable | Not applicable | “Spicy Gameplay” includes Exploration, Entanglement, Strategy, and Conflict. These are typically the most attractive and visually impactful  “Sticky Gameplay,” however, is some “soft” ones a player goes along with. Axial codes include Accompanied Progression, Co-fighting, Limited Time Offer, Routinely To-do and Check, Social, and Variation.  “Snug Components,” including Compatibility, Tweak, and Fix. This theme is the part hardly noticed by a playing person. But it may ruin the game experience extensively once not dealt with properly. | This study’s findings indicate that key changes in Pokémon GO (i.e., key features and quality of life updates) relate to the Jump Up and Brake events of the monthly active user number uptrends. These key changes can be put into clusters, namely the axial codes identified in the findings, based on their very natures. These clusters may be comparable to some game design patterns.  This paper proposes a theory of Geo AR mobile game success. Three game design components (Spicy Gameplay, Sticky Gameplay, and Snug Component) would contribute to three scenarios in the active user number trend (Initial, Jump Up, and Brake). These three scenarios could lead to the success Geo AR mobile game. | Successful PoGo game features (motivation) | Not applicable |
| Loveday & Flow [105] | 2017 | Australia | Quantitative and qualitative survey | September 2016 | Adult players of Pokémon GO | Not applicable | Validated survey instruments, measuring flow (Flow State Scale-2 Short Form) and nostalgia (Nostalgia Scale) and open-ended questions | Beneficial state of flow | Significant predictors for 27% of variance in flow levels of Pokémon GO players were: game level achieved, playing alone, nostalgia for Pokémon from childhood, and playing with family.   The themes identified by thematic analysis of participant comments concerned the beneficial effects they considered Pokémon GO was having on their social, mental, and physical well-being. | Motivation to play PoGo | Not applicable |
| Ma et al. [106] | 2018 | Hong Kong | Quantitative survey | August 2016 | Hong Kong residents who played Pokémon GO using iPhone 5 or 6 series in 5 selected types of built environment | Average daily walking and running distances over a period of 35 days, from 14 days before to 21 days after game installation | Participants’ data, including sex, age, income, education level, BMI (kg/m2), and the start date of Pokémon GO, were collected using a questionnaire.   Participants’ physical activity data, specifically daily walking and running distances, were captured from their iPhone “health” app pages by taking pictures of their screens | Changes in players’ physical activity levels | The average daily walking and running distances increased by 18.1% (0.96 km, approximately 1200 steps) in the 21 days after the participants installed Pokémon GO compared with the average distances over the 14 days before installation (*P*<.001). However, this association attenuated over time and was estimated to disappear 24 days after game installation.  Pokémon GO was associated with a short-term increase in the players’ daily walking and running distances; this association was especially strong among less physically active participants. Pokémon GO can build new links between humans and green space and encourage people to engage in physical activity. | Physical activity impacts of PoGo | Wearable/Phone |
| Macheel & Reosti [107] | 2016 | United States | Trade journal |  |  | Not applicable | Not applicable | Not applicable | Hyper engagement and addictiveness of the game is the sense of accomplishing a task and getting to the next level  People are more prone to make in-app purchases in Pokemon Go because they trust the brand, grew up with it, believe in it and want to interact with it using a different medium than they did in the past  There have also been concerns about safety and tresspassing, since the digital world the game creates does not always account for the laws and safety restrictions of the real world that it asks players to navigate. | Reasons to continue playing and spend PoGo and risky behaviours | Not applicable |
| Majgaard & Laarsen [108] | 2017 | Denmark | Conference paper, interview and quantitative survey | Autumn 2016 | Pokémon Go players | Not applicable | Semi-structured interviews and online questionnaire | Demographics of adult Pokémon Go players and the type of gaming community they shaped | Many younger and middle-aged women without a lot of gaming experience were among the respondents. Disproportionately greater interaction with strangers compare to other games. Vibrant user-initiated online communities and forums surrounding the game across multiple platforms. | Player demographic and societal impacts | Not applicable |
| Malik et al. [109] | 2020 | Finland | Observational |  | Current players of Pokémon Go | Not applicable | The instrument used in the study was developed from the relevant previous literature including measures for general gaming behavior, uses and gratifications, and concerns for privacy aspects intentions and enjoyment | The motivational, usage, and privacy concerns variations among age and gender groups of Pokémon Go players | Most of the players, who are likely to be casual gamers, are persuaded toward the game due to nostalgic association and word of mouth. Females play Pokémon Go to fulfill physical exploration and enjoyment gratifications. On the other hand, males seek to accomplish social interactivity, achievement, coolness, and nostalgia gratifications. Compared to females, males are more concerned about the privacy aspects associated with the game. With regard to age, younger players display strong connotation with most of the studied gratifications and the intensity drops significantly with an increase in age. | Player demographic and motivation to play PoGo | Not applicable |
| Manulife Financial Corporation [110] | 2016 | United States | News article | July 22 to July 28, 2016 | Millenials | Not applicable | Not applicable | Not applicable | 80% of those who play reported spending 30 minutes or more a day playing the game. Those who do not play also believes the app will increase their step count. 31% have used the app while driving or cycling, and 12% experienced some type of injury while playing. | Physical activity benefits and risk of PoGo | Self-report questionnaire |
| Marquet et al. [111] | 2017 | United States | Longitudinal analysis |  | Students from North Carolina State University who play Pokémon GO | Not applicable | International Physical Activity Questionnaire (IPAQ) Short Form  Step-counting app (PACER; Pacer Health Inc)   EMA app ([PACO]; Paco Developers) would prompt a brief questionnaire on playing behavior and physical activity 3 times per day (12:00 PM, 7:00 PM, and 10:00 PM) | Step count, playing behviour and physical activity, playing motivation  How playing Pokémon GO had made players more physically active, improved their mood, and increased social interactions | Interpretable factors from the clustering of motivations to start playing Pokémon GO: Pokémon and video game fans (n=26, 55% of the sample), physical activity seekers (n=8, 17%), and curious & social (n=13, 28%)  Days during which Pokémon GO was played were positively associated with a set of beneficial health behaviors, including higher physical activity levels, more socialization, and better mood. Results, however, depended on personal motivations and expectations when joining the game. | Physical activity, social, and affective benefits of PoGo and motivation to play (demographic) | Wearable/Phone and self-report questionnaire |
| Marquet et al. [112] | 2018 | United States | Quantitative survey | November 2016 | US college students | The EMA tool prompted a set of questions on playing behavior and physical activity (PA) three times per day (12pm, 7pm, 10pm) for seven days | Pre and post online survey and to install an Ecological Momentary Assessment (EMA) tool, PACO, and a step counter, PACER, on their smartphones  EMA questions on four determining factors of PA: time of the day during which the playing behavior occurs, environment in which playing takes place, social or solitary playing, and finally, what kind of activity takes place while playing. | Effect of (1) amount of playing time, (2) time of day during which playing took place, (3) playing while active or sedentary, (4) playing environment, and (5) the social component of playing, on PA. | Playing Pokémon GO was associated with higher PA when playing occurred during weekdays and during daytime and also among those who played while being active (i.e., walking). During weekends, this association was only found in the morning or late in the evening (after 7pm). Accumulating three or more active playing episodes per day was associated with an increase of 1526 daily steps.   Pokémon GO has uneven effects on player's PA. However, under the right circumstances such as the time of day during which playing occurs, or where the playing takes place, Pokémon GO can become a useful tool for health promotion among young adults. | Physical activity impacts of PoGo | Wearable/Phone and self-report questionnaire |
| Martínez-López et al. [113] | 2022 | Spain | Quantitative longitudinal study |  | Spanish adolescents between the ages of 12–15 years | Use Pokémon GO for 8 weeks | ALPHA health-related fitness test battery for youth  ASIMED B-type-class III and a portable height metre SECA 214 were used to record height and weight, respectively. | Fitness and fatness | Pokémon GO increased PA and cardiorespiratory fitness (CRF) levels and decreased BMI and body fat (%BF) after 8 weeks of practice independently of age, sex, number of computers at home, and maternal education. Despite these results, less than half of the participants considered that Pokémon GO had improved their overall fitness and felt more encouraged to engage in PA. Boys spent more time per day playing, accumulating points, and reaching a higher game level than girls.  The Pokémon GO app could be used in school and family contexts to increase the daily amount of MVPA, improve CRF, and to take advantage of the effects of loss of body fat. | Physical activity impacts of PoGo and player demographics | ALPHA health-related fitness test battery |
| Mateo-Orcajada et al. [114] | 2024 | Region of Murcia (Spain) | Quasi-experimental design |  | Two compulsory secondary education schools in the Region of Murcia | Prior to the intervention, the adolescents completed the PAQ-A questionnaire. During the intervention, adolescents were obligated to use the mobile applications at least three times a week, to complete the minimum distance established for each week. At the end of the intervention, the adolescents completed the PAQ-A, the uMARS and the dropout questionnaires. | Physical Activity Questionnaire for Adolescents (PAQ-A), User Version of the Mobile Application Ratings Scale (uMARS) | Physical activity, mobile apps rating | For Pokémon Go, a large percentage of teens who did not use it indicated that it was because the app did not work properly on their device; the navigation and credibility of source ratings were worse than in Strava and MapMyWalk, respectively, when considering the covariate distance travelled.   However, adolescents showed a higher engagement, entertainment, customization, and interactivity in Pokémon Go, as compared to Pacer and Strava, when considering the covariate distance covered. The covariate gender was also shown to influence the results of the objective evaluation, more specifically in Pokémon Go.  Pokémon Go does not provide any health-related content or information, as aspects such as playing, having fun, nostalgia, social ties or interest in Pokémon are the main drivers for increasing physical activity. | Player demographic and reason to quit playing PoGo | Steps recorded on each application’s interface |
| Mattheiss et al. [115] | 2017 | International | Quantitative survey |  | Participants from North America and Europe | Not applicable | The Big Five personality traits were assessed with 15 items. The three player motivation subscales “Social”, “Immersion” and “Achievement” were assessed with the 12-item scale. Also assessed personal innovativeness in Information Technology and hours played per week  The second survey was conducted to assess which participants continued playing the game after three months and which stopped | Personality characteristics that relate to adoption and continued play of the game | People who played the game score lower in “Conscientiousness” and higher in “Player Motivation” than nonplayers. People who continued playing the game three months later have a lower score in “Neuroticism” than those who stopped playing. | Personality traits and motives of PoGo players | Not applicable |
| Mejia et al. [116] | 2019 | Peru | Quantitative survey |  | Secondary school students | Not applicable | Addiction to using the Internet and video games was also evaluated with the MULTICAGE CAD-4 test | Socio-educational aspects, and Internet and/or video game addiction, problems at home, with teachers, accidents, and theft | 44% (409) had an Internet addiction and 23% (215) a video game addiction. With regards to the respondents who played Pokémon GO, 49% (336) spent a large time interacting with the game (two or more hours per day).  Almost half of the school´s students that participated in our study used Pokémon GO excessively and a high percentage of students were addicted to the Internet and video games. Higher usage levels of the game were associated with having a video game addiction, greater use of phone applications, negative repercussions from parents, and reprimand for arriving late to class. | Negative mental health correlates among PoGo players | Not applicable |
| Meneley [117] | 2019 | Palestine | Observational |  |  | Not applicable | Not applicable | Not applicable | Examined the relationship between self-monitoring and other-monitoring, especially in relation to walking in Palestine, and explored how genres of mobility like nature walking or playing Pokémon GO might unfold differently in an occupied territory where the right to move is highly contested.   In Palestine, walking becomes an important means not for pursuing personal health, but for cultivating a wider health of the land and knowledge of the nurturing relationship between land and the people who walk across it. Such practices of walking with or walking together can function as forms of kinwork. | Societal impacts of PoGo | Not applicable |
| Meschtscherjakov et al. [118] | 2017 | Germany | Quantitative survey |  | Active Pokémon GO players | Not applicable | Smartphone gaming behavior (5 items), as well as Pokémon GO playing behavior (10 items) including game statistics (8 items) from the Pokémon GO app | Changes in physical activity from playing | Pokémon GO persuades people to leave the home and increase exercise.  Despite fun being the main motivator for people to play it, game-design elements that allows players to collect new Pokémon and filling-up the Pokédex nudges people to go out more often and exercise. Game-design elements such as catching as many different Pokémon and completing the Pokédex are more persuasive than fighting and competition challenges.  The longterm effect on mobile behavior will be dependent on constantly updating the game and the introduction of new catchable Pokémon. | Successful PoGo game features (motivation) | Not applicable |
| Militello et al. [119] | 2018 | United States | Quantitative survey | January and February 2017 | Parent-child dyads | Not applicable | Participants were surveyed about family characteristics, interest, and experiences playing Pokémon GO and healthy lifestyle beliefs, from both parent and child perspectives.   Using a revised Godin Leisure-Time Exercise Questionnaire, a retrospective pre-post design assessed changes in parent physical activity (PA) before and after playing Pokémon GO. | Physical activity and user experience in gameplay | “Spending time together” was the most cited reason for gameplay by both parents and children. Interestingly, open-ended responses indicated that gameplay could trigger both positive and negative emotional parent response. The most cited reason for app disengagement was boredom; conversely, the most cited reason for app re-engagement was in-app events.  For parents, there were significant increases in minutes spent in mild and moderate PA per week after playing Pokémon GO. However, child perceptions of parental influence on PA most significantly associated with parents who reported weekly strenuous PA both before and after Pokémon GO uptake.   For a period of time, Pokémon GO fostered social and physical well-being for children and families through a multifaceted approach. | Physical activity impacts and social benefits of PoGo | Self-report questionnaire |
| Mukhra et al. [120] | 2019 | United States | Commentary |  |  | Not applicable | Not applicable | Not applicable | There have been numerous ethical and legal issues involved with the game including privacy, safety, ignorance, surveillance, addiction, accidents, hacking, and associated health hazards.   Though Pokémon Go has been associated with both positive and negative consequences, its utility value depends upon its acceptance by society in the right way. | Risk of playing PoGo | Not applicable |
| Muramatsu et al. [121] | 2019 | Japan | Case report |  |  | Not applicable | Not applicable | Not applicable | A 27‑year‑old male who sustained multiple injuries after being hit by a truck while playing Pokemon Go. A physiological study revealed a head contusion and left shoulder, left back, and hip tenderness. Whole‑body computed tomography revealed left clavicular, sternum, and left multiple rib fractures with lung contusions and iliac wing fracture. | Risk of playing PoGo | Not applicable |
| Narayan [122] | 2018 | International | Quantitative survey | 2017 | Active members of these Pokémon Go communities participated, which also include many who self-attested to suffering from social anxiety. | Not applicable | A mixed methods survey consisting of five qualitative open ended questions and six questions based on a five point Likert scale | Effect of Pokémon Go on social anxiety | The qualitative data collected from them expresses a high degree of acceptance and appreciation of Pokémon Go as a valid and helpful platform affecting social anxiety.  The game effectively fills in the shortcomings of known effective treatments by providing a completely self-motivated and self-regulated activity which provides just the right amount of cognitive load with its light gameplay. | Mental health impacts of playing PoGo | Not applicable |
| Nelson et al. [123] | 2024 | International | Conference paper, qualitative survey | Before 2 April 2024 | r/pokemongo subreddit’s official Pokémon Go (PoGo) discord with over 30,000 international members | Not applicable | Crafted a survey with questions informed by 125 comments on a PoGo subreddit post (800,000+ members) asking whether other members use PoGo’s AR features | AR usage behaviors, how these impact exercise, and why players do or do not use the AR features | Survey reveals that 48.4% of players do not engage with AR at all. Of those that do use the AR features, they only do so rarely (73% of AR users, or 37% of the total participants, only use AR <10% of the time). 25% of players engage with genuine AR experiences (AR+) to any extent.  Pokémon GO is the most played AR location-based exergame, but its "AR" label may be misleading. PoGo's success in encouraging prolonged walking is hindered by its AR features, which require players to stand still between destinations, interrupting their exercise flow. The survey shows this results in lower AR engagement and reduced physical activity compared to non-AR players. | Successful PoGo game features (and considerations; motivation) | Not applicable |
| Nemet [124] | 2017 | United States | Commentary |  |  | Not applicable | Not applicable | Not applicable | The increase in physical activity has not been shown to sustain over time. Some studies also highlight the negative effects of this extremely popular video game, stating that Pokémon GO might also entail risks, such as increased risk of injury, road traffic incidents, abduction, trespassing and violence.  There is a need for a more critical understanding of the physiological responses of playing such games including energy expenditure, compensatory behaviors in energy intake or a decrease in other activities. | Physical activity benefits and risk of PoGo | Not applicable |
| Nigg et al. [125] | 2017 | United States | Editorial | July 28, 2016, to August 31, 2016 | Pokémon GO players | Not applicable | Revised Godin Leisure-Time Exercise Questionnaire assessed days per week (0–7) and minutes per day (10-minute intervals from 0 to ≥ 60) spent in strenuous, moderate, and mild physical activity before and after beginning to play Pokémon GO. | Physical activity | Playing Pokémon GO increased moderate to vigorous physical activity by about 50 minutes per week and reduced sedentary behavior by about 30 minutes per day; significant increases for all three physical activity indicators with the largest change in the mild-intensity physical activity. For sedentary behavior indicators, TV, video, or DVD watching and Internet surfing decreased significantly.  Pokémon GO individually tailors its character (avatar) and augmented reality of the game both motivationally and geographically, maximizing uniqueness for each player, and may be providing a way to individualize interventions. | Physical activity impacts of PoGo | Self-report questionnaire |
| Nikou et al. [126] | 2018 | International | Conference paper, qualitative survey | 9th to 10th of March 2017 | Pokémon Go players using Reddit | Not applicable | Developed 10 statement-like questions to measure the five identified wellness dimensions, two questions per each dimension.  By subtracting the past wellness levels from the current wellness levels, the change in wellness levels for every specific dimension could be obtained. | Physical, social, intellectual, spiritual, emotional, and overall wellness | Positive relationship between playing Pokémon Go and intellectual, spiritual and emotional dimension of wellness. Did not find, however, any significant relationship between playing Pokémon Go and dimensions of physical and social wellness. This is quite surprising considering that the game developers have invested considerable effort to design in the Pokémon Go game features that should encourage physical activity, social encounters and group playing.  It seems that people enjoy playing the game and that emotional wellness is a meaningful dimension of wellness in the relatively young population of active Pokémon Go players. | Physical activity, social, and affective benefits of PoGo | Not applicable |
| Ojelabi [127] | 2018 | United States | Interview | 2017 | Students at several urban universities and colleges in the Midwestern region of the U.S. | Not applicable | Interview questions focused on examining their game experience, their motivation to play the game, their perceived benefits particularly concerning their health and social interactions, and their concerns or perceived barriers. | Social interaction and physical activity | Pokémon Go encouraged increased social interaction among family, friends and strangers. Participants also enjoyed some health and educational benefits as a result of playing Pokémon Go. AR technology, employed in the deployment of Pokémon Go on smartphones actually encourages and facilitates face to face communication. Participants met people at Pokéstops and Pokégym where they had face to face interaction about the game, life, and in some cases, they became friends over time.  Concerns of playing Pokémon Go borders on personal safety and security, circumvention of game design as well as privacy. | Social benefits of PoGo (successful features) and risk | Not applicable |
| Ono et al. [128] | 2017 | Japan | Brief report | June to 31 August 2016 | People in Japan | Not applicable | Population-based quasi-experimental study using national data from the Institute for Traffic Accident Research and Data Analysis, Japan. | Pokémon GO–related fatal traffic injuries | Non-significant change in incidence of fatal traffic injuries after the Pokémon GO release; effect of Pokémon GO on fatal traffic injuries may be negligible. | Risk of playing PoGo | Not applicable |
| Orosz et al. [129] | 2018 | Hungary | Quantitative survey | 2017 | Hungarian Pokémon Go players | Not applicable | Questionnaire; Motives for Online Gaming Questionnaire-Pokémon Go Extension (MOGQ-PoGo), Passion Scale, Short UPPS-P Impulsive Behavior Scale (SUPPS-P) | Motives of Pokémon Go players, passion, impulsivity | In line with the Dualistic Model of Passion, Harmonius Passion (adaptive) and Obsessive Passion (maladaptive) for playing Pokémon Go can predict an almost perfectly distinguished set of adaptive or maladaptive playing motives, and OP has a noteworthy relationship with impulsivity as a determinant. | Motivation to play PoGo | Not applicable |
| Pickett Corona [130] | 2018 | United States | Randomized controlled trial | January 26, 2018 to March 02, 2018 |  | On weeks 1 & 2 the participants were asked to wear the tracking devices and maintain their normal routine to collect a baseline, and for the weeks 3 & 4 they were randomized into  the 3 groups and asked to play the Pokémon Go game (following the criteria of each group) | Analyzing GPS tracking data from the Pokémon Go mobile application and Follow Mee Application  First phase was to gather participants to collect the information (players’ behavior, movement, trajectories, and  experience with the game) and the second phase was to analyze the data through different disciplines and areas of research | GPS data from each group for location and physical activity information | The Pokémon Go game could represent a fun way for the users who seek improve their health to change their traveling patterns and might increase their physical activity without thinking on the time spent doing it, by analyzing the Land Uses, the users are more likely to be taken to recreational open spaces to help develop or maintain a healthy lifestyle. | Physical activity impacts of PoGo | Self-report questionnaire |
| Ponce-Ramírez et al. [131] | 2024 | Region of Murcia (Spain) | Quasi-experimental design | Before May 2024 | A school was selected in the Region of Murcia with the largest number of adolescents enrolled in compulsory secondary education (CSE) in its municipality.  The sampling was non-probabilistic by convenience, through the selection of adolescents with a normal weight (BMI: 18.5–24.9) and overweight/obese (BMI: >25) available at the selected school. | A quasiexperimental design was carried out with two experimental groups, the first being composed of normal-weight adolescents and the second of overweight/ obese adolescents.  Participants in both experimental groups were randomly assigned to one of the selected mobile apps (Strava, Pacer, Map My Walk, and Pokémon Go). | Before the start of the intervention (pretest), participants completed the Questionnaire of Experiences Related to Mobile Phones (CERM).  The adolescents used the mobile physical activity apps for 10 weeks outside school hours. The proposed target for the first week was 4.5km each day they used the app, which corresponds to 7152 steps. The distance to be covered was progressively increased each week at a rate of 595 steps/week, ending with a total of 12,520 steps on the 10th week.  The mobile apps were rated by the adolescents by using the uMARS. | Adherence to the intervention with mobile physical activity applications; subjective assessment of mobile apps according to weight status and the mobile app used; problematic use of mobile phones; the relationship between distance travelled with mobile application use, ratings, and problematic mobile phone use | The results showed no significant differences in adolescents’ adherence to the intervention according to the mobile application used (*p* = 0.191) or weight status (*p* = 0.202). Significant differences were not found in the assessment of mobile applications within the group of overweight and obese adolescents: engagement (*p* = 0.471), functionality (*p* = 0.319), aesthetics (*p* = 0.378), information (*p* = 0.184), usability (*p* = 0.154), or perceived impact (p = 0 139), although differences were found in the assessment made by normal-weight adolescents in information (*p* = 0.029), usability (*p* = 0.029), and perceived impact (*p* = 0.044), where Pacer had better scores than Pokémon Go in the first two dimensions. No greater problematic mobile phone use was found after the intervention according to weight status (p = 0 311) nor the mobile application used (p = 0 985).  It can be concluded that there is similar adherence among normal weight and overweight/obese adolescents to interventions with mobile applications to promote physical activity. It is noteworthy that adolescents, regardless of weight status, showed a positive perception towards the use of these mobile applications. | Application of PoGo as a physical activity intervention | Steps recorded on each application’s interface |
| Qin [132] | 2021 | United States | Quantitative survey | 2020 | Pokémon Go players; undergraduates from a large eastern university in the U.S. | Not applicable | Player Experience of Need Satisfaction scale (Autonomy, Competence, Relatedness, Presence), Intrinsic Motivation Inventory, Attractiveness of Game Elements, and Length of Gameplay | Player perceptions of game elements and the satisfaction of the three SDT needs | Autonomy- and relatedness-supportive elements associated with autonomy and relatedness need satisfaction of the player, which related to presence significantly. The satisfaction of autonomy and competence needs related to game enjoyment, which influenced gameplay length.  The attractiveness of need supportive elements of Pokémon Go have need satisfying potentials. The satisfaction of these needs, in turn, leads to game enjoyment and continued gameplay. | Reasons to adopt, continue, or quit playing PoGo | Not applicable |
| Rasche et al. [133] | 2018 | Germany | Observational | 14 weeks after the official release of Pokémon Go™ in Germany | Active and former players | Not applicable | Assessed initial contact including use of the introduction tutorial in-game and other sources of informations. User behavior was evaluated including the time gamers spend playing Pokémon Go and whether they used the augmented reality function or performed in-app purchases. The experience of critical situations like crossing the street without paying attention to traffic was investigated. | Motivation to perform physical activity and intention to play this game | Active users prefer the virtual reality visualization. Former users stated to use the augmented reality function rarely. The choice of visualization mode was independent from experienced critical situations. No negative influence was determined of the perceived quality of the initial contact regarding duration of use. Former gamers stated to play Pokémon Go™ in cases they were already physical active. Active gamers stated to take time out and play it consciously. About 10% of this sample experienced a critical situation during playing. | Player demographic and risk of playing PoGo | Not applicable |
| Rasche et al. [134] | 2017 | Germany | Quantitative survey |  | Indicated on Facebook that they were interested in physical activity and well-being, entertainment electronics, or Pokémon Go | Not applicable | Web-based survey regarding game experience, physical activity, motivation, and personality as measured by the Big Five Inventory | Physical activity, motivation, personality | Active users are more motivated by features directly related to Pokémon, such as catching all possible Pokémon and reaching higher levels, whereas former users stress the importance of general game quality, such as better augmented reality and more challenges in the game | Player demographic and reason to play PoGo | Not applicable |
| Rauschnabel et al. [135] | 2017 | Germany | Quantitative survey |  | Pokémon Go players | Not applicable | Assessed Enjoyment, Activity, Flow, Nostalgia, Social Norms, Socialising, Image, Privacy Risks, Physical Risks, Attitude toward using, Intention to continue playing Pokémon Go, Intention for In-app purchases | Drivers of attitudinal and intentional reactions | Consumers' attitudes toward playing mobile AR games are mostly driven by the level of enjoyment they receive and the image that playing a particular game conveys to other people. In addition, nostalgia, the flow experience, and the physical activity from playing contribute to a positive association. Socializing is not related to any of the target variables. In-app purchases are driven by flow, image, and social norms, while attitudes toward playing the game are not a driver of in-app purchases.  Hedonic, emotional, and social benefits and social norms drive consumer reactions while physical risks (but not data privacy risks) hinder consumer reactions. However, the importance of these drivers differs depending on the form of user behavior. | Factors affecting intention to play and spend in PoGo (motivation) | Not applicable |
| Richards et al. [136] | 2018 | United Kingdom | Case report |  | Pokémon Go player | Not applicable | Not applicable | Not applicable | Fit and well young man sustained high-voltage electrical burns from falling onto a railway track while playing Pokémon Go. Limb salvage was not possible in this patient, and therefore, in addition to his acute care, the disease burden will include the long-term costs associated with an above knee amputation in a young man.  The use of mobile phones causes both auditory and visual distraction, and this should be considered when developing measures to reorientate users to hazardous situations. | Risky behaviours of PoGo players | Not applicable |
| Richardson et al. [137] | 2022 | Spain | Interview | Late 2018–early 2019 | Older adults playing Pokémon GO | Not applicable | Three ethnographic techniques – scenarios of use, in-depth interviews and re-enactments | Participants’ experience through a a reflexive narrative of the gameplay; social and embodied dimensions of play | Mobile location-based and AR games present a spectrum of affordances: hybrid modalities of experience (through the coalescence of digital, networked and physical ways of knowing); digital wayfaring (as this hybrid experience generates a new kind of collective place-making); haptic play (through the tactile intimacy of the touchscreen and ‘feel’ of the game); ambient play (as the game becomes diffused through the embodied routines of everyday life) and social play (via the embedment of collaborative action in the game’s mechanics).  It is mundane media interfaces and practices – audio and video calls on computers and phones, casual network play through online games – that allows us to support social inclusion and re-enact tacit forms of informal care, for friends, families and the community. | Social benefits of PoGo | Not applicable |
| Rowntree & Feeney [138] | 2019 | Ireland | Quantitative survey |  | Outpatients attending an Irish general adult mental health service | Not applicable | Opportunistic, anonymised, cross-sectional survey of outpatients attending an Irish general adult mental health service. Asked about smartphone ownership, games played on smartphones, and those played using other platforms, Pokémon GO knowledge and gameplay | Perceived effects of smartphone and video games in an Irish community mental health service population | The two individuals who found Pokémon GO usage increased their exercise levels, also reported mental health benefits from it. Individuals’ gaming use and age did not significantly impact on whether they were positive or negative in their opinions towards video and smartphone games.  As more frequent users, perhaps younger individuals would most benefit from gamification of interventions and the use of existing games that have possible physical and mental health benefits. | Mental and physical health impacts of playing PoGo | Self-report questionnaire |
| Ruiz-Ariza et al. [139] | 2018 | Spain | Randomized controlled trial | Second week of August 2016 and after 8 weeks (third week of October 2016) | Adolescents from two summer schools from Andalusia (Spain) | Playing Pokémon GO for 8 weeks | Ad hoc 1 min memory test, Trait and Emotional Intelligence Questionnaire Short Form | Cognitive performance (CP) and emotional intelligence (EI) | Players walked 54 km and spent 40 min/day playing in this period. Boys played more, won more points and reached a higher level in the game than girls. The players playing Pokémon GO significantly increased their selective attention, concentration levels, and sociability levels against their peers.   It is concluded that Pokémon GO increases, in a playful way, the amount of daily exercise in adolescents, could positively affect their cognitive performance, and improve the social relationships. | Physical activity, social, and cognitive benefits of PoGo | Self-report questionnaire |
| Sawano et al. [140] | 2017 | Japan | Case report |  |  | Not applicable | Not applicable | Not applicable | A 33-year-old Japanese man was run over and dragged on the road for ~50 m. Despite an immediate medical attention, he died of multiple trauma and hemorrhagic shock after 2 h. The driver fled the accident site, but was caught and arrested 2 days later. Detailed investigation revealed that he was playing Pokémon GO when he caused the accident. | Risk of playing PoGo | Not applicable |
| Schade et al. [141] | 2020 | United States | Randomized controlled trial | November 28, 2016 to February 8, 2017 | Healthy undergraduate students | Randomly assigned to either Pokémon Go-playing group or control group | Fitbit Charge Heart Rate FB405BKL monitors were applied to determine step count, minutes of physical activity, caloric expenditure, and resting heart rate | Daily number of steps and distance travelled | No statistically significant difference in means was observed when comparing the average daily step count (p = 0.845) or the average daily distance travelled (p = 0.528) between control and PoGo-playing participants. | Physical activity impact of PoGo | Fitbit Charge Heart Rate devices |
| Serino et al. [142] | 2016 | International | Commentary |  |  | Not applicable | Not applicable | Not applicable | Potential concerning dangers of playing Pokémon Go include physical harm from distracted walking or driving, as well as harmful interactions with potential predators who may use the game to attract children to isolated locations.  Pokémon Go has the potential to be an exciting new way for parents to spend time with their children and share in their interests. In addition, when used in a proper and appropriate setting, it may give children the confidence to explore their surroundings, develop relationships, and engage in physical activity. However, it is imperative that rules and guidelines be clearly defined to ensure the continuing safety and wellbeing of all children. | Risk of playing PoGo | Not applicable |
| Shen [143] | 2019 | Taiwan | Interview and quantitative survey |  | Gamers and individuals with professional expertise | Not applicable | Interviewed 9 experts, collected 235 Kansei words from 33 articles, and surveyed 335 gamers through a questionnaire to collect the data about users’ preferences. Evaluation Grid Method (EGM), as a qualitative method, is used for deep interviews. | Motivated reasons for the appeal of mobility-augmented reality games | The strongest two original evaluation items of Pokémon GO are determined as “social interaction” and “scenario interaction” based on the statistical analysis of Quantification Theory Type I.  The popularity of Pokémon GO can be ascribed to the design of the innovative models of game interaction, which targets the psychological preferences of gamers and successfully drove gamers’ motivations to play Pokémon GO. | Successful PoGo game features (motivation) | Not applicable |
| Shiau & Huang [144] | 2023 | Taiwan | Quantitative survey | 2020 | Pokémon Go users | Not applicable | Developed reliable self-report scales for cognitive and emotional fit | User satisfaction and continued intention to play | In the Stimulus-Organism-Response framework, information quality, system quality and virtual (Pokémon) characteristics are the main factors that influence cognitive fit and emotional fit. The main factors that influence user satisfaction are cognitive and emotional fit. Finally, continued intention to play is an essential factor that is influenced by satisfaction.   The intersection between virtual world and reality,that is, smoothness and authenticity of game graphics, allows people to relate to the game, whether the game is the city they live in, their life or their surrounding environment. For this reason, players will invest more energy in their relationship with their Pokémon. | Successful PoGo game features (motivation) | Not applicable |
| Smith et al. [145] | 2021 | International | Qualitative survey | 2 February – 1 March 2020 inclusive | PoGo or HPWU players | Not applicable | Qualitatively mapping both psychographic (constraints, involvement, loyalty) and behavioral (player typologies and player status) data to profile groups of players, and their pattern of play (hardcore, in-between, casual) | Better design for location-based augmented reality exergames to improve exercise and health | Three themes were identified: 1) player loyalty, 2) player involvement and 3) player constraints. Psychographic constraints experienced can negatively influence player preference and behavior.  However, psychographic enablers such as player involvement with the franchise and player loyalty toward the brand can act to sustain continued gameplay across different player types and should not be underestimated as a powerful influence in decision-making, choice behavior, and behavior change (to improve exercise and health). | Successful PoGo game features (motivation) | Not applicable |
| Sobel et al. [146] | 2017 | United States | Interview and qualitative survey |  | Adult guardians who allowed their children to play Pokémon GO | Not applicable | Qualitative analysis of survey data and semi-structured interview questions | Coded survey data and interviews | While parents still had some typical concerns about screen time, the fact that the game motivates outdoor, social, and bonding activities helped mitigate these worries. Parents could alleviate new concerns about safety by accompanying and playing with their children.  Perhaps it was Pokémon itself that initially on-boarded families to play, but the valuable experiences of and interactions within families existed and continued to exist beyond the actual content of the game. Without these other important elements, we argue the game’s popularity for families would not have been sustained. | Social benefits of PoGo and risk | Not applicable |
| Sun [147] | 2024 | United States | Randomized controlled trial | April 2023 until June 2024 | Healthy, physically inactive adults | A randomized controlled trial to test whether pairing an exergame with a standard cognitive-behavioral PA promotion intervention improves PA beyond that of the cognitive-behavioral intervention alone. | Participants completed a demographic measure at baseline, and measures of other constructs at baseline and the end of each week of the intervention. All participants also completed an additional weekly self-report survey. | Physical activity, Intrinsic motivation for PA, Acceptability of the intervention | No significant difference in steps at Week 4 between the two conditions, intrinsic motivation for PA did not mediate the relationship between condition and post-treatment PA, and none of the moderators significantly interacted with the exergame to affect post-treatment steps, though there were main effects of area walkability, liking for video games, and attitudes toward video games. Pokémon GO demonstrated mixed acceptability.  The complex gameplay decreased enjoyment of the game, likely because participants failed to experience a sense of competence, which is key to intrinsic motivation. Thus, the game failed to fulfill the basic purpose of an exergame – to be fun, enjoyable, and motivating to play. | Motivation to play PoGo and physical activity impact | Fitbit tracker |
| Tabacchi et al. [148] | 2017 | Italy | Quantitative survey | 1st and the 31st of August, 2016 | Pokémon Go (PoGo) players | Not applicable | Developed and deployed the Pokemon Go Study Questionnaire (PoGoStQ), which queries PoGo players about qualitative and quantitative usage, as well as their Big Five personality traits. | Specific differences in personality traits of early PoGo adopters; correlations between PoGo usage and personality traits; comparing personality traits | The resulting profile of early PoGo player is one of a more Introverted, close person with high agreeability and conscientiousness. Extraversion and Stability are positively correlated with the collection part of the game, while Agreeableness is a negative predictor thereof. Openness is correlated to the level of proficiency.  Personality profile of early PoGo users is more assimilable to video-gamers than to SN or mobile users. | Personality traits and motives of PoGo players | Not applicable |
| Tang [149] | 2017 | International | Quantitative survey | One year after the first survey and 3 weeks after the official release of Pokémon GO, an additional survey was conducted focusing on the players of Pokémon GO | Players of Pokémon GO | Not applicable | Theoretical framework grounded on telepresence theory and social capital theory | Success factors of Pokémon GO | Success is due to its perfect match, in that it allows players to fulfill their childhood fantasies in reality through the telepresence medium. The role-playing in the game further creates another level of interactivity with cooperation and competition among gamers. Afterward, normative influence is formed when a huge number of people are interacting in the game, drawing in non-fans to play.  Pokémon GO universe is consistent with the telepresence medium concept espoused in telepresence theory as well as the social capital formulation, in that a process is evoked through the cognitive, structural, and relational dimensions. | Successful PoGo game features (motivation) | Not applicable |
| Tannemaat & Aziz [150] | 2017 | International | Editorial |  |  | Not applicable | Not applicable | Not applicable | One method to ‘‘nudge” large numbers of people towards a desired behaviour could be through the application of Augmented Reality (AR) games. Movement to and from infection zones could thus be actively discouraged on a real-time basis, resulting in ‘‘dynamic virtual quarantines” and reduced rates of disease transmission.  The effect of such dynamic virtual quarantines would likely be small. Nonetheless, they could help mitigate the disease-transmitting properties of the Pokémon network during disease outbreaks. | Societal impacts of PoGo | Not applicable |
| Tateno et al. [151] | 2016 | Japan | Commentary |  |  | Not applicable | Not applicable | Not applicable | On the one hand, there have been numerous internet reports of Pokémon Go gamers sustaining injuries as they have ventured into their surroundings in a distracted state, but on the other hand, there are also reports of gamers becoming less sedentary and having improvements in depression and anxiety through promoted physical activity.  Pokémon Go is designed to be user-friendly, such that with a simple flick of a finger, even mobile game novices can collect balls and throw them to catch Pokémon on the screen. Setting up PokeStops at hikikomori support centers may attract individuals with hikikomori to catch Pokémon and communicate with others. | Mental and physical health impacts of playing PoGo and risk | Not applicable |
| Thompson [152] | 2016 | United States | News article |  |  | Not applicable | Not applicable | Not applicable | At least 14 crashes were attributed to Pokemon Go during a 10-day period in July. A random sample of 4,000 tweets collected during 10 days in July showed that many players are relying on motor vehicles to help them locate the creatures. A full third of the tweets indicated that a driver, passenger or a pedestrian was distracted by Pokemon Go, which correlates to nearly 114,000 incidences reported on Twitter in 10 days. | Risky behaviours of PoGo players | Not applicable |
| Thompson [153] | 2017 | United States | News article | June 15 to July 31, 2016 | iPhone users that had played the game since its July 2016 launch | Not applicable | Not applicable | Not applicable | Pokemon Go players were twice as likely to walk 10,000 steps a day than they were before taking up the game, researchers reported. Overweight or sedentary people appeared to benefit most from the game. | Player demographic and physical activity benefits | Not applicable |
| Thongmak [154] | 2024 | Thailand | Quantitative survey | From 2019 | Pokémon Go players in Thailand | Not applicable | Measurement model using survey questionnaire results. | Continuance intention, Intention to recommend, Intention to use other LBSs | The insignificant relationship between satisfaction and continuance intention on game addiction could be explained by the low mean of Pokémon Go players’ game addiction.  The insignificant effect of functional value on gamers’ continuance intention could be supported by the negative impact of the perceived functional value of a game item on purchase intention. | Understanding continuing/discontinuing PoGo | Not applicable |
| Thongmak [155] | 2020 | Thailand | Quantitative survey | From 2019 | Individuals who currently played or did not play Pokémon Go | Not applicable | The questionnaire has a section exploring players' and non-players’ perceptions, a section investigating a respondent's behavioural intention, and a section about participants' demographic characteristics, their perceived innovativeness, the frequency of using mobile Internet/playing games, and game preferences. | Motives, social capital, loneliness, life satisfaction, physical health, game perceptions | Game aesthetics increase all perceived values of both groups. Game aesthetics and innovativeness have no direct impact on gamers' intention to play. Emotional value and functional value are crucial for their behavioural intention. Social value is important for non-players, while conditional value influences players' intentions.  All players' perceptions are higher than non-players’ perceptions. The positive impact of game aesthetics on functional value is stronger for non-players than for players. | Factors affecting intention to play PoGo | Not applicable |
| Thongmak [156] | 2022 | Thailand | Quantitative survey | From 2019 | Pokémon GO players, full-time students and full-time employees | Not applicable | Questionnaire investigating self-efficacy to protect information privacy, privacy knowledge, privacy concerns, and privacy protection behaviour, and the frequency of playing Pokémon GO, and game preferences. | Direct or indirect effects of self-efficacy to protect information privacy, privacy knowledge, privacy concerns, and perceived risks on privacy protection behaviours among players | Privacy knowledge, self-efficacy, privacy concerns, and perceived risks are confirmed as salient factors directly or indirectly influencing the privacy protection behaviour of players one way or another.  Findings in this study reveal some irrational behaviours of users, for instance, students’ having high privacy concerns but low protection behaviours. | Risk of playing PoGo | Not applicable |
| Thongmak [157] | 2019 | Thailand | Conference paper, quantitative survey |  | Full-time students and full-time employees as non-adopters | Not applicable | Survey based on a research model including 6 factors: perceived enjoyment, subjective norms, privacy concerns, perceived ease of use, and perceived usefulness, and intention to play Pokemon Go | Intention to play the game for non-adopters. | Intention to play of non-players is directly enhanced by perceived enjoyment, perceived usefulness, and subjective norms. Privacy concerns indirectly influence intention to play via perceived usefulness. Subjective norms affect perceived ease of use, perceived enjoyment, and perceived usefulness respectively. Enjoyment and subjective norms also have indirect impacts on playing intention. Findings yield the contrary results in the context of Pokemon Go in Thailand. | Factors affecting intention to play PoGo | Not applicable |
| Tong et al. [158] | 2017 | Not mentioned | Conference paper, quantitative survey |  | Pokémon Go players | Not applicable | Online questionnaire exploring Pokémon Go players’ experience was designed to figure out their motivations and play patterns. | Motivation to play, reason to stop, game mechanics they like and dislike | Pokémon Go benefits its players with increased exercise, social connectivity, and outdoor activity. However, players hold negative attitudes towards safety and privacy concerns, time commitment, and its game mechanics design.   Ironically, on the one hand, players’ PA can be increased through playing the game; on the other hand, they spend the same amount of time looking at their phones instead of the real environment. | Risk, physical activity, and social impacts of PoGo | Self-report questionnaire |
| Tong et al. [159] | 2017 | Not mentioned | Quantitative survey | September to October 2016 | Pokémon Go players | Not applicable | Questions regarding When, Where, and with Whom were asked. Other questions included: participants’ self-reported PA changes, connections with others, chances meeting new people, and time holding cell phones on a 100 Visual Analog Scale. At the end, one open question was asked: what motivates/impresses you most when playing this game? | Motivation to play, reason to stop, game mechanics they like and dislike | Free-to-play, location-based AR mobile games like Pokémon Go are likely to become a new design model for gamified applications that promote physical activity.  In order to sustain motivation and physical activity, the core gameplay and mechanics require thoughtful and engaging design. | Successful PoGo game features (motivation) | Not applicable |
| Urwin & Flick [160] | 2019 | International | Quantitative and qualitative survey | 3 months following the launch of Pokémon Go | Active Pokémon Go players | Not applicable | Mix of open and closed questions.   Closed questions such as how long the individual has been playing Pokémon Go for, how often they play, and what situations they play in. Open questions on experience and mood, asking what players enjoy about the game, what they dislike, how they feel after catching a Pokémon, if these feelings last, and if they have noticed and impact upon other areas of their lives. | Experiences and associated mood changes (if any) of Pokémon Go players | Respondents to the survey viewed playing the game overwhelmingly positively, and were very open with the positive impacts they had experienced due to playing. Respondents mentioned speaking to friends or family more regularly than they had previously.  The benefits Pokémon Go have for mood are strongly linked to the game mechanics, and the use of gamification. By utilising positive reinforcement for in-game activities through rewards, bonuses, or trophies, the benefits to mood are more likely to be maintained as gameplay is maintained. | Successful PoGo game features (mental well-being) | Not applicable |
| Vaterlaus et al. [161] | 2019 | United States | Qualitative survey | 2018 | College students who currently played, no longer played, or had never played Pokémon Go | Not applicable | In the online survey, Pokémon Go users and nonusers were directed to different question tracks with open-ended items | Perceived behavioral outcomes of playing between current and former players | Three themes were identified: (a) limitations to playing Pokémon Go, (b) motivations for playing Pokémon Go, and (c) social consequences of playing Pokémon Go.  Taken together, college-aged Pokémon Go users and past users in this study were motivated to play to gratify their coolness, novelty, nostalgia, realism, community building, activity, health/exercise behaviors, and play/fun needs. Nonusers and past users reported the failed gratifications of novelty, coolness, nostalgia, community building, play/fun, functionality of app with device, and safety. | Motivation to play PoGo | Not applicable |
| Vella et al. [162] | 2019 | Australia | Interview and internet user analytics | 2016 | Pokémon Go (PoGo) players seen standing close to PokéStops | Not applicable | Semistructured interviews were conducted and when necessary, follow-up questions were asked for greater clarification and understanding. Broadly, the questions probed players’ social play experiences | Specific aspects of the game or gameplay that promote the social outcomes and how PoGo facilitate social connectedness | Playing PoGo produced a sense of belonging, linked to a sense of place, as well as facilitating conversations with strangers and strengthening social ties. This was due to the use of accessible technology able to be integrated into daily routines, shared passion for the game, and mechanics that encouraged players out of their homes.   “Shared passion” was tied to the nostalgic connection many players felt for the franchise. This study shows how gameplay can build social connectedness through real-world engagement. | Successful PoGo game features (motivation) | Not applicable |
| Vincent [163] | 2016 | Australia | News article |  |  | Not applicable | Not applicable | Not applicable | A police station in Australia is warning against actually walking into the station just to gain in-game items.  Paying attention to your surroundings is good advice when it comes to playing games or just using a smartphone in general, but unique to Pokémon Go is the serious battery drain it's causing for users | Risk of playing PoGo | Not applicable |
| Wagner-Greene et al. [164] | 2017 | United States | Editorial |  | Adult players playing the game during the last two weeks of July 2016 | Not applicable | Convenience sample survey | Play while driving, Play while riding a bicycle, hoverboard, etc., Pay more attention to Pokémon GO than to your surroundings while walking, Play in areas where you do not feel safe, Enter private property, Sacrifice sleep | These games may be an opportunity for the field of public health to use novel approaches to combat epidemics such as obesity. However, the negative aspects are potentially life threatening. Health communication messaging and other strategies will need to be developed to expand on the traditional work aimed at reducing texting and driving or distracted driving. | Risk of playing PoGo | Not applicable |
| Wang & Skjervold [165] | 2021 | International | Quantitative survey | September 9th to October 20th, 2016 | Pokémon Go players | Not applicable | Goal, Question Metrics (GQM) approach; first define a research goal (conceptual level), then define a set of research questions (operational level), and finally describe a set of metrics to answer the defined research questions (quantitative level).  47 questions about demographics, gaming habits, physical activity and health, social activity and health, and adverse behavior. Sixteen questions were open-ended. | Physical and social activity effect of Pokémon Go | Playing Pokémon Go has a statistically significant positive effect on physical and social activity. It was also found that the game had a different effect on various groups of players and that 50% of the players reported positive health benefits, including weight loss, loss in body fat, and gain in muscle mass.   Most significant result is how Pokémon Go managed to motivate groups who are hard to motivate to be physically and socially active. The game had a more substantial positive physical effect on players who were initially less physically active, male, unemployed, gamers than casual and non-players and played the game in denser populated areas. | Player demographics and physical and social activity benefits | Self-report questionnaire |
| Wang & Wu [166] | 2020 | International | Quantitative survey | Summer of 2016 | Respondents’ ages range from 18 to 60. The majority of the respondents were Whites (81.8%, n = 224), followed by Asians (10.2%, n = 28).  Respondents spent an average of 52 minutes playing Pokémon GO daily. | Not applicable | Use of Pokémon GO game features was measured by asking respondents to evaluate their frequency of using various game features on a seven-point Likert scale.  Sense of community was measured by nine items adopted from Peterson, Spear, and McMillan (2008) on a seven-point Likert scale.  Sense of companionship was measured by 25 items on a seven-point Likert scale adopted from the companionship for the artificial pets scale. | Sense of community, sense of  companionship, and psychological well-being | Results showed frequency of collecting Pokémon, customizing Pokémon,  performing gym related activities, purchasing special items, and taking  pictures with the Pokémon led to increased sense of community, sense of  companionship, and psychological well-being.   Frequency of performing gym-related activities, purchasing special items, customizing Pokémon, and taking pictures of Pokémon positively predicted sense of companionship. Collecting Pokémon can offer Pokémon GO players a sense of community, while customizing Pokémon can offer Pokémon GO players a sense of companionship. Sense of community and sense of companionship are two precursors of psychological well-being.  In addition, sense of community and sense of companionship mediated the relationship between frequency of using these five Pokémon GO features and psychological well-being respectively. | Social and psychological benefits of PoGo (successful features) | Not applicable |
| Wang et al. [167] | 2022 | United States | Quantitative survey; longitudinal analysis | Sep 2016 to Sep 2017 | Mother-child dyads currently playing Pokémon GO | Not applicable | Questionnaire, accelerometer  Differences in moderate to vigorous PA (MVPA) over time among individuals playing Pokémon GO compared to non-players | MVPA | Children’s daily MVPA did not differ by player status, but mothers who reported playing engaged in higher daily MVPA (M = 46.84, SD = 38.07) compared to non-players (M = 21.40, SD = 23.31).   This naturalistic study lacked power to further analyze changes in MVPA after the release of the game due to lack of engagement with Pokémon GO | Physical activity impacts of PoGo | Accelerometer and self-report questionnaire |
| Watanabe et al. [168] | 2017 | Japan | Quantitative survey | Baseline (Nov 26, 2015–Feb 18, 2016) and at follow-up (Dec 1–4, 2016) | Workers in Japan | Not applicable | All variables were measured using the online self-report questionnaire that developed by the authors, including Brief Job Stress Questionnaire (BJSQ), WHO Health and Work Performance Questionnaire (WHO-HPQ) | Physical complaints, work performance, psychological distress | Improvement in psychological distress was significantly greater among Pokémon GO players than among non-players. Pokémon GO may be effective for improving psychological distress among workers. Although its effect size is small, the game could have positive effects on the mental health of the adult working population. | Mental health impacts of playing PoGo | Not applicable |
| Wattanapisit et al. [169] | 2018 | Thailand | Quantitative survey; longitudinal analysis | 2016 | Medical students, who played Pokémon GO | Not applicable | Demographics, patterns of playing Pokémon GO (time spent gaming, mode of commuting while playing game, reasons for playing Pokémon GO and game-related injuries) and information on PA and sedentary time using the Global Physical Activity Questionnaire (GPAQ) version 2 (Thai version) | Changes in physical activity | The key reasons for playing game were `have fun' and `pass time/boredom'. The most common commuting mode to play the game was walking; some drove a car or motorcycle while playing the game. There was no correlation between physical activity and time spent gaming.   This study highlights how the lack of sustainability of the game and the motivation behind using Pokémon GO as a game rather than a physical activity app may have undermined the potential of using the game to improve physical activity. | Physical activity impacts of PoGo | Self-report questionnaire |
| Wedell [170] | 2016 | International | News article |  |  | Not applicable | Not applicable | Not applicable | Previously, Pokémon GO account creation process on iOS erroneously requests full access permission for the user’s Google account. Pokémon Go creator Niantic has since pushed through an iOS app update that corrects the full access error.  “*It’s kind of an invasion of privacy but as long as they don’t give my information out to anybody I don’t see an issue with it,*” said Griffin Goetz, 18, of Centerville. | Risk of playing PoGo | Not applicable |
| Wei & Wang [171] | 2019 | United States | Conference paper, quantitative survey | 2 months after the release of the game in the U.S. | Owned a smartphone and played Pokémon GO | Not applicable | Questionnaire assessing history of playing Pokémon Go, exercising during Pokémon Go play, short and long-term likelihood of engaging in exercises, and McCroskey’s willingness to communicate (WTC) scale | Willingness to communicate, and the likelihood of engaging in exercises | The longer participants had been playing the game, the higher the likelihood that they would engage in exercises. Findings revealed a positive relationship between exercise during gameplay and willingness to communicate with other players. | Physical activity impacts and social benefits of PoGo | Self-report questionnaire |
| Williams & Slak‑Valek [172] | 2019 | International | Quantitative survey | September 18, 2016 to 20 December 2016 | Pokémon GO players | Not applicable | 7 items measuring travel changes, 6 items measuring physical activity changes, 6 items measuring social life changes and 3 items measuring hours of play, the mood after play and the feeling of success after play. | Travel attitudes and behaviors, physical activity and the social life of players | Playing the networked game on a mobile device increases visitation to local tourism attractions, and motivates people to spend more time outdoors and walking. It also sends them to cities and countries they might not otherwise visit and acts as an alternative to spending time indoors playing computer games. Surveyed players feel happier and more successful after playing the game. Pokémon GO might have value as the center of a tourism product for those players. The opportunity to acquire Pokémon and encounter others involved in the game across a destination, or in a single attraction, could pull players to that place. | Societal impacts of PoGo | Not applicable |
| Winand et al. [173] | 2022 | Singapore | Interview | February and April 2017 | Young adults residing in Singapore, players and ex-players | Not applicable | Interview questions aimed to understand motivational factors for players to play the game and be active, possible reasons why they were more active when they play Pokémon Go and if MAR gaming has the potential of sustaining increased physical activity levels in young adults. | Physical activity sustainability and motivational factors | Findings revealed players were motivated to play to fulfil their needs for competency and relatedness. While the game seemed to impact players’ physical activity behaviour, it was not sustained.   Already physically active participants may have reduced their physical intensity level when playing. In addition to eventually reducing their physical activity level, players also circumvented the need to be active to play by adopting deviant behaviour to achieve game goals and their needs for competency. | Motivation to play PoGo and physical activity impact | Self-report questionnaire |
| Wingenbach & Zana [174] | 2022 | Brazil | Observational | April to August 2019 | Pokémon Go players | Not applicable | Survey questions | Demographic characteristics, mental health, game-specific questions, direct judgement on the effect of the game on participants, and questions on life satisfaction and social functioning | Higher self-reported social functioning and life satisfaction since playing PoGo compared to the time period before playing, which involved a shift from negative to positive ratings. The increases in self-reported life satisfaction and sociality (but not social ability) were more pronounced for the clinical compared to the non-clinical subsample  The effect of the social ability change on the life satisfaction change was mediated by the sociality change and moderated by the number of daily in-person player interactions (including strangers)  Findings using subjective judgements show that PoGo motivates social interactions and increases life satisfaction | Player demographic and well-being impact | Not applicable |
| Wong [175] | 2017 | Hong Kong | Quantitative survey | 28 days after the game was released (2016) | University students | Not applicable | International Physical Activity Questionnaire (IPAQ)—short form, | Physical activity levels | Compared with the ex-players, the players were significantly more frequent to stay outdoors when playing Pokémon Go, walk/jog to a location to catch Pokémon, to Pokéstops or Gyms as well as walking/jogging to hatch eggs. However, there was no significant difference in physical activity levels between the three groups.  Players who used to be sedentary benefited the most from Pokémon Go. The game can be used as a starting point for sedentary people to begin an active lifestyle. | Physical activity impacts of PoGo | Self-report questionnaire |
| Wong et al. [176] | 2020 | Hong Kong | Interrupted time-series design | From January 1, 2012 to July 31, 2018 and from January 1, 2002 to July 31, 2008 | Accident and emergency attendances attributed to self-harm, other intentional injuries and minor noninjuries | Not applicable | Interrupted time-series analysis with a slope change model | Effects of Pokémon Go launch on the trends of accident and emergency attendances | Self-harm incidence in the population, particularly in adults, showed a significant decline in the period after Pokémon Go was launched. Augmented reality games such as Pokémon Go show great promise as a tool to enhance psychosocial well-being and improve mental health. | Mental health impacts of playing PoGo | Not applicable |
| Wong et al. [177] | 2017 | Australia | Observational | July 2016 and March 2017 | Two authors | Not applicable | The authors played the game when they felt like they would like to, just like normal gamers and discussed the game with their friends and colleagues. With their IT design knowledge and health knowledge, the authors documented features within Pokémon Go on a computer spreadsheet and that might encourage lifestyle changes. | Authors' game experience | This paper suggests from the perspective of a user on changes to the game that potentially could help with obesity, mental health cardiovascular health and vitamin D deficiencies. Rather than info-centric monitoring, measurement or advice based approaches, it seems that ‘gamifying’ interactions and advice through fun may be an additional approach to deliver exercise health benefits.While the impact of augmented reality games on improving exercises might be substantial, the question of sustainability and likely long-term health outcomes remain debatable.   The rapid uptake of Pokémon Go by the population around the world, however, should serve as a useful lesson for information and technological design to improve outcomes of obesity-related diseases in the future. | Successful PoGo game features (physical health) | Not applicable |
| Wragg & Whall [178] | 2024 | England | Observational |  | Fathers and their two boys | Autoethnographic data were collected over a period of 6 months of play between the authors and their sons. | Through collaborative play, and discussions between the parents and their two boys, aged five and thirteen, insight was gained into how Pokémon Go can facilitate active movement, local environment awareness, and inter-social skill development. | Autoethnographic data of play between the authors and their sons | Given the transformative influence of mobile technologies on traditional outdoor play, Pokémon Go can promote physical activity, facilitate enskilment, and foster a greater appreciation for the player’s actual physical environment. By providing users with an interactive and immersive experience, Pokémon Go encourages players to explore their local surroundings and seek out points of interest.  The game’s social aspect can foster intergenerational and intercultural interactions by connecting people through shared interests, promoting family bonding, facilitating cultural exploration, encouraging social cooperation, providing learning opportunities, promoting global inclusivity, thereby ultimately enhancing players’ social skills. The game’s location-based mechanics inspire exploration, bridging generational gaps and fostering cross-cultural understanding.  The extrinsic motivation of chasing the arbitrary 10,000 steps a day pales in insignificance to the intrinsic drive to play for the joy of playing, with the walking and movement all just part of the gameplay. | Successful PoGo game features (physical health, cognition) | Not applicable |
| Wu et al. [179] | 2021 | Taiwan | Quantitative survey |  | Experienced Pokémon Go players | Not applicable | Questionnaire assessing the four identified critical constructs (i.e., context facilitation, intrinsic motivation, situational engagement, and player value) | Pokémon Go–derived player value | Context facilitation and intrinsic motivation have direct effects on situational engagement; situational engagement has a direct effect on player value; and situational engagement partially mediates the effects of context facilitation and intrinsic motivation on player value.   Intrinsic motivation and context facilitation are the most critical factors in collectively triggering players’ situational engagement and thereby creating player value in the cyber–physical environment. Motivations such as levelling up and collecting rare game items are just as important at engaging players as interacting within a cyber-physical environment. | Successful PoGo game features (motivation) | Not applicable |
| Wulf & Baldwin [180] | 2020 | Study 1: Germany Study 2: United States | Quantitative survey | Study 1: July 2016 before game release  Study 2: August 2016 after game release | Study 1: German participants Study 2: Installed and played the game Pokémon Go | Not applicable | Study 1: Intention to play the game as well as how nostalgic they feel, life experiences with Pokémon  Study 2: Factual data of their game-play, entertainment experiences and how nostalgic they feel, how meaningful Pokémon was to them | Study 1: Anticipated nostalgia, intention to play  Study 2: Psychological well-being and subjective well-being | Study 1: The meaning players ascribe to Pokémon predicted anticipated nostalgia, which in turn motivated them to play the game.  Study 2: Replicates and extends Study 1's findings in an American sample after the game’s release. Again, meaningfulness of Pokémon predicted game-induced nostalgia, which mediated the effect of meaningfulness on eudaimonic entertainment. Eudaimonic entertainment, in turn, predicted psychological well-being. Taken together, these findings draw a bi-directional link between nostalgia and media consumption and substantiate research regarding the well-being functions of both. | Motivation to play PoGo | Not applicable |
| Xian et al. [181] | 2017 | United States | Observational | June 15 and July 31, 2016 | Pokémon GO players | Not applicable | Participants were instructed to provide screenshots of their step counts recorded by the iPhone Health app and their “Pokémon GO Trainer Profile” between June 15, 2016, and July 31, 2016 | Step count | Pokémon GO participation was associated with a significant increase in PA among young adults. On average, 10 000 “XP” points (a measure of game progression) was associated with 2134 additional steps per day, suggesting a potential dose-response relationship.  Increased PA was also observed in subgroups, with the largest increases seen in participants who spent more time playing Pokémon GO, those who were overweight/obese, or those with a lower baseline PA level. Incorporating PA into gameplay may provide an alternative way to promote PA in persons who are attracted to the game. | Physical activity impacts of PoGo and player demographics | Wearable/Phone |
| Yan et al. [182] | 2020 | United States | Quantitative survey | 2016 | Non-users, non-active users, and active-users | Not applicable | An online survey was developed by the research team to measure participants’ Pokémon Go related behaviour and perceptions, as well as their physical activity level. | Physical activity participation, motivation, enjoyment, and confidence | Most users agreed that playing the game had a positive impact on their physical activity level. However, the non-active users and the active-users walked significantly less than the non-users did, and the non-users also had higher overall physical activity participation levels.   Most Pokémon GO players perceived that the app made them more physically active, although they still walked less than non-players did. | Physical activity impact of PoGo | Self-report questionnaire |
| Yang & Liu [183] | 2017 | United States | Quantitative survey |  | Pokémon Go players | Not applicable | Pokémon Go Motive Scale, social capital scale, UCLA Loneliness Scale, Satisfaction with Life Scale, Physical and Environmental Health Subscale of the Holistic Health Score Sheet | Implications of gaming for players’ psychosocial and physical well-being | Exercise, Fun, Escapism, Nostalgia, Friendship Maintenance, Relationship Initiation, and Achievement. Both Fun and Friendship Maintenance were positive correlates of well-being, whereas Escapism and Nostalgia were negative correlates. Relationship Initiation was associated with both better and poorer well-being. | Motivation to play PoGo and well-being impact | Not applicable |
| Yee et al. [184] | 2017 | Australia | Observational | July to August in 2016 | Two of the authors approximately 100 other players | Not applicable | Personal participatory field notes as well as observational field notes were obtained regarding using the technology and human behavior and observations during observation. | Health behavioural changes in the community | The method of stimulating these changes behaviours through entertainment and fun appear to be able to achieve, at least in the short term, patterns of behavior that lots of ‘info-centric’ client focused health technologies, applications and services have failed to achieve.   Pokémon Go appears to present a change in the relationship between lifestyle advice and end-users. In Pokémon Go the lifestyle advice is no longer a direct objective of the application. The main interaction is between the user and the technology as part of an entertainment or game that has embedded within it lifestyle modification of behaviors. The real test is sustainability. | Successful PoGo game features (physical health) | Not applicable |
| Yip et al. [185] | 2023 | Hong Kong | Interview | Between July 2017 and July 2018 | Young adults playing Pokémon GO | Not applicable | Focus group questions | Factors in social, individual, interpersonal, and environmental domains relate to the insights of young people’s experiences with Pokémon GO, their motivations for playing, and their perspectives on the game’s implications for the public health of their communities | Five themes emerged: 1) missing out or self-regulation, 2) childhood memories of Pokémon, 3) extending virtual-reality exploration, 4) spending more time outdoors walking and exercising, and 5) getting together with others and social interaction.  Certain negatives were identified (i.e., addiction and behavior resulting from a loss of self-control) | Motivation to play PoGo and risks | Not applicable |
| Zsila et al. [186] | 2018 | Hungary | Quantitative survey | 2016 | Active Pokémon Go players | Not applicable | Study 1: Assessed the Pokémon Go playing activity (i.e., time spent playing, platform, social connectedness) and participants' attitude and exposure to Pokémon anime. Gaming motives were assessed using the 27-item Motives for Online Gaming Questionnaire (MOGQ) and the 15 new items that were created for the purpose of this research.  Study 2: Problematic Online Gaming Questionnaire – Short Form, UPPS-P Impulsivity Scale – Short Version | Study 1: Motivations for playing Study 2: Impulsivity and problematic gaming | Recreation, Outdoor Activity, Nostalgia, and Boredom were found to be the main motivations for players. Although the results suggest that the motivation of Pokémon Go players decreased in multiple aspects, the motives of competition and skill development as well as the need for recreation increased.  The strongest motive for Pokémon Go players was recreation, which was one of the seven motivational dimensions of the original MOGQ. In addition to the seven original factors of the MOGQ, three new factors were identified: Outdoor Activity, Nostalgia, and Boredom. | Motivation to play PoGo | Not applicable |

**References**

1. Alha K, Koskinen E, Paavilainen J, Hamari J. Why do people play location-based augmented reality games: a study on Pokémon GO. Comput Human Behav. Apr 2019;93:114-122. [doi: 10.1016/j.chb.2018.12.008]

2. Alloway TP, Carpenter R. Gotta catch ‘em all: Exploring the use of Pokémon Go to enhance cognition and affect. Psychology of Popular Media. 2021;10(2):178-186. [doi: 10.1037/ppm0000283]

3. Alomar N, Alsaleh M, Alarifi A. Behavioral consequences of Pokemon GO: The exaggerated picture. Computers in Human Behavior. 2019 Jan;90:223-45. PMID: 2018-60162-024. doi: https://dx.doi.org/10.1016/j.chb.2018.08.040.

4. Althoff T, White RW, Horvitz E. Influence of Pokémon Go on Physical Activity: Study and Implications. J Med Internet Res. Dec 6, 2016;18(12):e315. [doi: 10.2196/jmir.6759] [Medline: 27923778]

5. Arjoranta J, Kari T, Salo M. Exploring Features of the Pervasive Game Pokémon GO That Enable Behavior Change: Qualitative Study. JMIR Serious Games. May 25, 2020;8(2):e15967. [doi: 10.2196/15967] [Medline: 32449689]

6. Ashar M, Thaliath L, Sali K, et al. Correlates of excessive Pokemon Go playing among medical students. Ind Psychiatry J. 2019;28(2):301-305. [doi: 10.4103/ipj.ipj_92_18] [Medline: 33223726]

7. Ayers JW, Leas EC, Dredze M, Allem JP, Grabowski JG, Hill L. Pokémon GO-A New Distraction for Drivers and Pedestrians. JAMA Intern Med. Dec 1, 2016;176(12):1865-1866. [doi: 10.1001/jamainternmed.2016.6274] [Medline: 27635638]

8. Barbero EM, Carpenter DM, Maier J, Tseng DS. Healthcare Encounters for Pokémon Go: Risks and Benefits of Playing. Games Health J. Jun 2018;7(3):157-163. [doi: 10.1089/g4h.2017.0180]

9. Barbieri S, Vettore G, Pietrantonio V, et al. Pedestrian Inattention Blindness While Playing Pokémon Go as an Emerging Health-Risk Behavior: A Case Report. J Med Internet Res. Apr 1, 2017;19(4):e86. [doi: 10.2196/jmir.6596] [Medline: 28365563]

10. Bazina N, Bernik A, Tomičić I, editors. Social effects of augmented reality and pokémon go on player’s health and well-being in croatia. Presented at: 120th esd Conference; 2024; Zagreb, Croatia. URL: https://www.croris.hr/crosbi/publikacija/prilog-skup/901524 [Accessed 2026-07-10]

11. Beach C, Billstrom G, Anderson Steeves ET, Flynn JI, Steeves JA. The Physical Activity Patterns of Greenway Users Playing Pokémon Go: A Natural Experiment. Games Health J. Feb 2019;8(1):7-14. [doi: 10.1089/g4h.2017.0168] [Medline: 30260679]

12. Beach C, Montoye AHK, Steeves JA. Differences in Physical Activity During Walking and Two Pokémon Go Playing Styles. Games Health J. Apr 1, 2021;10(2):130-138. [doi: 10.1089/g4h.2020.0070]

13. Biel AM. Pokémon GO: A Socio-Technical Exploratory Study. Arizona State University]: Arizona State University; 2016. URL: http://libproxy1.nus.edu.sg/login?url=https://www-proquest-com.libproxy1.nus.edu.sg/dissertations-theses/pokémon-go-socio-technical-exploratory-study/docview/1860879805/se-2 [Accessed 2025-11-26]

14. Bonus JA, Peebles A, Mares ML, Sarmiento IG. Look on the Bright Side (of Media Effects): Pokémon Go as a Catalyst for Positive Life Experiences . Media Psychol. Apr 3, 2018;21(2):263-287. [doi: 10.1080/15213269.2017.1305280]

15. Brecht K. Pokémon Go and Its Effect on Depressive Symptoms, Physical Activity, and Social Connectedness [Ph.D Dissertation]. Alaska, United States: University of Alaska Anchorage; 2020.

16. Broom DR, Flint SW. Gotta Catch ‘Em All: Impact of Pokémon Go on Physical Activity, Sitting Time, and Perceptions of Physical Activity and Health at Baseline and Three-Month Follow-Up. Games Health J. Dec 2018;7(6):401-408. [doi: 10.1089/g4h.2018.0002]

17. Broom DR, Lee KY, Lam MHS, Flint SW. Gotta catch ‘em all or not enough time: Users motivations for playing Pokémon GoTM and non-users’ reasons for not installing. HPR. 2019;7(1):1. [doi: 10.4081/hpr.2019.7714]

18. Bueno S, Gallego MD, Noyes J. Uses and gratifications on augmented reality games: an examination of Pokémon GO. Appl Sci. 2020;10(5):1644. [doi: 10.3390/app10051644]

19. Burney SMA, Ejaz A, Ali SA, Siddiqui FA. Discovering the Correlation between Technology Acceptance Model and Usability. International Journal of Computer Science and Network Security. 2017;17(11):53-61. URL: https://share.google/l9qcmlx4NwCAefmgg

20. Butcher L, Tucker O, Young J. Path to discontinuance of pervasive mobile games: the case of Pokémon Go in Australia . APJML. Jun 1, 2020;33(2):584-606. [doi: 10.1108/APJML-12-2019-0710]

21. Caci B, Scrima F, Tabacchi ME, Cardaci M. The reciprocal influences among motivation, personality traits, and game habits for playing Pokémon GO. Int J Hum-Comput Interact. Aug 27, 2019;35(14):1303-1311. [doi: 10.1080/10447318.2018.1519167]

22. Chen PL, Pai CW. Smartphone gaming is associated with pedestrians’ head-turning performances: an observational study of street-crossing behaviors at uncontrolled intersection in Taipei. International Journal of Sustainable Transportation. Jan 2, 2018;12(1):12-18. [doi: 10.1080/15568318.2017.1321706]

23. Chen PL, Pai CW. Evaluating the effects of smartphone racing games and other game types on pedestrian’s risk-taking inclinations. Comput Human Behav. Nov 2018;88:78-83. [doi: 10.1016/j.chb.2018.06.022]

24. Chen PL, Saleh W, Pai CW. Pokemon gaming causes pedestrians to run a red light: An observational study of crossing behaviours at a signalised intersection in Taipei City. Transportation Research Part F: Traffic Psychology and Behaviour. May 2018;55:380-388. [doi: 10.1016/j.trf.2018.03.011]

25. Chen CS, Lu HP, Luor T. A new flow of Location Based Service mobile games: Non-stickiness on Pokémon Go. Comput Human Behav. Dec 2018;89:182-190. [doi: 10.1016/j.chb.2018.07.023]

26. Cheng R. National Steps Challenge Is Back With Limited Edition Pokémon Collectibles, Free Trackers & Prizes Worth Over $40,000. TheSmartLocal; 2019. URL: https://thesmartlocal.com/read/national-steps-challenge-2019 [Accessed 2025-11-26]

27. Cheng Z (Aaron, Greenwood BN, Pavlou PA. Location-Based Mobile Gaming and Local Depression Trends: A Study of Pokémon Go. Journal of Management Information Systems. Jan 2, 2022;39(1):68-101. [doi: 10.1080/07421222.2021.2023407]

28. Clark E. Nearly All Pokémon Go Users Meet New People While Playing. The Manifest; 2018 [cited 2026 Accessed 2026-04-14]; Available from: https://medium.com/@the_manifest/nearly-all-pok%C3%A9mon-go-users-meet-new-people-while-playing-7faee6415a7.

29. Coldewey D. To catch a Pikachu: NY Governor moves to ban sex offenders from Pokémon GO. TechCrunch; 2016 [Accessed 2025-11-27]; Available from: https://techcrunch.com/2016/08/01/to-catch-a-pikachu-ny-governor-moves-to-ban-sex-offenders-from-pokemon-go/.

30. Conditt J. Pokemon Go's mental health benefits are real. Engadget; 2016 [cited Accessed 2023-09-21]; Available from: https://www.engadget.com/2016-07-13-pokemon-go-mental-health-science.html.

31. Costello R, Smith L. An Innovative Approach of Using Mobile Gaming to Bridge Well-Being, Anxiety, Depression, and Isolation. International Journal of Adult Education and Technology. Jan 2022;13(1):1-18. URL: https://services.igi-global.com/resolvedoi/resolve.aspx?doi=10.4018/IJAET.20220101 [doi: 10.4018/IJAET.302013]

32. de Souza e Silva A, Glover-Rijkse R, Njathi A, de Cunto Bueno D. Playful mobilities in the Global South: A study of Pokémon Go play in Rio de Janeiro and Nairobi. New Media & Society. 2023;25(5). doi: https://doi.org/10.1177/14614448211016400.

33. Dunham J, Papangelis K, LaLone N, Wang Y. The player traits and gratifications of casual and hardcore players in Harry Potter: Wizards Unite, Ingress, and Pokémon GO. Behav Inf Technol. 2025;44(4):805-828. [doi: 10.1080/0144929X.2024.2345293]

34. Ellis LA, Lee MD, Ijaz K, Smith J, Braithwaite J, Yin K. COVID-19 as “Game Changer” for the Physical Activity and Mental Well-Being of Augmented Reality Game Players During the Pandemic: Mixed Methods Survey Study. J Med Internet Res. Dec 22, 2020;22(12):e25117. [doi: 10.2196/25117] [Medline: 33284781]

35. Escaravajal-Rodríguez JC. Pokémon GO and its Influence on Spanish Facebook Users. Apunts: Educació Física i Esports. 2018(133):38-49. [doi: 10.5672/apunts.2014-0983.es.(2018/3).133.03]

36. Etherington D. PokeFit gives Pokemon Go a real-time fitness dashboard on Android. TechCrunch; 2016 [Accessed 2023-10-16]; Available from: https://techcrunch.com/2016/07/29/pokefit/.

37. Evans L, Saker M. The playeur and Pokémon Go: Examining the effects of locative play on spatiality and sociability. Mobile Media & Communication. May 2019;7(2):232-247. [doi: 10.1177/2050157918798866]

38. Evans J, Evans SZ, Shank DB, Fallon QP. Motivations for Social Interaction: The Case of Pokémon Go After the Fad Ended. Soc Sci Q. Jan 2021;102(1):547-551. URL: https://onlinelibrary.wiley.com/toc/15406237/102/1 [doi: 10.1111/ssqu.12880]

39. Ewell PJ, Quist MC, Øverup CS, Watkins H, Guadagno RE. Catching more than pocket monsters: Pokémon Go’s social and psychological effects on players. J Soc Psychol. Mar 3, 2020;160(2):131-136. [doi: 10.1080/00224545.2019.1629867]

40. Faccio M, McConnell JJ. Death by Pokémon GO: The Economic and Human Cost of Using Apps While Driving. J of Risk & Insurance. Sep 2020;87(3):815-849. [doi: 10.1111/jori.12301]

41. Finco MD, Rocha RS, Fao RW, editors. Pokemon GO: a healthy game for all. Presented at: 11th European Conference on Game-Based Learning (ECGBL); Oct 5-6, 2017; Graz, Austria. URL: https://share.google/vowmbM85svlT7K4KW

42. Fisher-Reid GE. Pokémon go community day: self interest, socialization and community building. New York, United States: State University of New York at Stony Brook; 2020. URL: https://www.proquest.com/openview/a894d3c3d934c22857598e8e5b2c33de/1.pdf?pq-origsite=gscholar&amp;cbl=18750&amp;diss=y

43. Fountaine CJ, Springer EJ, Sward JR. A Descriptive Study of Objectively Measured Pokémon GO Playtime in College Students. Int J Exerc Sci. 2018;11(7):526-32. PMID: 29541340. doi: https://doi.org/10.70252/GHML5307.

44. Gabbiadini A, Sagioglou C, Greitemeyer T. Does Pokemon Go lead to a more physically active life style? Computers in Human Behavior. 2018 Jul;84:258-63. PMID: 2018-19182-026. doi: <https://dx.doi.org/10.1016/j.chb.2018.03.005>.

45. Gee LA, Subramaniam A, Muthusamy S, et al. DOES PLAYING LOCATION-BASED AUGMENTED REALITY GAME INCREASES THE LEVEL OF PHYSICAL ACTIVITY? JEBAS. 2021;9(Spl-1-GCSGD_2020):S182-S186. [doi: 10.18006/2021.9(Spl-1-GCSGD_2020).S182.S186]

46. Ghazali EM, Mutum DS, Woon MY. Multiple sequential mediation in an extended uses and gratifications model of augmented reality game Pokémon Go. INTR. Jun 3, 2019;29(3):504-528. [doi: 10.1108/IntR-12-2017-0505]

47. Ghazali E, Mutum DS, Woon MY. Exploring player behavior and motivations to continue playing Pokémon GO. ITP. Jun 3, 2019;32(3):646-667. [doi: 10.1108/ITP-07-2017-0216]

48. Giller M, Kowal T, Likus W, Brzęk A. Pokémon GO, went, gone…-physical activity level, health behaviours, and mental well-being of game users: a cross-sectional study. Healthcare (Basel). Sep 17, 2025;13(18):2334. [doi: 10.3390/healthcare13182334] [Medline: 41008464]

49. Goldbach J. Duo takes daily walks playing ‘Pokemon GO’ to improve health. Daily Titan; 2017 [Accessed 2023-09-21]; Available from: https://dailytitan.com/lifestyle/duo-takes-daily-walks-playing-pokemon-go-to-improve-health/article_de43c0ce-80f8-5264-95a2-5e7c5203bdce.html.

50. Gómez-Cuesta N, Mateo-Orcajada A, Meroño L, Abenza-Cano L, Vaquero-Cristóbal R. Adolescents’ Assessment of Several Step Tracker Mobile Applications Based on Their Previous Level of Physical Activity. Children. 2025;12:554. doi: https://doi.org/10.3390/children12050554.

51. Graells-Garrido E, Ferres L, Caro D, Bravo L. The effect of Pokémon Go on the pulse of the city: a natural experiment. EPJ Data Sci. Dec 2017;6(1):1-19. [doi: 10.1140/epjds/s13688-017-0119-3]

52. Grajek M, Olszewski Ł, Krupa-Kotara K, Białek-Dratwa A, Sas-Nowosielski K. Selected behaviors and addiction risk among users of urban multimedia games. Front Psychol. 2022;13:862891. [doi: 10.3389/fpsyg.2022.862891] [Medline: 35418911]

53. Guo Y, Peeta S, Agrawal S, Benedyk I. Impacts of Pokémon GO on route and mode choice decisions: exploring the potential for integrating augmented reality, gamification, and social components in mobile apps to influence travel decisions. Transportation (Amst). 2022;49(2):395-444. [doi: 10.1007/s11116-021-10181-9] [Medline: 33642652]

54. Hamari J, Malik A, Koski J, Johri A. Uses and gratifications of Pokémon Go: why do people play mobile location-based augmented reality games? Int J Hum–Comput Interact. May 28, 2019;35(9):804-819. [doi: 10.1080/10447318.2018.1497115]

55. Harborth D, Pape S. Exploring the hype: investigating technology acceptance factors of Pokémon GO. Presented at: 2017 IEEE International Symposium on Mixed and Augmented Reality (ISMAR); Oct 9-13, 2017; Nantes, France. [doi: 10.1109/ISMAR.2017.32]

56. Harborth D, Pape S. Hansen M, Kosta E, Nai-Fovino I, Fischer-Hübner S, editors. Privacy Concerns and Behavior of Pokémon Go Players in Germany. Springer; 2018:314-329. URL: https://link.springer.com/chapter/10.1007/978-3-319-92925-5_21

57. Hino K, Asami Y, Lee JS. Step counts of middle-aged and elderly adults for 10 months before and after the release of Pokémon GO in Yokohama, Japan. J Med Internet Res. Feb 5, 2019;21(2):e10724. [doi: 10.2196/10724] [Medline: 30720438]

58. Howe KB, Suharlim C, Ueda P, Howe D, Kawachi I, Rimm EB. Gotta catch’em all! Pokémon GO and physical activity among young adults: Difference in differences study. BMJ. 2016;355:i6270. doi: https://doi.org/10.1136/bmj.i6270.

59. Hsiao CH, Tang KY. Who captures whom – Pokémon or tourists? A perspective of the Stimulus-Organism-Response model. Int J Inf Manage. Dec 2021;61:102312. [doi: 10.1016/j.ijinfomgt.2021.102312]

60. Hsieh CY, Chen T. Effect of Pokémon GO on the Cognitive Performance and Emotional Intelligence of Primary School Students. Journal of Educational Computing Research. Dec 2019;57(7):1849-1874. [doi: 10.1177/0735633119854006]

61. Huțul TD, Karner-Huțuleac A, Huțul A. “Go-tcha!”: The social component of mental health during the Covid-19 pandemic and the benefits of playing Pokemon Go. Entertain Comput. May 2024;50:100649. [doi: 10.1016/j.entcom.2024.100649]

62. Ivan C. POKEMON go, a financial strategy or a modern fight against sedentary lifestyle? Presented at: eLSE 2017; Apr 28, 2017; Bucharest, RO. [doi: 10.12753/2066-026X-17-153]

63. Jang S, Liu Y. Continuance use intention with mobile augmented reality games Overall and multigroup analyses on Pokemon Go. Information Technology & People. 2019 May;33(1):37-55. PMID: WOS:000506929900001. doi: https://doi.org/10.1108/ITP-05-2018-0221.

64. Jenny SE, Thompson RM. Pokémon Go: Encouraging recreation through augmented reality gaming. International Journal of Technology in Teaching and Learning. 2016;12(2):112-122. URL: https://share.google/mrmTF9uuXLEoKNGsU

65. Joseph B, Armstrong DG. Potential perils of peri-Pokémon perambulation: The dark reality of augmented reality? Oxford Medical Case Reports. 2016;2016(10):265–6. doi: https://doi.org/10.1093/omcr/omw080.

66. Jumareng H, Setiawan E, Németh Z. Augmented Pokemon GO in Times of COVID-19: Does it Have any Effect on Promoting Teenagers’ Physical Activity? Teor metod fiz vihov. 2022;22(3):360-365. URL: https://tmfv.com.ua/journal/issue/view/167 [doi: 10.17309/tmfv.2022.3.09]

67. Kaczmarek LD, Misiak M, Behnke M, Dziekan M, Guzik P. The Pikachu effect: social and health gaming motivations lead to greater benefits of Pokémon GO use. Comput Human Behav. Oct 2017;75:356-363. [doi: 10.1016/j.chb.2017.05.031]

68. Kaczmarek LD, Behnke M, Dżon M. Eye problems and musculoskeletal pain in Pokémon Go players. Sci Rep. Nov 11, 2022;12(1):19315. [doi: 10.1038/s41598-022-22428-1] [Medline: 36369515]

69. Kari T, Arjoranta J, Salo M, editors. Behavior change types with pokemon GO. Presented at: 12th International Conference on the Foundations of Digital Games (FDG); Aug 14-17, 2017; Hyannis, MA, USA. [doi: 10.1145/3102071.3102074]

70. Kato TA, Teo AR, Tateno M, Watabe M, Kubo H, Kanba S. Can Pokémon GO rescue shut-ins (hikikomori) from their isolated world? Psychiatry Clin Neurosci. Jan 2017;71(1):75-76. [doi: 10.1111/pcn.12481] [Medline: 27862668]

71. Khalis A, Mikami AY. Who's gotta catch 'em all?: Individual differences in Pokemon Go gameplay behaviors. Personality and Individual Differences. 2018 Apr;124:35-8. PMID: 2018-01016-009. doi: https://dx.doi.org/10.1016/j.paid.2017.11.049.

72. Khalis A, Ferrari MA, Smit S, Ewell PJ, Mikami AY. You teach me and I’ll teach you: The role of social interactions on positivity elicited from playing Pokémon GO. Cyberpsychology (Brno). 2022;16(4). [doi: 10.5817/CP2022-4-9]

73. Kim J, Merrill K, Song H. Probing with Pokémon: Feeling of presence and sense of community belonging. Soc Sci J. Jan 2, 2020;57(1):72-84. [doi: 10.1016/j.soscij.2018.11.005]

74. Kim H, Lee HJ, Cho H, Kim E, Hwang J. Replacing Self-Efficacy in Physical Activity: Unconscious Intervention of the AR Game, Pokémon GO. Sustainability. 2018;10(6):1971. [doi: 10.3390/su10061971]

75. Kogan L, Hellyer P, Duncan C, Schoenfeld-Tacher R. A pilot investigation of the physical and psychological benefits of playing Pokémon GO for dog owners. Comput Human Behav. Nov 2017;76:431-437. [doi: 10.1016/j.chb.2017.07.043]

76. Koh HE, Oh J, Mackert M. Predictors of playing augmented reality mobile games while walking based on the theory of planned behavior: web-based survey. JMIR Mhealth Uhealth. Dec 11, 2017;5(12):e191. [doi: 10.2196/mhealth.8470] [Medline: 29229586]

77. Koivisto J, Malik A, Gurkan BG. Getting healthy by catching them all: a study on the relationship between player orientations and perceived health benefits in an augmented reality game. In: Hamari J, editor. Presented at: 52nd Hawaii International Conference on System Sciences (HICSS; Jan 8-11, 2019; Hawaii, USA. URL: https://www.researchgate.net/publication/330555937_Getting_Healthy_by_Catching_Them_All_A_Study_on_the_Relationship_Between_Player_Orientations_and_Perceived_Health_Benefits_in_an_Augmented_Reality_Game

78. Koroleva DO, Kochervey AI, Nasonova KM, Shibeko YV. The Game Pokemon Go as a Crosscultural Phenomenon. Russian Education & Society. 2016;58(12):816–27. 10.1080/10609393.2017.1353840.

79. Kosa M, Uysal A. The role of need satisfaction in explaining intentions to purchase and play in Pokémon GO and the moderating role of prior experience. Psychol Pop Media. 2021;10(2):187-200. [doi: 10.1037/ppm0000285]

80. Krittanawong C, Aydar M, Kitai T. Pokemon Go: digital health interventions to reduce cardiovascular risk. Cardiology in the Young. 2017 Oct;27(8):1625-6. PMID: WOS:000417419400026. doi: https://doi.org/10.1017/s1047951117000749.

81. Ku GC, Shang IW, Li MF. How Do Location-Based Augmented Reality Games Improve Physical and Mental Health? Evaluating the Meanings and Values of Pokémon Go Users’ Experiences through the Means-End Chain Theory. Healthcare (Basel). 2021;9(7). [doi: 10.3390/healthcare9070794] [Medline: 34202879]

82. Kumparak G. Pokémon GO introduces daily bonuses to keep players coming back. TechCrunch. 2016. URL: https://techcrunch.com/2016/11/02/pokemon-go-introduces-daily-bonuses-to-keep-players-coming-back [Accessed 2023-09-21]

83. Kurland R. Invasion of the Poké-Persuasion: Pokémon Go Users 'Play' Near Jewish Landmarks. Jewish Exponent; 2016 [Accessed 2023-09-21]; Available from: https://www.jewishexponent.com/invasion-of-the-poke-persuasion-pokemon-go-users-play-near-jewish-landmarks/.

84. Laato S, Islam A, Laine TH. Did location-based games motivate players to socialize during COVID-19. Telemat Inform. Nov 2020;54:101458. [doi: 10.1016/j.tele.2020.101458] [Medline: 34887611]

85. Laato S, Islam AKMN, Laine TH. Playing location-based games is associated with psychological well-being: an empirical study of Pokémon GO players. Behaviour & Information Technology. Jul 4, 2022;41(9):1888-1904. [doi: 10.1080/0144929X.2021.1905878]

86. Lalot F, Zerhouni O, Pinelli M. “I Wanna Be the Very Best!” agreeableness and perseverance predict sustained playing to Pokémon Go: a longitudinal study. Games Health J. Oct 2017;6(5):271-278. [doi: 10.1089/g4h.2017.0051] [Medline: 28661725]

87. Langford TW, Pribyslavska V, Barry VW. Daily Total Energy Expenditure Associated with Playing Pokémon Go. Int J Exerc Sci. 2019;12(5):1179-1186. [doi: 10.70252/CIWA3668] [Medline: 31839855]

88. Laor T. The race to escape: location-based escapism and physical activity as a motivator in the consumption of the AR game Pokémon Go. Cyberpsychol J Psychosoc Res Cyberspace. 2020;14(2). [doi: 10.5817/CP2020-2-6]

89. Laor T. Running away with the Pokémon GO gang: three consumption needs. Int J Hum-Comput Interact. Aug 27, 2022;38(14):1321-1332. [doi: 10.1080/10447318.2021.2002041]

90. Lawler R. Pokémon Go’ starts tracking steps using HealthKit and Google Fit. Engadget; 2018. URL: https://www.engadget.com/2018-11-01-pokemon-go-adventure-sync-apple-health-googlefit.html [Accessed 2023-09-21]

91. Lawler-Sagarin KA, Sagarin BJ, Pederson A. Enhanced Community Through Augmented Reality: Social Benefits of Pokémon Go. Psychol Rep. Oct 2025;128(5):3428-3440. [doi: 10.1177/00332941231197155] [Medline: 37605801]

92. Lee LH, Lin Z. Danger, nuisance, disregard: analyzing user-generated videos for augmented reality gameplay on hand-held devices. Proc ACM Hum-Comput Interact. Oct 14, 2024;8:1-33. [doi: 10.1145/3677063]

93. Lee MD. Continuation Amidst Constraint: Factors Influencing Retention and Well-Being for Players of Augmented Reality Games. University of Pennsylvania; 2021. URL: http://libproxy1.nus.edu.sg/login?url=https://www-proquest-com.libproxy1.nus.edu.sg/dissertations-theses/continuation-amidst-constraint-factors/docview/2570451568/se-2 [Accessed 2025-11-26]

94. Lee JE, Zeng N, Oh Y, Lee D, Gao Z. Effects of Pokémon GO on physical activity and psychological and social outcomes: a systematic review. J Clin Med. Apr 25, 2021;10(9):1860. [doi: 10.3390/jcm10091860] [Medline: 33922978]

95. Lemmens JS, Weergang IAM. Caught them all: gaming disorder, motivations for playing and spending among core Pokémon GO players. Entertain Comput. Mar 2023;45:100548. [doi: 10.1016/j.entcom.2023.100548]

96. Li Y, Liu Y, Ye L, Sun J, Zhang J. Pokémon GO! GO! GO! The impact of Pokémon GO on physical activity and related health outcomes. Mhealth. 2021;7:51. [doi: 10.21037/mhealth-20-121] [Medline: 34345628]

97. Lindqvist AK, Castelli D, Hallberg J, Rutberg S. The Praise and Price of Pokémon GO: A Qualitative Study of Children's and Parents' Experiences. JMIR Serious Games. 2018 Jan 3;6(1):e1. PMID: 29298750. doi: https://doi.org/10.2196/games.8979.

98. Liu HX, Holopainen J, Wagner C. A design strategy for Geo AR mobile game sustainable success emphasizing game completeness. Entertain Comput. May 2023;46:100569. [doi: 10.1016/j.entcom.2023.100569]

99. Loveday P, Flow BJ, Pokémon GO. Flow and Pokémon GO: the contribution of game level, playing alone, and nostalgia to the flow state. e-JSBRB. 2017;8(2):16-28. URL: https://research.usc.edu.au/esploro/outputs/journalArticle/Flow-and-Pokémon-GO-The-Contribution/99450699602621 [Accessed 2026-06-19]

100. Ma BD, Ng SL, Schwanen T, Zacharias J, Zhou M, Kawachi I, et al. Pokémon GO and Physical Activity in Asia: Multilevel Study. Journal of Medical Internet Research. 2018;20(6):e217. doi:10.2196/jmir.9670.

101. Macheel T, Reosti J. Pokemon’s Lessons for Loyalty: Pokemon Go is more than a game - it’s a game-changer, particularly in how it is driving traffic to retail stores and bank branches. But can this effect be duplicated. ISO & Agent. 2016;5(3):32. URL: https://libproxy1.nus.edu.sg/login?url=https://www-proquest-com.libproxy1.nus.edu.sg/trade-journals/pokemons-lessons-loyalty/docview/1825285397/se-2?accountid=13876

102. Majgaard G, Larsen LJ, editors. Pokemon GO: a pervasive game and learning community. Presented at: 11th European Conference on Game-Based Learning (ECGBL); Oct 5-6, 2017; Graz, Austria. URL: https://www.researchgate.net/publication/324748247_Pokemon_GO_A_Pervasive_Game_and_Learning_Community?__cf_chl_f_tk=UfElfjroGwNOxdjeFZ8FM.D0JvOMTwb9p3vN6NwsoNg-1782918147-1.0.1.1-oCIPKqbGSdN8a5RbYjymaF_.uRPeTfLuRjbWjP3D4N8

103. Malik A, Hiekkanen K, Hussain Z, Hamari J, Johri A. How players across gender and age experience Pokémon GO? Univ Access Inf Soc. Nov 2020;19(4):799-812. [doi: 10.1007/s10209-019-00694-7]

104. Manulife Financial Corporation. Pokémon GO gets millenials moving: 60% report increased activity. 2016. URL: http://www.manulife.com/public/news/detail/0,,lang=en&artId=148887&navId=630002,00.html [Accessed 2025-11-26]

105. Marquet O, Alberico C, Adlakha D, Hipp JA. Examining motivations to play Pokémon GO and their influence on perceived outcomes and physical activity. JMIR Serious Games. Oct 24, 2017;5(4):e21. [doi: 10.2196/games.8048] [Medline: 29066423]

106. Marquet O, Alberico C, Hipp AJ. Pokémon GO and physical activity among college students. A study using Ecological Momentary Assessment. Comput Human Behav. Apr 2018;81:215-222. [doi: 10.1016/j.chb.2017.12.028]

107. Martínez-López EJ, López-Serrano S, De La Torre-Cruz M, Ruiz-Ariza A. Effects of the augmented reality game Pokémon GO on fitness and fatness in secondary school students. Health Educ J. Feb 2022;81(1):54-68. [doi: ]

108. Mateo-Orcajada A, Abenza-Cano L, Vaquero-Cristóbal R. Importance of perceived quality and usability of step tracker mobile apps for their use by adolescents. Int J Adolesc Youth. Dec 31, 2024;29(1):2389310. [doi: 10.1080/02673843.2024.2389310]

109. Mattheiss E, Hochleitner C, Busch M. Deconstructing pokemon go - an empirical study on player personality characteristics. Presented at: 12th International Conference on PERSUASIVE Technologies (PERSUASIVE; Apr 4-6, 2017; Amsterdam, Netherlands. [doi: 10.1007/978-3-319-55134-0_7]

110. Mejia CR, Mena LS, Mogollón CA, Figueroa-Romero R, Hernández-Calderón EN, Aguilar-Fernández AM, et al. Compulsive gaming in secondary school students from five peruvian cities: Usage and addiction to the Pokémon GO game. Electronic Journal of General Medicine. 2019;16(5):em164. 10.29333/ejgm/114664.

111. Meneley A. Walk This Way: Fitbit and Other Kinds of Walking in Palestine. Cult Anthropol. 2019;34(1):130-154. [doi: 10.14506/ca34.1.11]

112. Meschtscherjakov A, Trosterer S. Persuasive effects of pokemon GO game-design elements. Presented at: 12th International Conference on PERSUASIVE Technologies (PERSUASIVE); Apr 4-6, 2017; Amsterdam, The Netherlands. [doi: 10.1007/978-3-319-55134-0_19]

113. Militello LK, Hanna N, Nigg CR. Pokémon GO within the context of family health: retrospective study. JMIR Pediatr Parent. Oct 3, 2018;1(2):e10679. [doi: 10.2196/10679] [Medline: 31518294]

114. Mukhra R, Baryah N, Krishan K, Kanchan T. “Pokémon Go” and ethical considerations—are Pokémons poking us enough? Journal of Information Ethics. 2019;28(2):117-24.

115. Muramatsu KI, Ohsaka H, Takahashi N, Yanagawa Y. Multiple Injuries Sustained When Hit by a Truck While Playing the Smartphone Game Pokemon Go. J Emerg Trauma Shock. 2019;12(2):165-166. [doi: 10.4103/JETS.JETS_131_18] [Medline: 31198288]

116. Narayan U. . M.S. Pokémon Go and Social Anxiety: A Therapeutic Platform. Northeastern University; 2018. URL: http://libproxy1.nus.edu.sg/login?url=https://www-proquest-com.libproxy1.nus.edu.sg/dissertations-theses/pokémon-go-social-anxiety-therapeutic-platform/docview/2058145664/se-2 [Accessed 2025-11-26]

117. Nelson CR, Morris W, Gabbard JL. Design considerations for augmented reality location-based exergaming: lessons from pokémon go. Presented at: 2024 IEEE International Symposium on Mixed and Augmented Reality Adjunct (ISMAR-Adjunct); Oct 21-25, 2024; Bellevue, WA, USA. [doi: 10.1109/ISMAR-Adjunct64951.2024.00056]

118. Nemet D. Childhood Obesity, Physical Activity, and Exercise. Pediatr Exerc Sci. Feb 2017;29(1):60-62. [doi: 10.1123/pes.2017-0004] [Medline: 28271810]

119. Nigg CR, Mateo DJ, An J. Pokémon GO May Increase Physical Activity and Decrease Sedentary Behaviors. Am J Public Health. Jan 2017;107(1):37-38. [doi: 10.2105/AJPH.2016.303532] [Medline: 27854536]

120. Nikou S, Tarvoll J, Oorni A. Impact of playing pokemon go on wellness. Presented at: 51st Annual Hawaii International Conference on System Sciences (HICSS); Jan 2-6, 2018; Hilton Waikoloa Village, Hawaii, USA. [doi: 10.24251/HICSS.2018.240]

121. Ojelabi A. Examining the Impacts of Pokémon Go on Physical Health and Social Interaction among College Students. M.S.] Michigan; 2018. URL: http://libproxy1.nus.edu.sg/login?url=https://www-proquest-com.libproxy1.nus.edu.sg/dissertations-theses/examining-impacts-pokémon-go-on-physical-health/docview/2019183540/se-2 [Accessed 2026-11-26]

122. Ono S, Ono Y, Michihata N, Sasabuchi Y, Yasunaga H. Effect of Pokémon GO on incidence of fatal traffic injuries: a population-based quasi-experimental study using the national traffic collisions database in Japan. Inj Prev. Dec 2018;24(6):448-450. [doi: 10.1136/injuryprev-2017-042503] [Medline: 29025874]

123. Orosz G, Zsila Á, Vallerand RJ, Böthe B. On the Determinants and Outcomes of Passion for Playing Pokémon Go. Front Psychol. 2018;9:316. [doi: 10.3389/fpsyg.2018.00316] [Medline: 29599735]

124. Pickett Corona S. Analyzing Spatio-Temporal Patterns of Pokémon Go Game Users and the Impacts of Users’ Physical Activities. San Diego State University; 2018. URL: https://www.proquest.com/pqdtglobal1/dissertations-theses/analyzing-spatio-temporal-patterns-pokémon-go/docview/2128702520/sem-2?accountid=1387 [Accessed 2025-11-26]

125. Ponce-Ramírez CM, Mateo-Orcajada A, Abenza-Cano L, Vaquero-Cristóbal R. Assessment of Step Tracker Mobile Applications for the Promotion of Physical Activity by Adolescents Based on Their Weight Status. Int J Telemed Appl. 2024:8038334. [doi: 10.1155/2024/8038334] [Medline: 39381367]

126. Qin Y. Attractiveness of game elements, presence, and enjoyment of mobile augmented reality games: the case of Pokémon Go. Telemat Inform. Sep 2021;62:101620. [doi: 10.1016/j.tele.2021.101620]

127. Rasche P, Schlomann A, Schäfer K, Wille M, Bröhl C. Pokémon go – an empirical user experience study. In: Theis S, editor. Advances in Intelligent Systems and Computing. 2018. URL: https://www.researchgate.net/publication/318014775_Pokemon_Go_-_an_Empirical_User_Experience_Study

128. Rasche P, Schlomann A, Mertens A. Who Is Still Playing Pokémon Go? A Web-Based Survey. JMIR Serious Games. 2017;5(2):e7. [doi: 10.2196/games.7197

129. Rauschnabel PA, Rossmann A, tom Dieck MC. An adoption framework for mobile augmented reality games: the case of Pokémon GO. Comput Human Behav. Nov 2017;76:276-286. [doi: 10.1016/j.chb.2017.07.030]

130. Richards KG, Wong KY, Khan M. Augmented reality game-related injury. BMJ Case Rep. Nov 28, 2018;11(1):e224012. [doi: 10.1136/bcr-2017-224012] [Medline: 30567080]

131. Richardson I, Hjorth L, Piera-Jimenez J. The emergent potential of mundane media: Playing Pokemon GO in Badalona, Spain. New Media & Society. 2022;24(3):667-683. [doi: 10.1177/1461444820965879] [Medline: 2022-35562-006]

132. Rowntree R, Feeney L. Smartphone and video game use and perceived effects in a community mental health service. Ir J Med Sci. Nov 2019;188(4):1337-1341. [doi: 10.1007/s11845-019-02016-5] [Medline: 31001790]

133. Ruiz-Ariza A, Casuso RA, Suarez-Manzano S, Martínez-López EJ. Effect of augmented reality game Pokémon GO on cognitive performance and emotional intelligence in adolescent young. Computers & Education. Jan 2018;116:49-63. [doi: 10.1016/j.compedu.2017.09.002]

134. Sawano T, Ozaki A, Shimada Y, et al. Pokémon GO & driving. QJM. May 2017;110(5):311-312. [doi: 10.1093/qjmed/hcx044]

135. Schade SA, Mahoney JM, Spotts AV, Greenauer N, Veerabhadrappa P. Pokémon Go did not increase step count or distance travelled among college students. Hum Mov. 2020;21(2):64-70. [doi: 10.5114/hm.2020.89916]

136. Serino M, Cordrey K, McLaughlin L, Milanaik RL. Pokémon Go and augmented virtual reality games: A cautionary commentary for parents and pediatricians. Current Opinions in Pediatrics. 2016;28(5):673-7. doi: https://doi.org/10.1097/mop.0000000000000409.

137. Shen KS. Measuring the appeal of mobility-augmented reality games, based on the innovative models of interaction: a case study. SN Appl Sci. Dec 2019;1(12). [doi: 10.1007/s42452-019-1763-y]

138. Shiau WL, Huang LC. Scale development for analyzing the fit of real and virtual world integration: an example of Pokémon Go. ITP. Mar 21, 2023;36(2):500-531. [doi: 10.1108/ITP-11-2020-0793]

139. Smith J, Lee MD, Ellis LA, Ijaz K, Yin K. Developing a novel psychographic-behavioral qualitative mapping method for exergames. IJSG. 2021;8(2):87-107. [doi: 10.17083/ijsg.v8i2.422]

140. Sobel K, Bhattacharya A, Hiniker A, Lee JH, Kientz JA, Yip JC. It wasn’t really about the pokémon": parents’ perspectives on a location-based mobile game. Presented at: CHI ’17: CHI Conference on Human Factors in Computing Systems; 1483-1496; Denver Colorado USA. 2017.

141. Sun JH. A Randomized Controlled Trial to Examine the Efficacy of Pokémon GO in Increasing Physical Activity. Drexel University; 2024. [Accessed 2025-11-26]

142. Tabacchi ME, Caci B, Cardaci M, Perticone V. Early usage of Pokémon Go and its personality correlates. Comput Human Behav. Jul 2017;72:163-169. [doi: 10.1016/j.chb.2017.02.047]

143. Tang AKY. Key factors in the triumph of Pokémon GO. Bus Horiz. Sep 2017;60(5):725-728. [doi: 10.1016/j.bushor.2017.05.016]

144. Tannemaat MR, Aziz NA. Creating dynamic virtual quarantines using “Pokémon Go” to limit infectious diseases spread. Med Hypotheses. Feb 2017;99:76-77. [doi: 10.1016/j.mehy.2017.01.004] [Medline: 28110705]

145. Tateno M, Skokauskas N, Kato TA, Teo AR, Guerrero APS. New game software (Pokémon Go) may help youth with severe social withdrawal, hikikomori. Psychiatry Res. Dec 30, 2016;246:848-849. [doi: 10.1016/j.psychres.2016.10.038] [Medline: 27817905]

146. Thompson D. A new roadway danger: Drivers chasing ’Pokemon Go. HealthDay; 2016. URL: https://medicalxpress.com/news/2016-09-roadway-danger-drivers-pokemon.html [Accessed 2025-11-26]

147. Thompson D. Pokemon Go’ Players Add 2,000 Steps a Day. HealthDay; 2017. URL: https://consumer.healthday.com/fitness-information-14/walking-health-news-288/pokemon-go-players-add-2-000-steps-a-day-720486.html [Accessed 2025-11-26]

148. Thongmak M, editor. Motives to play a mobile location-based augmented-reality game of non-adopters: the case of pokémon go. Presented at: Proceedings of the International Conference on Electronic Business (ICEB); Dec 8-12, 2019; Tyne, UK. URL: https://aisel.aisnet.org/iceb2019/46

149. Thongmak M. Determinants of intention to play Pokémon Go. Heliyon. Dec 2020;6(12):e03895. [doi: 10.1016/j.heliyon.2020.e03895] [Medline: 33364473]

150. Thongmak M. Protecting privacy in Pokémon Go: A multigroup analysis. Technol Soc. Aug 2022;70:101999. [doi: 10.1016/j.techsoc.2022.101999]

151. Tong X, Gupta A, Lo H, Choo A, Gromala D, Shaw CD. Chasing lovely monsters in the wild, exploring players’ motivation and play patterns of Pokémon GO. Presented at: CSCW ’17 Companion: Companion of the 2017 ACM Conference on Computer Supported Cooperative Work and Social Computing; Feb 25 to Mar 1, 2017; Portland, OR. [doi: 10.1145/3022198.3026331]

152. Tong X, Gromala D, Shaw CD, Gupta A. Players’ experience of an augmented reality game, pokemon go: inspirations and implications for designing pervasive health gamified applications. Presented at: 5th International Conference on Distributed, Ambient and Pervasive Interactions (DAPI) Held as Part of 19th International Conference on Human-Computer Interaction (HCI International); Jul 9-14, 2017; Vancouver, BC, Canada. [doi: 10.1007/978-3-319-58697-7_50]

153. Urwin J, Flick C. AR games as a potential source of improved mental well being: Implications for self-help and individual support. Journal of Gaming & Virtual Worlds. Oct 1, 2019;11(3):309-328. [doi: 10.1386/jgvw.11.3.309_1]

154. Vaterlaus JM, Frantz K, Robecker T. “Reliving my Childhood Dream of being a Pokémon Trainer”: an exploratory study of college student uses and gratifications related to Pokémon GO. Int J Hum–Comput Interact. 2019;35(7):596-604. [doi: 10.1080/10447318.2018.1480911]

155. Vella K, Johnson D, Cheng VWS, et al. A Sense of Belonging: Pokémon GO and Social Connectedness. Games and Culture. Sep 2019;14(6):583-603. [doi: 10.1177/1555412017719973]

156. Vincent B. Australian police urge “pokémon go” players to pay more attention. Engadget; 2016. URL: https://www.engadget.com/2016-07-06-pokemon-go-australian-police.html#:~:text=Zealous%20new%20Pok%C3%A9mon%20Go%20trainers%20around%20the%20world,nab%20the%20items%20that%20could%20be%20lurking%20there

157. Wagner-Greene VR, Wotring AJ, Castor T, Kruger J, Mortemore S, Dake JA. Pokémon GO: Healthy or Harmful? Am J Public Health. Jan 2017;107(1):35-36. [doi: 10.2105/AJPH.2016.303548] [Medline: 27925813]

158. Wang AI, Skjervold A. Health and social impacts of playing Pokémon Go on various player groups. Entertain Comput. Aug 2021;39:100443. [doi: 10.1016/j.entcom.2021.100443]

159. Wang R, Wu M. Catch them all: exploring the psychological impact of playing Pokémon Go. J Commun Technol. 2020;3(1):53-72. [doi: 10.51548/joctec-2020-004]

160. Wang SD, Dzubur E, Naya CH, Mason TB, Dunton GF. Dyadic Effects of Pokémon GO on Physical Activity and Sedentary Behavior in Mothers and Children. Int J Exerc Sci. 2022;15(5):142-151. [Medline: PMC9987425]

161. Watanabe K, Kawakami N, Imamura K, et al. Pokémon GO and psychological distress, physical complaints, and work performance among adult workers: a retrospective cohort study. Sci Rep. 2017;7(1):10758. [doi: 10.1038/s41598-017-11176-2]

162. Wattanapisit A, Saengow U, Ng CJ, Thanamee S, Kaewruang N. Gaming behaviour with Pokémon GO and physical activity: A preliminary study with medical students in Thailand. PLoS ONE. 2018;13(6):e0199813. [doi: 10.1371/journal.pone.0199813]

163. Wedell K. Dayton Daily News. 2016. URL: https://www.daytondailynews.com/news/pokemon-game-raises-security-concerns/zfxxPiYKTVngrxj2gJpZWN [Accessed 2023-09-21]

164. Wei FYF, Wang K. Location-based mobile games in mhealth: a preliminary study of pokémon go in promoting health exercising. In: Wei FYF, Wang YK, editors. Presented at: Hawaii International Conference on System Sciences; Jan 8-9, 2019; Grand Wailea, Maui, Hawaii, USA. [doi: 10.24251/HICSS.2019.508]

165. Williams RB, Slak-Valek N. Pokémon GO is serious leisure that increases the touristic engagement, physical activity and sense of happiness of players. Inf Technol Tourism. Dec 2019;21(4):515-533. [doi: 10.1007/s40558-019-00153-2]

166. Winand M, Ng A, Byers T. Pokémon “Go” but for how long?: a qualitative analysis of motivation to play and sustainability of physical activity behaviour in young adults using mobile augmented reality. Managing Sport and Leisure. Sep 3, 2022;27(5):421-438. [doi: 10.1080/23750472.2020.1810107]

167. Wingenbach TSH, Zana Y. Playing Pokemon Go: Increased Life Satisfaction Through More (Positive) Social Interactions. Front Sports Act Living. 2022;4:903848. [doi: 10.3389/fspor.2022.903848] [Medline: 35873213]

168. Wong FY. Influence of Pokémon Go on physical activity levels of university players: a cross-sectional study. Int J Health Geogr. Feb 22, 2017;16(1):8. [doi: 10.1186/s12942-017-0080-1] [Medline: 28228102]

169. Wong RSM, Ho FKW, Tung KTS, Fu KW, Ip P. Effect of Pokémon Go on Self-Harm Using Population-Based Interrupted Time-Series Analysis: Quasi-Experimental Study. JMIR Serious Games. Jun 12, 2020;8(2):e17112. [doi: 10.2196/17112] [Medline: 32530429]

170. Wong MC, Turner P, MacIntyre K, Yee KC. Pokémon-Go: Why Augmented Reality Games Offer Insights for Enhancing Public Health Interventions on Obesity-Related Diseases. Stud Health Technol Inform. 2017;241(128-33):128-133. [Medline: 28809195]

171. Wragg J, Whall R. Beames SK, Maher PT, editors. Outdoor Play Mediated through Pokémon: Facing the Snorlax. Routledge; 2024:13. [doi: 10.4324/9781003367536-38]

172. Wu JH, Robinson S, Li Q, Huang TY, Chen YC. Online gaming: The mediating role of situational engagement in facilitating player value. Journal of Electronic Commerce Research. 2021;22(4):382-402. URL: https://www.proquest.com/openview/55c45d1587cc5475deb728cee016c8e9/1?pq-origsite=gscholar&cbl=44515

173. Wulf T, Baldwin M. Being a kid again: Playing Pokemon Go contributes to well-being through nostalgia. Studies in Communication and Media. 2020;9(2):241-63. PMID: WOS:000546985300003. doi: https://doi.org/10.5771/2192-4007-2020-2-241.

174. Xian Y, Xu H, Xu H, et al. An Initial Evaluation of the Impact of Pokémon GO on Physical Activity. J Am Heart Assoc. May 16, 2017;6(5):e005341. [doi: 10.1161/JAHA.116.005341] [Medline: 28512111]

175. Yan Z, Finn K, Breton K, Merrimack College, School of Health Sciences, North Andover, MA, United States, Merrimack College, School of Health Sciences, North Andover, MA, United States, Merrimack College, School of Health Sciences, North Andover, MA, United States. Does it Promote Physical Activity? College Students’ Perceptions of Pokémon Go. Monten J Sports Sci Med. Mar 1, 2020;9(1):5-10. URL: http://www.mjssm.me/?sekcija=articles&alc=past&alv=1 [doi: 10.26773/mjssm.200301]

176. Yang C chen, Liu D. Motives Matter: Motives for Playing Pokémon Go and Implications for Well-Being. Cyberpsychol Behav Soc Netw. Jan 1, 2017;20(1):52-57. [doi: 10.1089/cyber.2016.0562]

177. Yee KC, Wong MC, Turner P. Pokémon Go: Ubiquitous Computing Delivering Better Health or Co-Incidental Health Benefits from Technology Use? A Participatory Observational Study. Stud Health Technol Inform. 2017;234:389-394. [Medline: 28186073]

178. Yip YC, Yip KH, Tsui WK. Young Adults’ Perspectives on the Implications of an Augmented Reality Mobile Game for Communities’ Public Health: A Qualitative Study. Int J Public Health. 2023;68:1605630. [doi: 10.3389/ijph.2023.1605630] [Medline: 36938302]

179. Zsila Á, Orosz G, Bőthe B, et al. An empirical study on the motivations underlying augmented reality games: the case of Pokémon GO during and after Pokémon fever. Pers Individ Dif. Oct 2018;133:56-66. [doi: 10.1016/j.paid.2017.06.024]
